# Total Synthesis of (−)-Aspidospermidine via an Enantioselective Palladium-Catalyzed Allylic Substitution Strategy

**DOI:** 10.1021/acs.orglett.4c03445

**Published:** 2024-10-31

**Authors:** Charlotte R. O’Donnell, Christian B. W. Stark

**Affiliations:** Department of Chemistry, Institute of Organic Chemistry, University of Hamburg, Martin-Luther-King-Platz 6, 20146 Hamburg, Germany

## Abstract

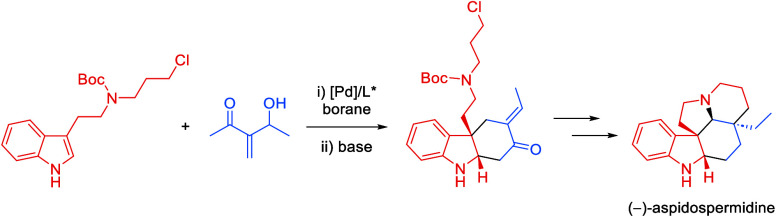

A total synthesis of (−)-aspidospermidine via an enantioselective Pd-catalyzed allylic substitution strategy is reported. This represents the first application of a Pd-catalyzed allylic substitution with a 3-substituted indole derivative in the synthesis of *Aspidosperma* alkaloids. In our synthetic route, the allylic substitution reaction was the stereo defining step. The pentacyclic framework was then constructed in a fully diastereoselective sequence. This culminated in the shortest enantioselective synthesis of aspidospermidine reported to date, in seven linear steps.

The *Aspidosperma* alkaloids are the largest family of monoterpenoid indole alkaloids, with over 240 members.^1^ Aspidospermidine (**1**) is the parent compound of the largest subclass of *Aspidosperma* alkaloids and possesses the subclass’ characteristic ABCDE pentacyclic framework with four contiguous stereocenters (Figure 1).^1a,2^ The synthesis of aspidospermidine (**1**) has consequently become the proving ground for the development of novel synthetic methodologies to access members of this alkaloid family, resulting in over 50 reported syntheses.^1b,3^ Notably, few of these syntheses employ catalytic enantioselective strategies.^4^

**Figure 1:**
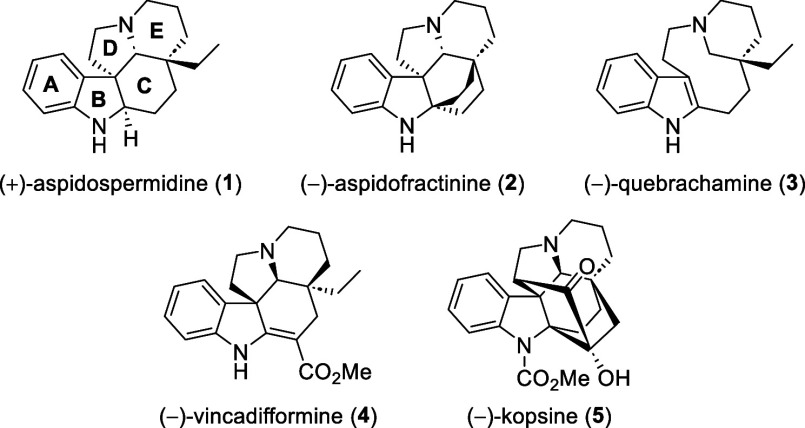
Selected *Aspidosperma* alkaloids.

Transition metal mediated allylic substitution reactions are powerful tools in catalytic asymmetric chemistry.^5^ Based on our ongoing interest in stereoselective allylic substitution methodology as well as natural product synthesis,^6^ we herein report an enantioselective Pd-catalyzed allylic substitution strategy for the total synthesis of (−)-aspidospermidine (**1**).

Our retrosynthetic analysis is outlined in Scheme 1. Aspidospermidine (**1**) would be obtained from a deoxygenation of 4-oxo-aspidospermidine **I**. The keto-group can be regarded as a residual functionality of the preceding α,β-unsaturated ketone **II**, which is anticipated as the bipolar functional handle for establishing both the D and E rings. Thus, the D/E rings in pentacycle **I** would be installed via a *N*-deprotection, *aza-*Michael addition and intramolecular enolate alkylation from tricyclic *endo*-enone **II**. The *endo*-enone functionality was planned to be established by an *exo*-to-*endo* double bond migration from *exo*-enone **III**. The *exo*-enone **III** would be obtained from the key enantioselective allylic substitution reaction between tryptamine derivative **V** and allyl cation precursor **VI**, followed by a trapping of the indolenine **IV**. The allylic substitution step should not only construct the stereo dictating quaternary carbon stereocenter, but also assemble the entire framework of aspidospermidine (**1**).

**Scheme 1:**
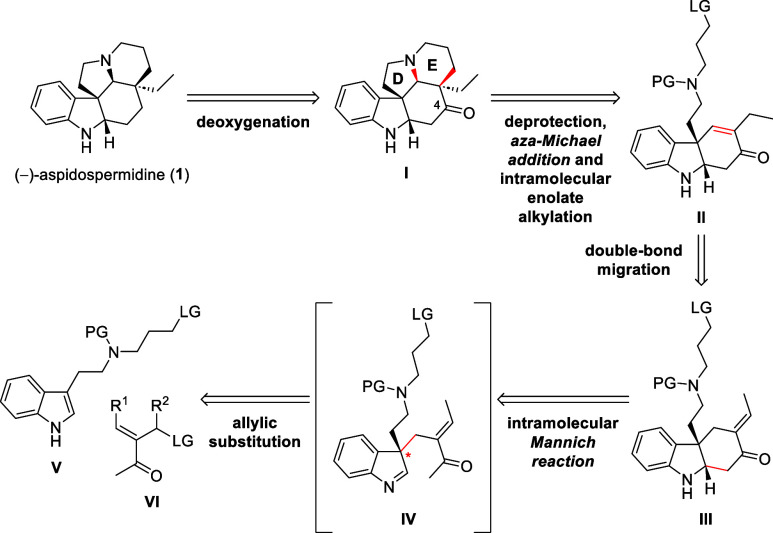
Retrosynthetic Analysis of (−)-Aspidospermidine (**1**)

Allylic substitution strategies employing 3-substituted indole derivatives as nucleophiles under Pd-catalysis have been studied in some detail.^7^ These strategies employ allylic alcohols^7a,7b,7h^ or allyl carbonates^7c−7g^ as the allyl cation precursors. Morita–Baylis–Hillman adducts are unique allylic alcohols as they form intermediary 2-acceptor substituted π-allyl-Pd-complexes. They have been rarely employed in allylic substitution reactions^8^ and to the best of our knowledge never with 3-substituted indoles as nucleophiles. However, a chemo-, regio- and stereoselective reaction of an indole nucleophile with an appropriate Morita-Baylis-Hillman electrophile would provide efficient access to densely functionalized hexahydro-1H-carbazoles and the core structure of many alkaloids including those of the *Aspidosperma* family.

For investigation into the key allylic substitution reaction, the tryptamine derivative **7** and a set of allyl cation precursors **9a**–**c** were prepared (Scheme 2).^9^ These substrates were submitted to nonstereoselective allylic substitution protocols (see the Supporting Information).^7a,7c^ From these studies, the Morita–Baylis–Hillman adduct **9a** was selected as the allyl cation precursor best suited for our strategy.^9a^

**Scheme 2:**
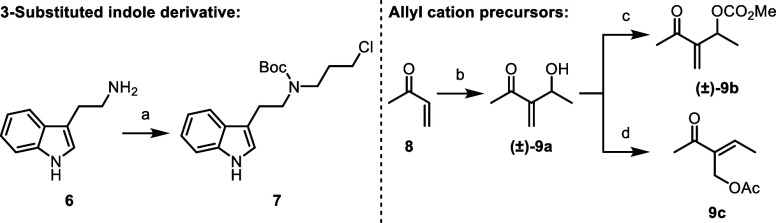
Synthesis of Substrates for the Allylic Substitution Reaction Conditions: (a) 1-bromo-3-chloropropane, 35 °C to rt, 26 h, then Boc_2_O, 60 °C, 2 h, 72%; (b) CH_3_CHO, DABCO, 0 °C to rt, 15 h, 83%; (c) methyl chloroformate, 4-DMAP, pyridine, CH_2_Cl_2_, 0 °C, 1 h, 20%; (d) PPh_3_, CH_3_COOH, DIAD, THF, 0 °C, 1 h, 41%.

We then attempted to render the allylic substitution reaction enantioselective.^7b^ From screening of chiral ligands (see the Supporting Information for more information), the DACH-naphthyl **L1** and DACH-phenyl **L2** were found to afford the enantioenriched indolenine **10** with comparable enantiomeric ratios (e.r.) (Table 1, entries 1 and 2). Interestingly, the ANDEN-phenyl ligand **L3** performed least effective (Table 1, entry 6). All reactions delivered the desired product **10** with excellent chemo- and regioselectivity. Since the DACH-phenyl ligand **L2** afforded the indolenine **10** in the highest yield, it was taken forward in the optimization studies. Increasing the steric bulk of the borane additive to 9-BBN-octyl was found to increase the e.r. (Table 1, entry 3).^6a,7b,10^ While lowering the temperature to room temperature was shown to increase both the yield and e.r. (Table 1, entry 4). However, further cooling to 4 °C was detrimental. (Table 1, entry 5). Other solvents or reagent ratios led to inferior results (Table 1, entries 7–11). Extending the reaction time had no effect on the yield of indolenine **10** (Table 1, entry 12). Finally, the optimized enantioselective conditions were shown to be scalable with the indolenine **10** obtained in a yield of 42% and an e.r. of 91:9 (Table 1, entry 13). Initial optimization of the enantioselective allylic substitution reaction was performed with the (*S*,*S*)-configured ligands. However, to synthesize the natural product (+)-aspidospermidine (**1**) we switched to the (*R*,*R*)-**L2**, based on extrapolation of the stereochemical results reported by Trost and co-workers.^7b^

**Table 1:**
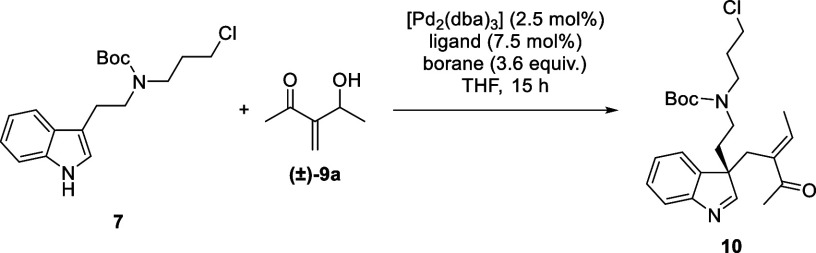
Optimization of the Conditions for the Enantioselective Allylic Substitution Reaction^a^

entry	L_n_	borane	*T* (°C)	yield (%)	e.r. (*R*:*S*)
1^b^	(*S*,*S*)-**L1**	Et_3_B	50	23	19:81
2^b^	(*S*,*S*)-**L2**	Et_3_B	50	49	26:74
3	(*S*,*S*)-**L2**	9-BBN-octyl	50	22	14:86
4	(*S*,*S*)**-L2**	9-BBN-octyl	rt	37	9:91
5	(*S*,*S*)-**L2**	9-BBN-octyl	4	18	30:70
6	(*S*,*S*)-**L3**	9-BBN-octyl	rt	26	37:63
7^c^	(*S*,*S*)-**L2**	9-BBN-octyl	rt	23	18:82
8^d^	*(S*,*S*)-**L2**	9-BBN-octyl	rt	25	14:86
9^e^	(*S*,*S*)-**L2**	9-BBN-octyl	rt	0	–
10^f^	(*R*,*R*)-**L2**	9-BBN-octyl	rt	0	–
11^g^	(*R*,*R*)-**L2**	9-BBN-octyl	rt	12	56:44
12^h^	(*R*,*R*)-**L2**	9-BBN-octyl	rt	36	90:10
13^i^	(*R*,*R*)**-L2**	9-BBN-octyl	rt	42	91:9

a Unless otherwise stated, all reactions were performed on a 0.10 mmol scale with **9a** (2.0 equiv).

b Additional **9a** (2.0 equiv) was added after 15 h; reaction time of 48 h.

c With 4.0 equiv of **9a**.

d With 1.5 equiv of **9a**.

e Reaction in PhMe.

f Reaction in CH_2_Cl_2_.

g (*R*,*R*)-**L2** (15 mol %) and Pd_2_(dba)_3_ (5 mol %).

h Reaction time of 24 h.

i Reaction scale of 1.0 mmol.

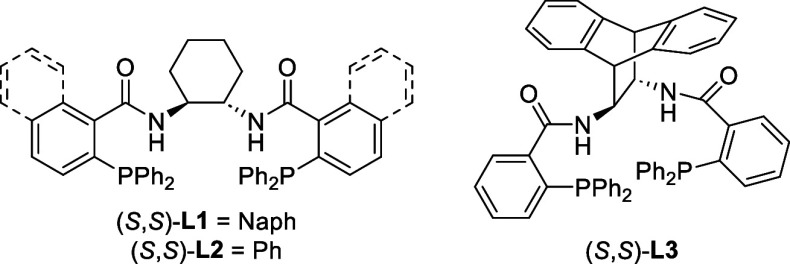

With the key Pd-catalyzed enantioselective allylic substitution successfully performed, we turned our attention to the next steps in the synthetic route (Scheme 3). Indolenine **10** was cyclized under basic conditions to produce tricycle **11** as a single diastereomer. Moreover, the allylic substitution and intramolecular Mannich reaction could also be performed as a one-pot process, albeit with diminished yield. The ensuing *exo*-to-*endo* double bond migration presented a significant challenge (see the Supporting Information). Eventually, it was found that *N*-protection of the *exo*-enone **11** followed by 1,4-hydrosilylation gave the silyl enol ether **13** in 81% over two steps.^11^ A subsequent IBX-mediated Nicolaou oxidation furnished the *N*-Boc *endo*-enone **14** together with *N*-Boc *exo*-enone **12**, which could be resubmitted to the 1,4-hydrosilylation reaction.^12^*N*-deprotection and concomitant *aza*-Michael addition proceeded in the presence of trifluoroacetic acid, furnishing the tetracyclic product **15**. The final E ring in pentacycle **16** was installed by an intramolecular enolate alkylation using *t*-BuOK. Pleasingly, the D/E ring closures could also be performed as a one-pot transformation. In which, the deprotection was performed with BF_3_•OEt_2_ and subsequent addition of *t*-BuOK induced the intramolecular enolate alkylation, affording pentacycle **16** in a yield of 71% from *endo*-enone **14**—which was higher than the yield of 56% over two separate steps. Gratifyingly, both the *aza*-Michael addition and intramolecular enolate alkylation reactions afforded only the single desired diastereomer, when performed over one- or two-steps, due to the high substrate-induced stereocontrol. A final Wolff–Kishner reduction yielded (−)-aspidospermidine (**1**). Interestingly, the unnatural enantiomer of aspidospermidine (**1**) was obtained; therefore, it was not possible to extrapolate the stereochemical results from Trost and co-workers to our system.^7b^

**Scheme 3:**
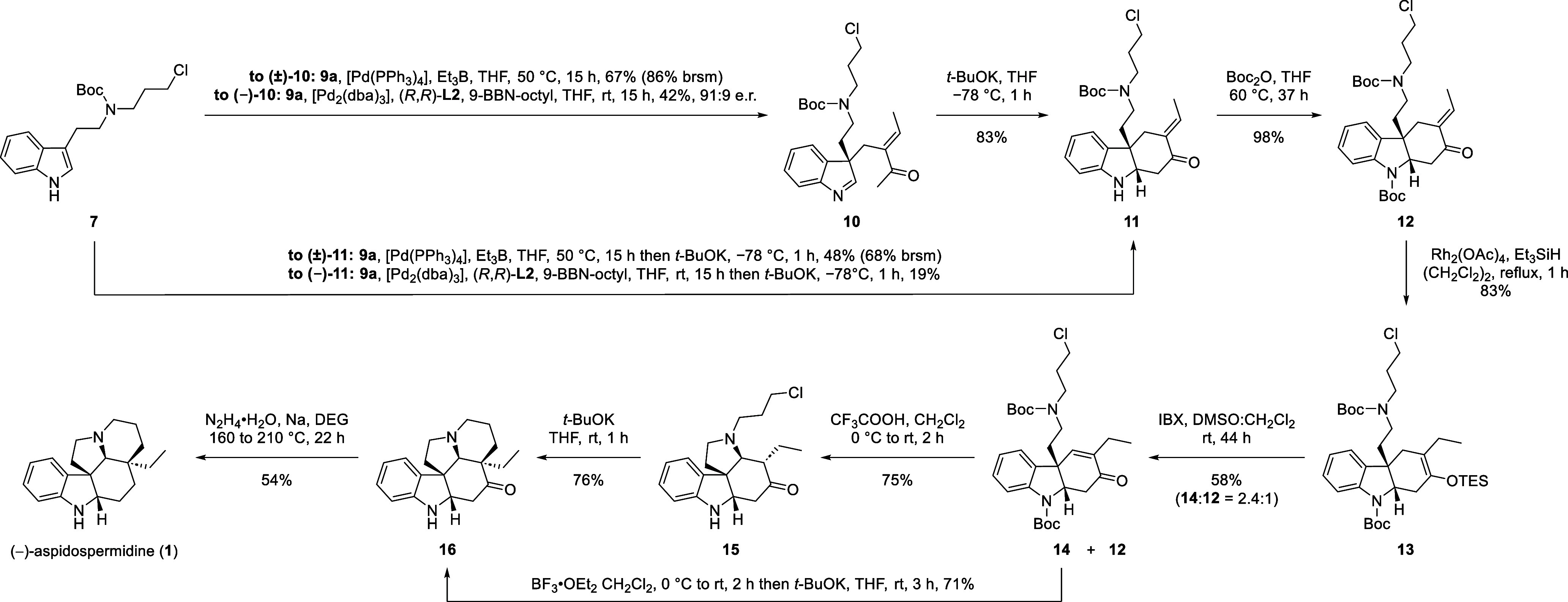
Total Synthesis of (−)-Aspidospermidine (**1**)

In conclusion, we have reported the first application of a Pd-catalyzed allylic substitution with a 3-substituted indole derivative in the synthesis of aspidospermidine (**1**) and more broadly of *Aspidosperma* alkaloids. The allylic substitution reaction was developed with high enantioselectivity (e.r. of 91:9) and acted as the stereo defining step. The remaining stereocenters of the natural product were then established under substrate control. This cumulated in the shortest enantioselective synthesis of aspidospermidine (**1**) reported to date, over seven linear steps from commercially available starting materials.^13^ We envision that our strategy could be employed for enantioselective syntheses of other members of the *Aspidosperma* alkaloid family.^14^

## Data Availability

The data underlying this study are available in the published article and its Supporting Information.

## Supporting Information Available

Experimental procedures, characterization data, NMR spectra, and chiral HPLC chromatograms (PDF)
