# Supplementary material for: Total Synthesis of (−)-Aspidospermidine via an Enantioselective Palladium-Catalyzed Allylic Substitution Strategy
Source: Org Lett. 2024 Oct 31;26(45):9689–92. doi: 10.1021/acs.orglett.4c03445 (PMC11574843; doi:10.1021/acs.orglett.4c03445)
Supplement: Supplementary file 1 — ol4c03445_si_001.pdf [file ol4c03445_si_001.pdf]

Supporting Information

Total Synthesis of (−)-Aspidospermidine via an Enantioselective Palladium-Catalysed Allylic Substitution Strategy

Charlotte R. O'Donnell and Christian B. W. Stark*

Department of Chemistry, Institute of Organic Chemistry, University of Hamburg
Martin-Luther-King-Platz 6, 20146 Hamburg, Germany

*Corresponding author: christian.stark@uni-hamburg

Table of Contents

1 **General Methods..S3**

2 **Additional Information on Non-Stereoselective Allylic Substitution Reaction S5**

3 **Additional Information on Enantioselective Allylic Substitution Reaction.............S7**

4 **Additional Information on Double Bond Migration Strategies S9**

5 **Synthetic Procedures..S11**

6 **NMR Spectra...S44**

7 **HPLC Chromatograms...S62**

8 **References..S63**

1 General Methods

Experimental Procedures, Glassware and Reagents

Unless otherwise stated, reactions were performed under anhydrous conditions, under a positive pressure of dry nitrogen and the glassware was dried with a heat gun. An oil bath was used for heating of reactions. All reagents and solvents were purchased from Thermo Fisher Scientific, Merck, VWR Chemicals, Carbolution Chemicals, BLDPharm, TCI, abcr and Grüssing and were used without further purification unless stated otherwise.

Chromatography

Reactions were monitored by thin-layer chromatography (TLC), using preconditioned plates (Macherey–Nagel ALUGRAM® Xtra SIL G UV254 or Merck Supelco TLC Aluminium oxide 60 F_{254}, neutral). Visualisation was affected by quenching of UV fluorescence ($\lambda_{254\ nm}$) and by staining with standard solutions of $KMnO_4$ and cerium molybdate followed by heating. Flash chromatography was performed using SiliaFlash® Silica Gel (particle size 40–63 μm) or Merck Millipore Aluminium oxide 90 active neutral (particle size 63–200 μm).

Nuclear Magnetic Resonance (NMR)

^{1}H NMR spectra were recorded at 400 MHz, 500 MHz or 600 MHz using a Bruker AVANCE I 400, Bruker AVANCE II 400, Bruker AVANCE III HD 400, Bruker AVANCE I 500 or Bruker AVANCE III HD 600 spectrometer respectively. Spectra were referenced using the solvent peak of $CDCl_3$ at 7.26 ppm. Coupling constants (*J*) are quoted to the nearest 0.1 Hz. Assignment of proton signals was assisted by ^{1}H-^{1}H COSY, HSQC and HMBC experiments. Assignment of stereochemistry was assisted by NOESY experiments. ^{13}C NMR spectra were recorded at 101 MHz or 151 MHz using a Bruker AVANCE I 400, Bruker AVANCE II 400, Bruker AVANCE III HD 400 or Bruker AVANCE III HD 600 spectrometer respectively. Spectra were referenced using the solvent peak of $CDCl_3$ at 77.16 ppm. Peaks are generally reported to one decimal place. Assignment of carbon signals was assisted by HSQC and HMBC experiments. Assignment of stereochemistry was assisted by ^{1}H-^{1}H NOESY experiments.

Infrared Spectra (IR)

Infrared spectra were recorded using a Bruker ALPHA-P FT-IR spectrometer as thin film samples, with Diamant-ATR. Absorption maxima (ν_{max}) are quoted in wavenumbers (cm^{-1}).

Mass Spectrometry

Mass spectra were recorded on a Thermo ISQ LT EI instrument using Electron Ionisation Gas Chromatography Mass Spectrometry (EI-GCMS). High resolution mass spectra were recorded on an Agilent 6224 ESI-TOF instrument using Electrospray Ionisation (ESI^+).

Melting Point

Melting points were measured on a Büchi Melting Point M-565 instrument.

Chiral HPLC

Analytical chiral HPLC was conducted using an Agilent 1260 Infinity II system with a Chiralpak AD-H column.

Optical Rotation

Optical rotations were measured using an A. Krüss GmbH P8000 Polarimeter, at 589 nm (sodium D line) and 20 °C with a cell path length of 1 dm and concentrations (*c*) reported in g/100 mL. Specific rotations are denoted as: (*c* in g/100 mL, solvent) $[\alpha]_{\mathrm{D}}^{20}$.

2 Additional Information on Non-Stereoselective Allylic Substitution Reaction

Table S1 Screening of conditions for the allylic substitution reaction[a]

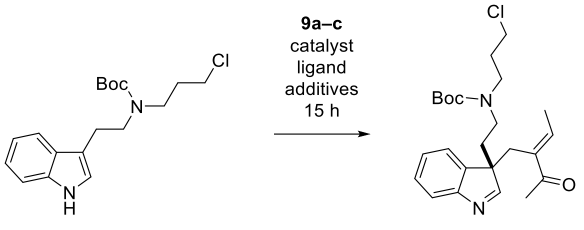

7 10

Entry	9	Catalyst, L_n and additives	Solvent	*T* [°C]	Yield [%]
1	b	$[Pd_2(dba)_3]$ $P(2\text{-furyl})_3$[b]	$(CH_2Cl)_2$	rt	0
2	a	$[Pd(PPh_3)_4]$ Et_3B[c]	THF	rt	5.8
3	a	$[Pd(PPh_3)_4]$ Et_3B[c]	THF	50	33
4	c	$[Pd(PPh_3)_4]$ Et_3B[c]	THF	50	31
**5**[d]	**a**	**$[Pd(PPh_3)_4]$ Et_3B**[c]	**THF**	**50**	**67 (86 brsm)**

[a]Unless otherwise stated, all reactions were performed on a 0.10 mmol scale with allyl cation precursor **9** (2.0 equiv.). [b]$[Pd_2(dba)_3]$ (5 mol%), $P(2\text{-furyl})_3$ (10 mol%). [c]$[Pd(PPh_3)_4]$ (5 mol%), Et_3B (3.6 equiv.). [d]Reaction scale of 1.0 mmol.

With allyl cation precursor **9b**, conditions reported by Rawal and co-workers were applied (Table S1, entry 1);[1] however, no formation of the indolenine **10** was observed. Employing conditions inspired by Tamaru and co-workers[2] with allyl cation precursor **9a** led to traces of the desired product **10** (Table S1, entry 2). At elevated temperature, the indolenine **10** was obtained in 33% yield (Table S1, entry 3). Submitting the allyl cation precursor **9c** under the same reaction conditions, afforded the indolenine **10** in a comparable yield (Table S1, entry 4).

The Morita–Baylis–Hillman adduct **9a** was selected for further use due to the ease of its preparation. Allylic substrate **9a** was prepared in a single step with a high yield of 83%. Whereas substrate **9c** was prepared in an overall yield of 34% over two steps. Furthermore, using substrate **9c** in place of **9a** in the allylic substitution reaction would have resulted in an increase in the longest linear sequence of the synthesis. Upon performing the reaction on a larger scale, product **10** was obtained in a preparatively useful yield of 67% (86 brsm) (Table S1, entry 5).

Since full conversion of the tryptamine derivative **7** was not observed under any of these conditions (Table S1), reaction optimisation through varying the equivalents of the reagents was attempted (Table S2). The ratio of the borane to the combined equivalents of the tryptamine derivative **7** and the allylic substrate **9a** was maintained at 1.2:1.0. Varying the equivalents of the allylic substrate **9a** resulted in lower yields, even with prolonged reaction times (Table S2, entries 2 and 3). Therefore, 2.0 equivalents of the allylic substrate **9a** were found to be optimal. Reducing the relative equivalents of the borane also led to a lower yield (Table S2, entry 4). Therefore, 3.6 equivalents of the borane additive were found to be optimal.

Table S2. Optimisation of reagent equivalents in the allylic substitution reaction.

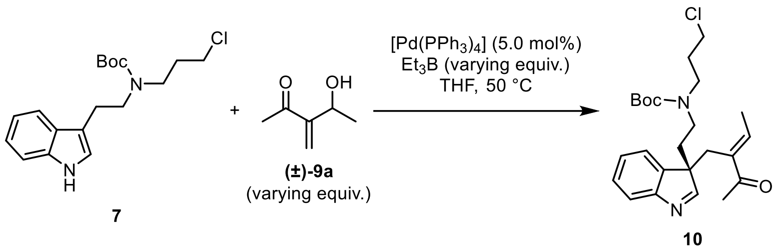

Entry	**9a** [equiv.]	Et_3B [equiv.]	Time [h]	Scale [mmol]	Yield [%]
**1**	**2.0**	**3.6**	**15**	**1.0**	**67 (86 brsm)**
2	4.0	7.2	42	1.4	56 (86 brsm)
3	1.2	2.4	40	1.2	38 (72 brsm)
4	2.0	2.4	22	0.63	40 (n.d. brsm)

3 Additional Information on Enantioselective Allylic Substitution Reaction

Table S3. Screening of chiral ligands for the enantioselective allylic substitution reaction[a]

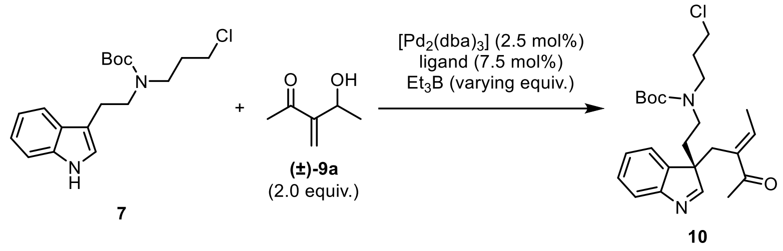

Entry	Ln	Et_3B [equiv.]	Solvent	*T* [°C]	Time [h]	Yield [%]	e.r. (*R*:*S*)
1[b]	(*S*,*S*)-**L1**	3.3	CH_2Cl_2	rt to 35	71	—	—
2[b]	(*S*,*S*)-**L2**	3.3	CH_2Cl_2	rt to 35	71	—	—
3[c]	(±)-**LS1**	2.4	THF	rt to 50	18	—	—
4[d]	(*R*)-**LS2**	2.4	THF	rt to 50	23	—	—

[a]All reactions were performed on a 0.10 mmol scale. [b]**9a** (3.0 equiv.). [c](±)-**LS1** (5.0 mol%). [d][$Pd_2(dba)_3$] (5.0 mol%), (*R*)-**LS2** (10 mol%).

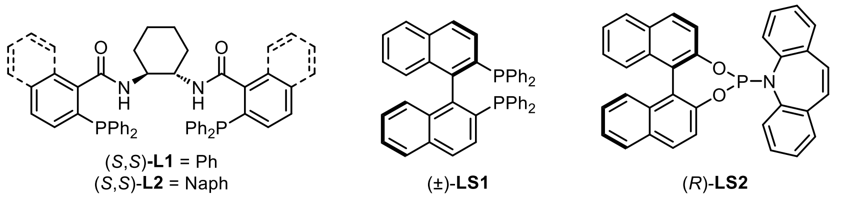

For the enantioselective allylic substitution reaction between tryptamine derivative **7** and the Morita–Baylis–Hillman adduct **9a**, conditions inspired by Trost and co-workers with the chiral ligands (*S*,*S*)-DACH-phenyl **L1** and (*S*,*S*)-DACH-naphthyl **L2** were applied (Table S3, entries 1 and 2). The reactions were initially performed at room temperature. No formation of the indolenine product **10** was observed after 23 h. The reactions were then heated to 35 °C. After 48 h at 35 °C, still no indolenine product **10** was observed. Therefore, alternative ligands were tested. BINAP (±)-**LS1** was selected, due to the potential to render the reaction enantioselective through the use of enantiopure ligand. The reaction was initially performed at room temperature; however, after two hours no formation of the indolenine product **10** was observed (Table S3, entry 3). The reaction was therefore heated to 50 °C. Unfortunately, no formation of the indolenine product **10** was observed after 16 h at 50 °C.

The Carreira phosphoramidite olefin ligand (*R*)-**LS2** was then tested as a chiral phosphoramidite olefin ligand. Hemilabile chiral phosphoramidite olefin ligands have been employed in allylic substitution reactions with 3-substituted indole derivatives and activated allylic substrates under Pd-catalysis[3] and under Ir-catalysis.[4] The reaction was initially performed at room temperature; however, after 16 h there was no formation of the indolenine product **10** (Table S3, entry 4). Therefore, the reaction was heated to 50 °C. Unfortunately, formation of the indolenine product **10** was still not observed after 7 h at 50 °C.

4 Additional Information on Double Bond Migration Strategies

Reported methods for direct *exo*-to-*endo* enone double bond migration are scarce; these employ Rh-catalysis. Unfortunately, under the conditions reported by Wakamatsu and co-workers the desired double bond migration was not observed with neither *exo*-enone **11** nor with the *N*-Boc protected substrate **12** (Scheme S1).[5] A redox strategy for the *exo*-to-*endo* double bond migration was therefore sought. The first attempt thereof was a hydrogenation-dehydrogenation sequence. The hydrogenation of the *exo*-enone **11** delivered the saturated ketone **S2**. Single-step dehydrogenation methods for ketones using Pd-catalysis[6] or the IBX-mediated Nicolaou oxidation,[7] did not yield the desired *endo*-enone **S1**. The Nicolaou oxidation instead gave the undesired but thermodynamically favoured *endo*-enone regioisomer **S3**. Other multi-step procedures to establish the desired *endo*-cyclic double bond from ketone **S2** were also unsuccessful.[8] We next envisaged a Saegusa–Ito oxidation via the thermodynamic silyl enol ether. Unfortunately, under conditions for the selective formation of the thermodynamic silyl enol ether from saturated ketone **S2**, no conversion was observed.[9] A 1,4-hydrosilylation of the *exo*-enone **11** was then proposed to selectively deliver the desired thermodynamic silyl enol ether;[10] unfortunately, this gave a complex mixture of products. However, the desired thermodynamic silyl enol ether **13** was obtained from the *N*-Boc *exo*-enone **12** in a yield of 83%. For the Saegusa–Ito oxidation of the silyl enol ether **13** to the desired *endo*-enone **14** a multitude of different conditions were investigated.[11] None of which yielded the desired *endo*-enone **14**. To our delight, the Nicolaou oxidation with silyl enol ether **13** gave the desired *N*-Boc *endo*-enone **14** in a reasonable yield together with the regioisomeric *N*-Boc *exo*-enone **12**, which could be resubmitted to the 1,4-hydrosilylation reaction.[7b]

Scheme S1 Double-bond migration strategies

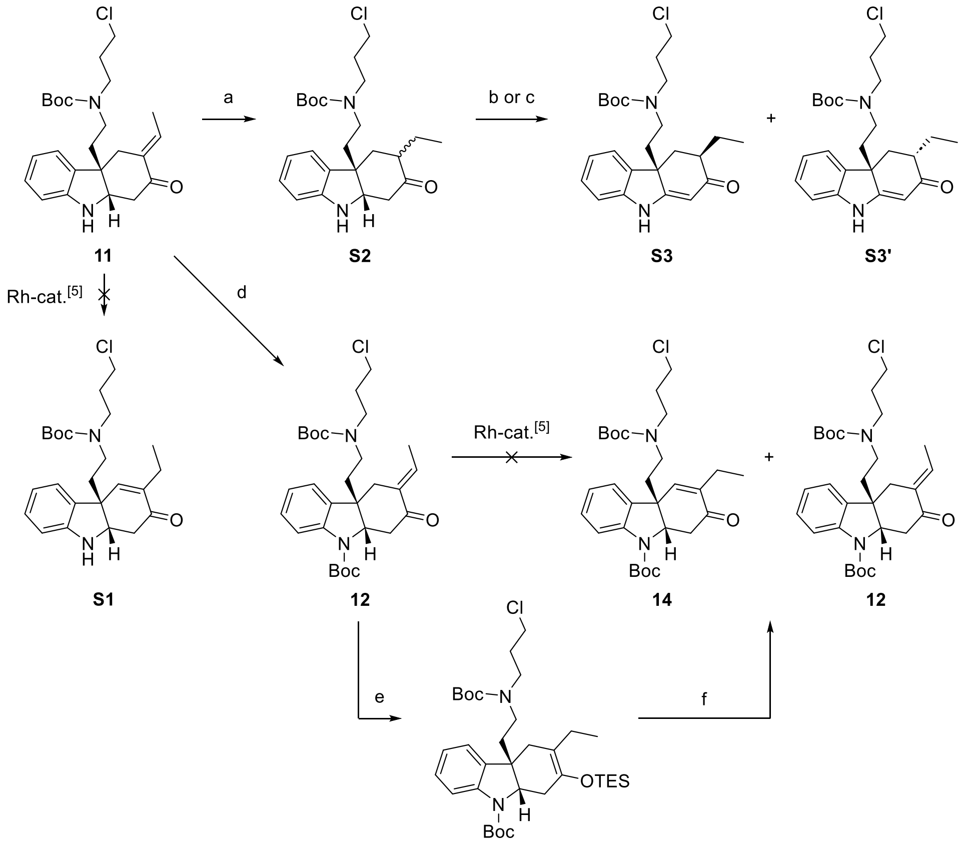

(a) Pd/C (10 wt.%), H_2 (1 atm), MeOH, rt, 24 h, 88%, d.r. 1:2; (b) IBX, PhMe:DMSO, 55 °C, 1 h, 24%, d.r. 1:7; (c) Cl_3CCOOH, nitrosobenzene, PhMe, rt, 5 h, 24%; (d) Boc_2O, 60 °C, 37 h, 98%; (e) $Rh_2(OAc)_4$, Et_3SiH, $(CH_2Cl_2)_2$, reflux, 1 h, 83 %; (f) IBX, DMSO:CH_2Cl_2, rt, 44 h, 41% **14**, 17% **12**.

5 Synthetic Procedures

5.1 Preparation of *tert*-butyl (2-(1*H*-indol-3-yl)ethyl)(3-chloropropyl)carbamate (7)

Compound **7** was prepared according to a modified literature procedure.[12]

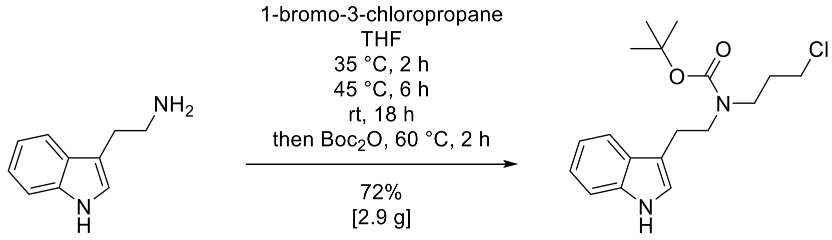

6 **7**

Tryptamine (**6**) (4.4 g, 28 mmol) was added to a flask, followed by THF (50 mL) and 1-bromo-3-chloropropane (1.2 mL, 12 mmol). The resulting mixture was stirred at 35 °C for 2 h, then at 45 °C for 6 h and at room temperature for a further 18 h. The reaction mixture was then diluted with THF (55 mL) and a solution of Boc_2O (7.6 mL, 34 mol) in THF (10 mL) was added. The resulting mixture was stirred at 60 °C for 2 h. The reaction mixture was then concentrated under reduced pressure. The residue was taken up in EtOAc and washed with H_2O. The aqueous layer was extracted with EtOAc. The combined organic extracts were dried over brine, $MgSO_4$, filtered and concentrated under reduced pressure. The crude material was purified by flash chromatography (petroleum ether 40–65/Et_2O, 7:3) to afford compound **7** as a colourless oil (2.9 g, 8.6 mmol, 72%).

The obtained spectroscopic data matched literature values.[12]

R_f = 0.32 (petroleum ether 40–65/Et_2O, 7:3);

^{1}H NMR (600 MHz, $CDCl_3$) δ = 8.32 (s, 1H), 7.66 (d, *J* = 7.0 Hz, 1H), 7.36 (d, *J* = 7.1 Hz, 1H), 7.21 (dd, *J* = 6.9 Hz, *J* = 6.9 Hz, 1H), 7.17 – 7.11 (m, 1H), 6.98 (s, 1H), 3.53 (app. s, 4H), 3.42 – 3.26 (m, 2H), 3.09 – 2.96 (m, 2H), 2.07 – 1.91 (m, 2H), 1.48 (app. d, 9H)[1] ppm;

^{13}C NMR (151 MHz, $CDCl_3$) δ = 155.8[2], 155.7[2], 136.4, 127.5, 122.1, 122.0, 119.3, 118.8, 113.1, 111.3, 79.8[2], 79.7[2], 48.7, 45.2[2], 45.1[2], 42.8[2], 42.5[2], 31.9[2], 31.5[2], 28.6[2], 28.5[2], 24.8[2], 24.1[2] ppm;

HRMS (ESI) *m/z*: $[M+Na]^+$ Calcd for $C_{18}H_{25}ClN_2O_2Na$ 359.1497; found 359.1493);

---

[1] Peak in ^{1}H NMR spectrum is split due to the presence of *N*-Boc rotamers.
[2] Peaks in ^{13}C NMR spectrum are split due to the presence of *N*-Boc rotamers.

IR (thin film) 3414 (m), 3319 (m, N–H), 2974 (m), 2929 (m), 1666 (s, C=O), 1158 (s, C–O), 738 (s, C–Cl) cm^{-1}.

5.2 Preparation of 4-hydroxy-3-methylenepentan-2-one (9a)

Compound **9a** was prepared according to a literature procedure.[13]

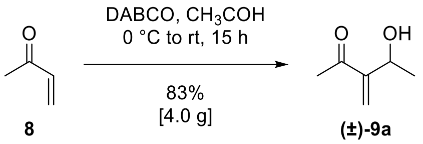

1,4-Diazabicyclo[2.2.2]octane (480 mg, 4.3 mmol) and freshly distilled acetaldehyde (2.9 mL, 51 mmol) were added to a flask. The resulting mixture was cooled to 0 °C and freshly distilled methyl vinyl ketone (**8**) (3.5 mL, 42 mmol) was added dropwise. The resulting mixture was stirred at room temperature for 15 h. The reaction mixture was then diluted with EtOAc and quenched with 1.0 M aq. HCl. The aqueous layer was extracted with EtOAc. The combined organic extracts were dried over brine, $MgSO_4$, filtered and concentrated under reduced pressure. The crude material was purified by Kugelrohr distillation (8 mbar, 80 °C to 0.6 mbar, 190 °C) to afford compound **9a** as a colourless oil (4.0 g, 35 mmol, 83%).

The obtained spectroscopic data matched literature values.[13]

5.3 Preparation of methyl (3-methylene-4-oxopentan-2-yl) carbonate (9b)

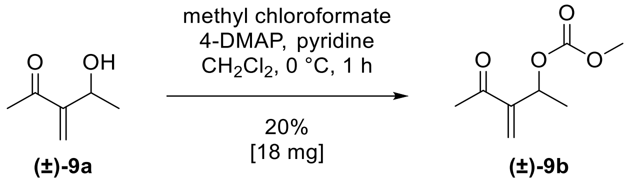

Compound **9a** (54 μL, 0.50 mmol) was added to a flask, followed by CH_2Cl_2 (0.42 mL). The resulting mixture was cooled to 0 °C and methyl chloroformate (60 μL, 0.78 mmol), 4-dimethylaminopyridine (6.1 mg, 0.050 mmol) and pyridine (50 μL, 0.62 mmol) were added. The resulting mixture was stirred for 1 h at 0 °C. The reaction mixture was then quenched with 1.0 M aq. HCl. The aqueous layer was extracted with Et_2O. The combined organic extracts were dried over brine, $MgSO_4$, filtered and concentrated under reduced pressure. The crude material was purified by flash chromatography (petroleum ether 40–65/Et_2O, 8:2) to afford compound **9b** as a colourless oil (18 mg, 0.10 mmol, 20%).

R_f = 0.33 (petroleum ether 40–65/Et_2O, 8:2);

^{1}H NMR (600 MHz, $CDCl_3$) δ = 6.13 (s, 1H), 6.07 (d, *J* = 1.2 Hz, 1H), 5.63 (qd, *J* = 6.5, 1.2 Hz, 1H), 3.76 (s, 3H), 2.35 (s, 3H), 1.38 (d, *J* = 6.5 Hz, 3H) ppm;

^{13}C NMR (151 MHz, $CDCl_3$) δ = 197.9, 154.9, 149.1, 124.7, 71.6, 54.9, 26.1, 20.8 ppm;

HRMS (ESI) *m/z*: $[M+Na]^+$ Calcd for $C_8H_{12}O_4Na$ 195.0628; Found 195.0646);

IR (thin film) 2959 (w), 2920 (w), 2851 (w), 1747 (s, C=O), 1677 (s, C=O), 1262 (s, C–O, 1076 (m) cm^{-1}.

5.4 Preparation of (*E*)-2-acetylbut-2-en-1-yl acetate (9c)

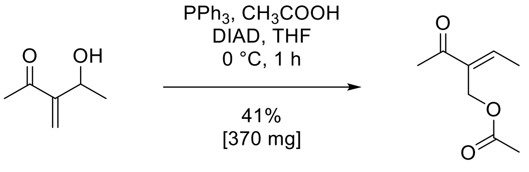

Triphenylphosphine (1.8 g, 6.9 mmol) was added to a flask, followed by compound **9a** (660 mg, 5.8 mmol), THF (47 mL) and acetic acid (0.93 mL, 16 mmol). The resulting mixture was cooled to 0 °C and diisopropyl azodicarboxylate (1.4 mL, 7.0 mmol) was added dropwise. The resulting mixture was stirred for 1 h at 0 °C. The reaction mixture was then concentrated under reduced pressure. The residue was diluted with sat. aq. $NaHCO_3$ and the aqueous layer was extracted with Et_2O. The combined organic extracts were dried over brine, $MgSO_4$, filtered and concentrated under reduced pressure. The residue was triturated (petroleum ether 40–65/Et_2O, 95:5), filtered and concentrated under reduced pressure to remove the Ph_3PO. The crude material was purified by flash chromatography (petroleum ether 40–65/EtOAc, 7:3) followed by Kugelrohr distillation (7×10^{-2} mbar, 160 °C to 6×10^{-2} mbar, 200 °C) to afford compound **9c** as a colourless oil (370 mg, 2.4 mmol, 41%).

R_f = 0.35 (petroleum ether 40–65/EtOAc, 7:3);

^{1}H NMR (400 MHz, $CDCl_3$) δ = 7.00 (q, *J* = 7.1 Hz, 1H), 4.85 (s, 2H), 2.33 (s, 3H), 2.02 (s, 3H), 1.99 (d, *J* = 7.1 Hz, 3H) ppm;

^{13}C NMR (101 MHz, $CDCl_3$) δ = 197.9, 171.0, 144.6, 137.6, 56.9, 25.7, 21.0, 15.1 ppm;

MS (EI-GCMS) *m/z* (%) = 141 (1), 113 (100), 97 (8);

IR (thin film) 2954 (m), 2923 (m), 1738 (s, C=O), 1673 (s, C=O), 1369 (m), 1231 (s, C–O), 1026 (m) cm^{-1}.

5.5 Preparation of 9-octyl-9-borabicyclo[3.3.1]nonane (9-BBN-octyl) (S5)

9-Octyl-9-borabicyclo[3.3.1]nonane (9-BBN-octyl) (**S5**) was prepared according to a literature procedure.[14]

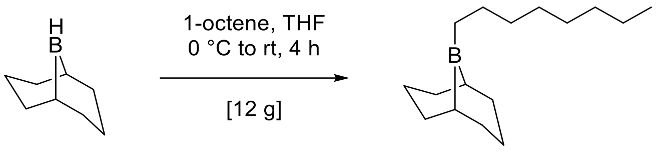

S4 **S5**

1-Octene (10 mL, 64 mmol) was added to a flask. The solution was cooled to 0 °C and 9-borabicyclo[3.3.1]nonane (9-BBN) (**S4**) (100 mL, 0.50 M in THF, 50 mmol) was added. The resulting mixture was stirred at room temperature for 4 h. The reaction was then concentrated under reduced pressure under inert conditions to give crude 9-octyl-9-borabicyclo[3.3.1]nonane (9-BBN-octyl) (**S5**) as a colourless oil (12 g) which was directly used without purification.

5.6 Preparation of *tert*-butyl (***R*,*E***)-(2-(3-(2-acetylbut-2-en-1-yl)-3*H*-indol-3-yl)ethyl)(3-chloropropyl)carbamate (10)

Non-stereoselective method:

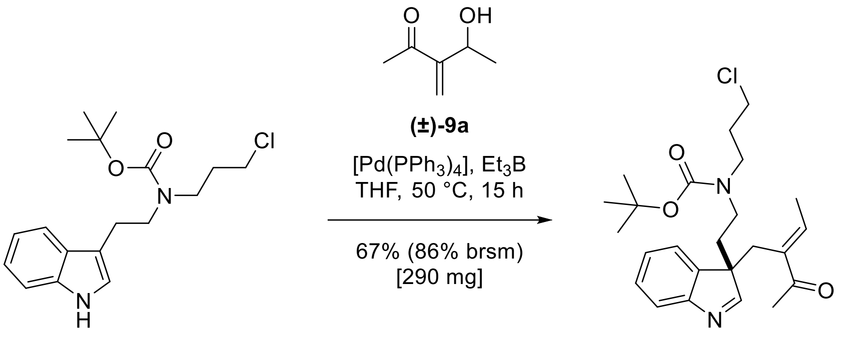

7 **10**

[Pd(PPh$_3$)$_4$] (60 mg, 0.052 mmol) was added to a flask at room temperature, followed by compound **7** (340 mg, 1.0 mmol) in THF (1.3 mL) and Et$_3$B (1.0 M in THF, 3.6 mL, 3.6 mmol). The resulting mixture was stirred at 50 °C. Compound **9a** (0.22 mL, 2.0 mmol) in THF (1.3 mL) was then added dropwise over 2.5 h. The resulting mixture was stirred at 50 °C for 15 h. The reaction mixture was then diluted with EtOAc and washed with sat. aq. NaHCO$_3$. The aqueous layer was extracted with EtOAc. The combined organic extracts were dried over brine, MgSO$_4$, filtered and concentrated under reduced pressure. The crude material was purified by flash chromatography (petroleum ether 40–65/Et$_2$O, 2:8) to afford compound **10** as a colourless oil (290 mg, 0.67 mmol, 67%, 86% brsm).

Enantioselective method:

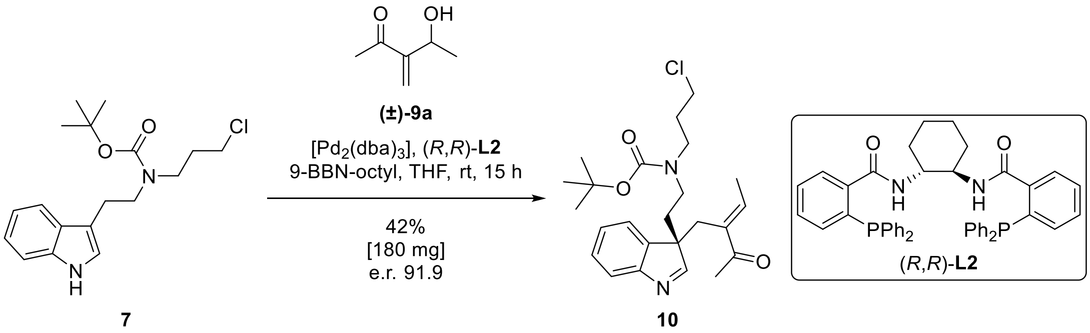

[Pd_2(dba)$_3$] (24 mg, 0.025 mmol), (*R*,*R*)-DACH phenyl Trost ligand **L2** (53 mg, 0.077 mmol) and compound **7** (340 mg, 1.0 mmol) were added to a flask at room temperature, followed by THF (4.8 mL). The resulting mixture was stirred for 15 min. 9-BBN-octyl (**S5**) (840 mg, 3.6 mmol) was added and the resulting mixture was stirred for a further 5 min. Compound **9a** (0.22 mL, 2.0 mmol) in THF (1.3 mL) was then added dropwise over 2.5 h. The resulting mixture was stirred at room temperature for 15 h. The reaction mixture was then diluted with EtOAc and washed with sat. aq. $NaHCO_3$. The aqueous layer was extracted with EtOAc. The combined organic extracts were dried over brine, $MgSO_4$, filtered and concentrated under reduced pressure. The crude material was purified by flash chromatography (petroleum ether 40–65/Et_2O, 2:8) to afford compound **10** as a colourless oil (180 mg, 0.42 mmol, 42%, 91:9 e.r.).

R_f = 0.35 (petroleum ether 40–65/Et_2O, 2:8);

^{1}H NMR (600 MHz, $CDCl_3$) δ = 7.96 – 7.82 (m, 1H), 7.49 (d, *J* = 5.7 Hz, 1H), 7.33 – 7.26 (m, 2H), 7.19 (dd, *J* = 7.5 Hz, *J* = 7.5 Hz, 1H), 6.44 (app. s, 1H), 3.38 (app. s, 2H), 3.19 – 2.94 (m, 2H)[3], 3.06 (d, *J* = 13.2 Hz, 1H), 2.94 – 2.84 (m, 1H), 2.81 – 2.45 (m, 2H), 2.34 – 2.17 (m, 1H), 2.17 – 2.07 (m, 1H), 2.00 – 1.84 (m, 3H), 1.86 – 1.68 (m, 2H), 1.49 (d, *J* = 7.1 Hz, 3H), 1.38 (s, 9H)[4] ppm;

^{13}C NMR (151 MHz, $CDCl_3$) δ = 200.1, 177.7, 155.5, 155.2, 140.5[5], 140.2[5], 139.8, 138.3, 128.2, 126.0, 123.2[5], 123.0[5], 121.1, 79.8, 61.4[5], 61.2[5], 44.9, 43.5, 42.4[5], 42.2[5], 33.9[5], 33.4[5], 31.6[5], 31.3[5], 30.4, 28.5, 25.5, 15.6 ppm;

---

[3] Overlapping signals therefore not possible to accurately integrate, the integrals shown are corrected.
[4] Corrected integral.
[5] Peaks in ^{13}C NMR spectrum are split due to the presence of *N*-Boc rotamers.

HRMS (ESI) *m*/*z*: $[M+H]^+$ Calcd for $C_{24}H_{34}ClN_2O_3$433.2252; Found 433.2263; $[M+Na]^+$ Calcd for $C_{24}H_{33}ClN_2O_3Na$ 455.2072; Found 455.2088;

IR (thin film) 2954 (s), 2922 (s), 2869 (s), 2853 (s), 1691 (s, C=O), 1670 (s, C=O), 1458 (s), 1161 (s, C–O), 737 (s, C–Cl) cm^{-1};

$[\boldsymbol{\alpha}]_{\boldsymbol{D}}^{\mathbf{20}}$ –37 (*c* 0.50, $CHCl_3$);

HPLC (Chiralpak AD-H, *n*-hexane/*i*-PrOH (9:1), 0.5 $mL\cdot min^{-1}$, λ 254 nm): t_{Rmajor} = 36.229 min, t_{Rminor} = 38.092 min.

5.7 Preparation of *tert*-butyl (3-chloropropyl)(2-((4a*R*,9a*S*,*E*)-3-ethylidene-2-oxo-1,2,3,4,9,9a-hexahydro-4a*H*-carbazol-4a-yl)ethyl)carbamate (11)

Route A

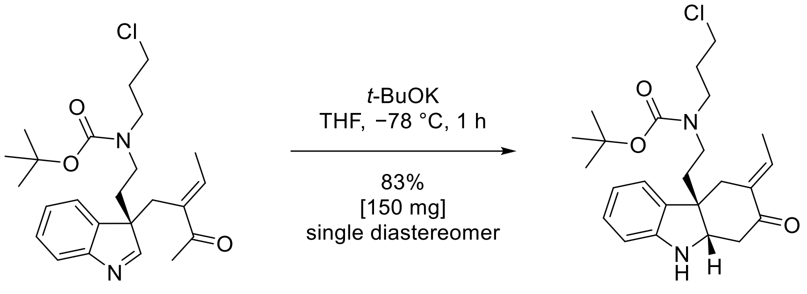

t-BuOK (79 mg, 0.71 mmol) was added to a flask, followed by THF (4.4 mL). The resulting mixture was cooled to −78 °C and compound **10** (180 mg, 0.42 mmol) in THF (4.0 mL) was added dropwise. The solution was stirred at −78 °C for 1 h. The reaction mixture was allowed to warm to room temperature and then quenched with sat. aq. NH_4Cl. The aqueous layer was extracted with EtOAc. The combined organic extracts were dried over brine, $MgSO_4$, filtered and concentrated under reduced pressure. The crude product was purified by flash chromatography (petroleum ether 40–65/Et_2O, 6:4) to afford compound **11** as a viscous colourless oil (150 mg, 0.35 mmol, 83%, single diastereomer).

Route B

Non-stereoselective:

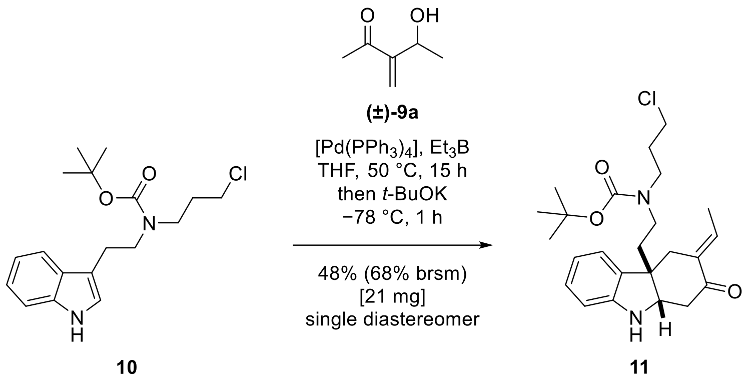

[$Pd(PPh_3)_4$] (7.2 mg, 6.2 μmol) was added to a flask, followed by compound **7** (35 mg, 0.10 mmol) in THF (0.25 mL) and Et_3B (1.0 M in THF, 0.36 mL, 0.36 mmol). The resulting mixture was stirred at 50 °C. Compound **9a** (22 μL, 0.20 mmol) in THF (0.25 mL) was then added dropwise over 2.5 h. The resulting mixture was stirred at 50 °C for 15 h. The resulting mixture was cooled to −78 °C and *t*-BuOK (65 mg, 0.58 mmol) was added. The solution was stirred at −78 °C for 1 h. The reaction mixture was allowed to warm to room temperature and then quenched with sat. aq. NH_4Cl. The reaction mixture was washed with sat. aq. $NaHCO_3$. The aqueous layer was extracted with EtOAc. The combined organic extracts were dried over brine, $MgSO_4$, filtered and concentrated under reduced pressure. The crude product was purified by flash chromatography (petroleum ether 40–65/Et_2O, 4:6) to afford compound **11** as a colourless oil (21 mg, 0.48 mmol, 48%, 68% brsm, single diastereomer).

Enantioselective:

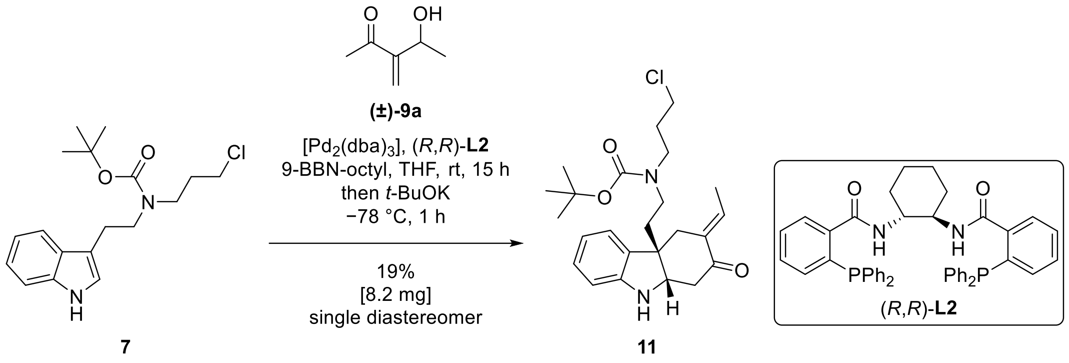

$[Pd_2(dba)_3]$ (2.8 mg, 3.1 µmol), (*R*,*R*)-DACH phenyl Trost ligand **L2** (5.9 mg, 8.5 µmol) and compound **7** (34 mg, 0.10 mmol) were added to a flask, followed by THF (0.48 mL). The resulting mixture was stirred for 15 min. 9-BBN-octyl (**S5**) (88 mg, 0.38 mmol) was added and the resulting mixture was stirred for a further 5 min. Compound **9a** (22 µL, 0.20 mmol) in THF (0.13 mL) was then added dropwise over 2.5 h. The resulting mixture was stirred at room temperature for 15 h. The resulting mixture was cooled to −78 °C and *t*-BuOK (71 mg, 0.63 mmol) was added. The solution was stirred at −78 °C for 1 h. The reaction mixture was allowed to warm to room temperature and then quenched with sat. aq. NH_4Cl. The reaction mixture was washed with sat. aq. $NaHCO_3$. The aqueous layer was extracted with EtOAc. The combined organic extracts were dried over brine, $MgSO_4$, filtered and concentrated under reduced pressure. The crude product was purified by flash chromatography (petroleum ether 40–65/Et_2O, 4:6) to afford compound **11** as a colourless oil (8.2 mg, 0.19 mmol, 19%, single diastereomer).

R_f = 0.39 (petroleum ether 40–65/Et_2O, 4:6);

^{1}H NMR (600 MHz, $CDCl_3$) δ = 6.97 (d, *J* = 7.4 Hz, 1H), 6.94 (app. s, 1H), 6.64 (dd, *J* = 6.9 Hz, *J* = 6.9 Hz, 1H), 6.59 (app. s, 1H), 6.43 (app. s, 1H), 4.13 (app. d, *J* = 67.6 Hz, 1H), 3.86 (s, 1H), 3.47 (t, *J* = 6.1 Hz, 2H), 3.42 – 3.32 (m, 1H), 3.23 (app. s, 3H), 2.97 – 2.86 (m, 1H), 2.83 (d, *J* = 14.5 Hz, 1H), 2.76 (dd, *J* = 15.3 Hz, *J* = 4.1 Hz, 1H), 2.63 – 2.50 (m, 2H), 2.08 – 1.98 (m, 1H), 1.90 (app. s, 3H), 1.54 (app. s, 3H), 1.42 (s, 9H) ppm;

^{13}C NMR (151 MHz, $CDCl_3$) δ = 199.1[6], 198.8[6], 155.4, 150.0, 134.0, 132.3, 132.0, 128.3, 122.7, 118.6, 108.9, 79.8, 60.4, 48.3, 44.9[6], 44.3[6], 44.2, 43.7, 42.6[6], 42.4[6], 39.6[6], 39.2[6], 35.8[6], 35.5[6], 31.8[6], 31.5[6], 28.5, 13.4 ppm;

HRMS (ESI) *m*/*z*: $[M+H]^+$ Calcd for $C_{24}H_{34}ClN_2O_3$ 433.2252; Found 433.2244; $[M+Na]^+$ Calcd for $C_{24}H_{33}ClN_2O_3Na$ 455.2072; Found 455.2076;

IR (thin film) 3367 (w), 2954 (s), 2923 (s), 2869 (m), 2853 (m), 1691 (s), 1634 (m), 1607 (w), 1463 (w), 1377 (m), 1366 (m), 1162 (s), 741 (s) cm^{-1};

$[\boldsymbol{\alpha}]_{\boldsymbol{D}}^{\mathbf{20}}$ −60 (*c* 0.50, $CHCl_3$).

[6] Peaks in ^{13}C NMR spectrum are split due to the presence of *N*-Boc rotamers.

5.8 Preparation of *tert*-butyl (3-chloropropyl)(2-(3-ethyl-2-oxo-1,2,3,4,9,9a-hexahydro-4a*H*-carbazol-4a-yl)ethyl)carbamate (S2)

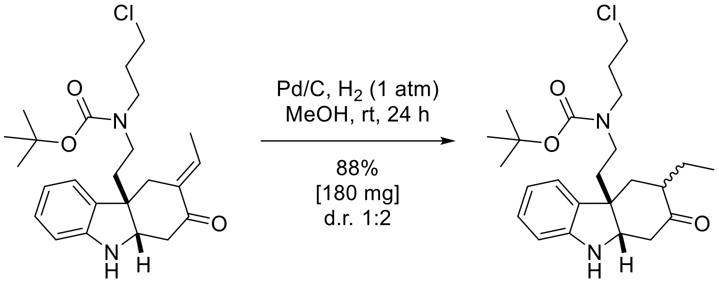

11 **S2**

Palladium 10 wt.% on activated carbon (17 mg) was added to a flask, followed by compound **11** (210 mg, 0.48 mmol) in MeOH (4.8 mL). The flask was evacuated and flushed with hydrogen. The resulting mixture was vigorously stirred under a hydrogen atmosphere (1 atm) at room temperature for 24 h. The reaction mixture was then filtered through Celite and concentrated under reduced pressure. The crude product was purified by flash chromatography (petroleum ether 40–65/Et_2O, 4:6) to afford an inseparable diastereomeric mixture of compound **S2** as a colourless oil (180 mg, 0.42 mmol, 88%, d.r. 1:2)[7].

R_f = 0.33 (petroleum ether 40–65/Et_2O, 4:6);

Major diastereomer:

^{1}H NMR (400 MHz, $CDCl_3$, 50 °C) δ = 7.09 – 6.98 (m, 2H)[8], 6.77 – 6.69 (m, 1H)[8], 6.59 (d, *J* = 7.6 Hz, 1H), 3.99 (app. s, 1H), 3.85 (s, 1H)[8], 3.49 (t, *J* = 6.4 Hz, 2H)[8], 3.30 – 3.22 (m, 2H)[8], 3.42 – 3.19 (m, 1H)[8], 3.11 – 2.89 (m, 1H)[8], 2.57 (dd, *J* = 5.9, 2.1 Hz, 2H), 2.35 – 2.20 (m, 1H), 2.17 – 1.83 (m, 4H)[8], 1.79 – 1.69 (m, 1H), 1.44 (s, 9H)[8], 1.31 – 1.21 (m, 1H), 0.88 (dd, *J* = 7.4 Hz, *J* = 7.4 Hz, 3H) ppm;

^{13}C NMR (101 MHz, $CDCl_3$, 50 °C) δ = 211.3, 155.4, 148.9, 134.6, 128.2, 122.9, 119.3, 109.9, 79.8, 63.5, 47.3, 46.3, 45.05, 44.2, 44.0, 42.5, 39.2, 36.1, 31.8, 28.6, 22.6, 11.5 ppm;

---

[7] Diastereomeric ratio was determined from the ^{1}H spectrum of the crude product.

[8] Overlapping signals of major and minor diastereomer therefore not possible to accurately integrate, the integrals shown are corrected.

Minor diastereomer:

^{1}H NMR (400 MHz, $CDCl_3$, 50 °C) δ = 7.09 – 6.98 (m, 2H)[8], 6.77 – 6.69 (m, 1H)[8], 6.51 (d, J = 7.8 Hz, 1H), 4.17 (app. s, 1H), 3.85 (s, 1H)[8], 3.49 (t, J = 6.4 Hz, 2H)[8], 3.30 – 3.22 (m, 2H)[8], 3.42 – 3.19 (m, 1H)[8], 3.11 – 2.89 (m, 1H)[8], 2.63 (dd, J = 15.9, 3.9 Hz, 1H), 2.47 (dd, J = 15.9, 3.2 Hz, 1H), 2.17 – 1.83 (m, 4H)[8], 1.68 – 1.57 (m, 1H), 1.44 (s, 9H)[8], 1.42 – 1.33 (m, 1H), 0.78 (dd, J = 7.5 Hz, J = 7.5 Hz, 3H) ppm;

^{13}C NMR (101 MHz, $CDCl_3$, 50 °C) δ = 212.8[9], 155.4, 150.4, 132.5[9], 128.5, 123.3, 119.1, 109.2, 79.8, 60.9, 47.2, 45.9, 45.09, 44.2, 43.6, 42.5, 39.4[10], 36.1[10], 31.8, 28.6, 22.9, 10.9 ppm;

HRMS (ESI) m/z: $[M+H]^+$ Calcd for $C_{24}H_{36}ClN_2O_3$ 435.2409; Found 435.2387; $[M+Na]^+$ Calcd for $C_{24}H_{35}ClN_2O_3Na$ 457.2228; Found 457.2216;

IR (thin film) 3354 (w), 2958 (s), 2925 (s), 2870 (m), 1688 (s), 1608 (w), 1481 (m), 1466 (m), 1416 (m), 1366 (m), 1161 (s), 744 (s) cm^{-1}.

---

[9] Weak, but visible in HMBC.
[10] Weak, but visible in HSQC.

5.9 Preparation of 2-Iodoxybenzoic acid (IBX) (S7)

2-Iodoxybenzoic acid (IBX) (**S7**) was prepared according to a literature procedure.[15]

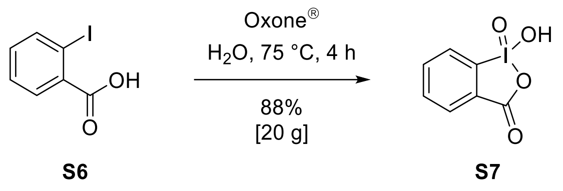

Oxone® (110 g, 180 mmol) was added to a flask, followed by 2-iodobenzoic acid (**S6**) (20 g, 81 mmol) and H_2O (400 mL). The resulting mixture was vigorously stirred at 75 °C for 4 h. The reaction mixture was then cooled to 0 °C and filtered. The crude product was washed with H_2O (100 mL) and acetone (2 × 10 mL) to afford 2-iodoxybenzoic acid (IBX) (**S7**) as a white solid (20 g, 71 mmol, 88%).

The obtained spectroscopic data matched literature values.[16]

5.10 Preparation of *tert*-butyl (3-chloropropyl)(2-(3-ethyl-2-oxo-2,3,4,9-tetrahydro-4a*H*-carbazol-4a-yl)ethyl)carbamate (S3)

Route A

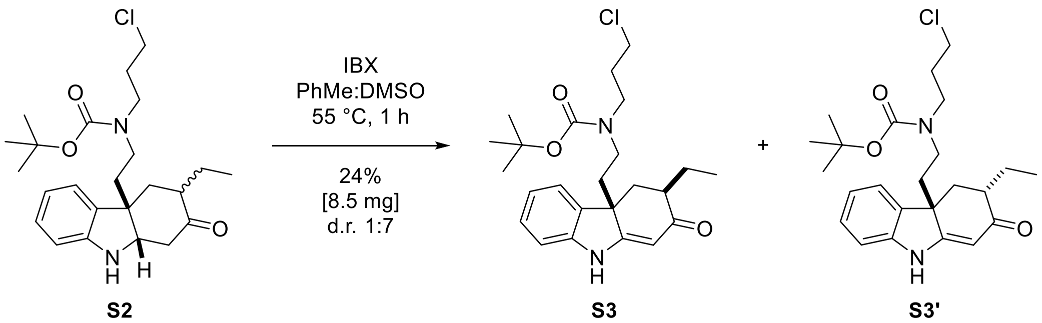

2-Iodoxybenzoic acid (IBX) (**S7**) (48 mg, 0.17 mmol) was added to a flask, followed by compound **S2** (37 mg, 0.085 mmol) in PhMe:DMSO (0.57 mL: 0.29 mL). The resulting mixture was heated at 55 °C for 1 h. The reaction mixture was then diluted with aq. $NaHCO_3$ (5%). The aqueous layer was extracted with Et_2O. The combined organic extracts were concentrated under reduced pressure. The residue was taken up in Et_2O and filtered through Celite. The filtrate was washed with sat. aq. $NaHCO_3$ and H_2O, dried over brine, $MgSO_4$, filtered and concentrated under reduced pressure. The crude product was purified by flash chromatography (petroleum ether 40–65/EtOAc, 4:6) to afford a separable diastereomeric mixture of compound **S3** as a colourless oil (8.5 mg, 0.020 mmol, 24%, d.r. 1:7)[11].

[11] Diastereomeric ratio was determined from the ^{1}H spectrum of the crude product.

Route B

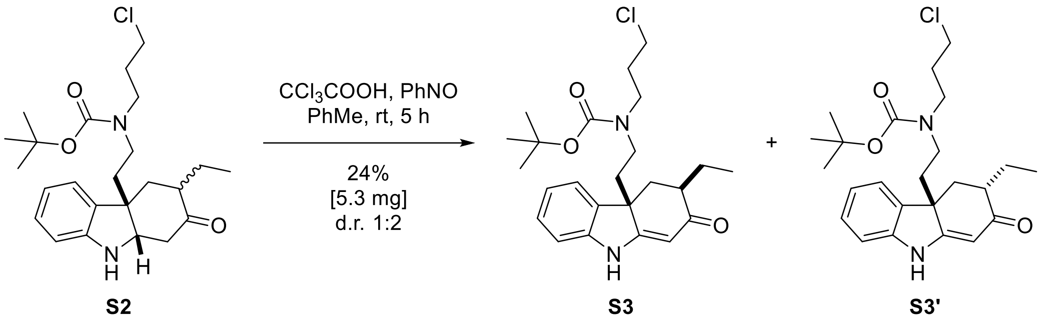

Trichloroacetic acid (27 mg, 0.17 mmol) and nitrosobenzene (17 mg, 0.16 mmol) were added to a flask, followed by compound **S2** (22 mg, 0.051 mmol) in PhMe (0.50 mL). The resulting mixture was stirred at room temperature for 5 h. The reaction mixture was then diluted with sat. aq. $NaHCO_3$ and the aqueous layer was extracted with EtOAc. The combined organic extracts were dried over brine, $MgSO_4$, filtered and concentrated under reduced pressure. The crude product was purified by column chromatography (petroleum ether 40–65/EtOAc, 4:6) to afford a separable diastereomeric mixture of compound **S3** as a colourless oil (5.3 mg, 12 μmol, 24%, d.r. 1:2)[12].

R_f = 0.39 major diastereomer, 0.25 minor diastereomer (petroleum ether 40–65/EtOAc, 4:6);

Major diastereomer[13]:

^{1}H NMR (400 MHz, $CDCl_3$, 50 °C) δ = 7.65 (s, 1H), 7.25 – 7.17 (m, 2H), 6.99 (ddd, *J* = 7.5, 7.5, 1.0 Hz, 1H), 6.87 (d, *J* = 7.8 Hz, 1H), 5.42 (s, 1H), 3.43 (t, *J* = 6.4 Hz, 2H), 3.24 – 3.06 (m, 2H), 3.06 – 2.73 (m, 2H)[14], 2.59 – 2.46 (m, 2H), 2.11 – 1.91 (m, 3H)[14], 1.85 (tt, *J* = 6.6 Hz, 2H), 1.80 – 1.68 (m, 1H), 1.66 – 1.48 (m, 1H), 1.37 (s, 9H), 0.94 (dd, *J* = 7.5, 7.5 Hz, 3H) ppm;

^{13}C NMR (101 MHz, $CDCl_3$, 50 °C) δ = 198.5[15], 173.3[15], 155.4, 143.9, 134.5, 128.6, 123.0, 122.1, 109.9, 97.8, 80.0, 48.3, 44.9[16], 44.0[16], 42.6, 42.4, 38.5[16], 35.4, 31.7, 28.6, 24.9, 11.1 ppm;

HRMS (ESI) *m/z*: $[M+H]^+$ Calcd for $C_{24}H_{34}ClN_2O_3$ 433.2252; Found 433.2236; $[M+Na]^+$ Calcd for $C_{24}H_{33}ClN_2O_3Na$ 455.2072; Found 455.2065;

---

[12] Diastereomeric ratio was determined from the ^{1}H spectrum of the crude product.
[13] Only the major diastereomer is described, due to the small quantity of the minor diastereomer obtained.
[14] Overlapping signals therefore not possible to accurately integrate, the integrals shown are corrected.
[15] Weak, but visible in HMBC.
[16] Weak, but visible in HSQC.

IR (thin film) 3185 (w, N–H), 2963 (m), 2927 (m), 2856 (m), 1693 (s, C=O), 1627 (m), 1588 (s, N–H), 1468 (m, C=C), 1366 (m), 1194 (s, C–O), 1163 (m), 750 (m, C–Cl) cm^{-1}.

5.11 Preparation of *tert*-butyl (4a*R*,9a*S*,*E*)-4a-(2-((*tert*-butoxycarbonyl)(3-chloropropyl)amino)ethyl)-3-ethylidene-2-oxo-1,2,3,4,4a,9a-hexahydro-9*H*-carbazole-9-carboxylate (12)

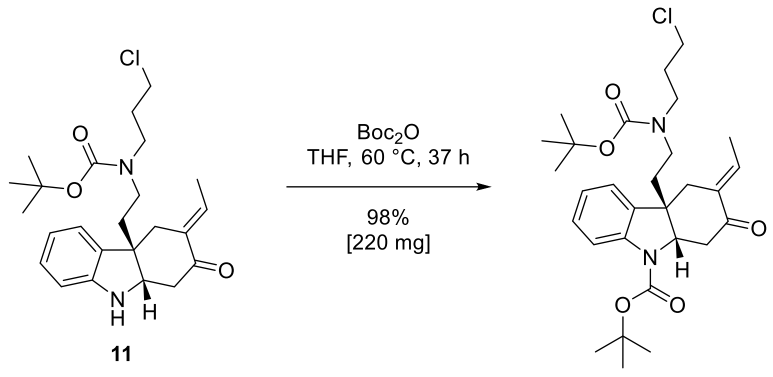

Compound **11** (180 mg, 0.42 mmol) was added to a flask followed by Boc_2O (0.24 mL, 1.0 mmol) in THF (4.2 mL). The resulting mixture was stirred at 60 °C for 37 h. The reaction mixture was then concentrated under reduced pressure. The crude product was purified by flash chromatography (petroleum ether 40–65/Et_2O, 1:1) to afford compound **12** as a white foam (220 mg, 0.41 mmol, 98%).

R_f = 0.36 (petroleum ether 40–65/Et_2O, 1:1);

^{1}H NMR (400 MHz, $CDCl_3$, 50 °C) δ = 7.64 (app. s, 1H), 7.15 (ddd, *J* = 7.2, 7.2, 1.1 Hz, 1H), 7.04 (dd, *J* = 7.2, 1.0 Hz, 1H), 6.95 (ddd, *J* = 7.4, 7.4, 0.8 Hz, 1H), 6.66 (q, *J* = 7.2 Hz, 1H), 4.52 (app. s, 1H), 3.47 (t, *J* = 6.3 Hz, 2H), 3.30 – 3.10 (m, 4H)[17], 2.97 – 2.70 (m, 4H), 1.96 (t, *J* = 8.3 Hz, 2H)[17], 1.89 (tt, *J* = 6.6 Hz, 2H)[17], 1.66 (d, *J* = 7.2 Hz, 2H), 1.57 (s, 9H), 1.43 (s, 9H) ppm;

^{13}C NMR (101 MHz, $CDCl_3$, 50 °C) δ = 197.2, 155.4, 151.9, 142.1[18], 134.8[18], 134.7, 133.9, 128.7, 123.2, 122.5, 115.7, 81.9, 80.0, 63.1, 46.7, 45.1, 43.8, 42.4, 40.2, 34.3[19], 31.8, 28.64, 28.60, 13.6 ppm;

HRMS (ESI) *m*/*z*: $[M+Na]^+$ Calcd for $C_{29}H_{41}ClN_2O_5Na$ 555.2596; Found 555.2602;

IR (thin film) 2954 (m), 2922 (m), 2853 (m), 1696 (s, C=O), 1635 (w, C=C), 1482 (s), 1378 (s), 1367 (s), 1166 (s, C–O), 739 (s, C–Cl) cm^{-1};

---

[17] Overlapping signals therefore not possible to accurately integrate, the integrals shown are corrected.
[18] Weak, but visible in HMBC.
[19] Weak, but visible in HSQC.

$[\boldsymbol{\alpha}]_{\boldsymbol{D}}^{\mathbf{20}}$ +16 (*c* 0.50, $CHCl_3$);

m.p. 52–54 °C.

5.12 Preparation of (4a*R*,9a*S*)-4a-(2-((*tert*-butoxycarbonyl)(3-chloropropyl)amino)ethyl)-3-ethyl-2-((triethylsilyl)oxy)-1,4,4a,9a-tetrahydro-9*H*-carbazole-9-carboxylate (13)

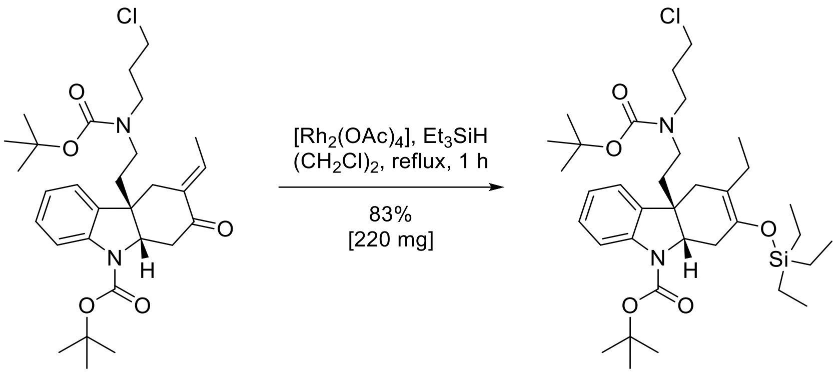

$[Rh_2(OAc)_4]$ (10 mg, 0.023 mmol) was added to a flask, followed by 1,2-dichloroethane (2.0 mL) and triethylsilane (0.17 mL, 1.1 mmol). The resulting mixture was stirred at room temperature for 10 min. Compound **12** (220 mg, 0.41 mmol) in 1,2-dichloroethane (2.0 mL) was then added. The resulting mixture was stirred at reflux for 1 h. The reaction mixture was then concentrated under reduced pressure. The crude product was purified by flash chromatography (neutral aluminium oxide, petroleum ether 40–65/EtOAc, 8:2) to afford compound **13** as a colourless oil (220 mg, 0.34 mmol, 83%).

R_f = 0.52 (neutral aluminium oxide, petroleum ether 40–65/EtOAc, 8:2);

^{1}H NMR (400 MHz, $CDCl_3$, 50 °C) δ = 7.64 (app. s, 1H), 7.12 (dd, *J* = 7.6, 7.6 Hz, 1H), 7.04 (d, *J* = 7.5 Hz, 1H), 6.94 (dd, *J* = 7.3, 7.3 Hz, 1H), 4.29 (app. s, 1H), 3.46 (t, *J* = 6.4 Hz, 2H), 3.30 – 3.11 (m, 2H)[20], 3.11 – 2.77 (m, 2H)[20], 2.56 (dd, *J* = 15.6, 5.7 Hz, 1H), 2.43 – 2.33 (m, 1H)[20], 2.33 (d, *J* = 14.9 Hz, 1H), 2.26 (d, *J* = 14.9 Hz, 1H), 2.07 – 1.94 (m, 1H), 1.94 – 1.78 (m, 5H), 1.58 (s, 9H), 1.42 (s, 9H)[20], 0.92 (t, *J* = 7.8 Hz, 9H), 0.79 (dd, *J* = 7.2, 7.2 Hz, 3H), 0.58 (q, *J* = 7.9 Hz, 6H) ppm;

^{13}C NMR (101 MHz, $CDCl_3$, 50 °C) δ = 155.5, 152.3, 142.9, 142.1, 136.9, 127.9, 122.8, 122.7, 116.0, 115.3, 81.2, 79.8, 65.7, 47.2, 45.0, 44.1, 42.5, 40.8, 37.7, 34.7, 31.8, 28.70, 28.66, 22.7, 12.2, 6.8, 5.6 ppm;

HRMS (ESI) *m/z*: $[M+Na]^+$ Calcd for $C_{35}H_{57}ClN_2O_5SiNa$ 671.3617; Found 671.3612;

---

[20] Overlapping signals therefore not possible to accurately integrate, the integrals shown are corrected.

IR (thin film) 2954 (s), 2922 (s), 2872 (m), 2853 (m), 1697 (s, C=O), 1378 (s), 1167 (s, C–O), 747 (s, C–Cl) cm^{-1};

$[\boldsymbol{\alpha}]_{\boldsymbol{D}}^{\mathbf{20}}$ +16 (*c* 0.50, $CHCl_3$).

5.13 Preparation of *tert*-butyl (4a*S*,9a*S*)-4a-(2-((*tert*-butoxycarbonyl)(3-chloropropyl)amino)ethyl)-3-ethyl-2-oxo-1,2,4a,9a-tetrahydro-9*H*-carbazole-9-carboxylate (14)

Route A

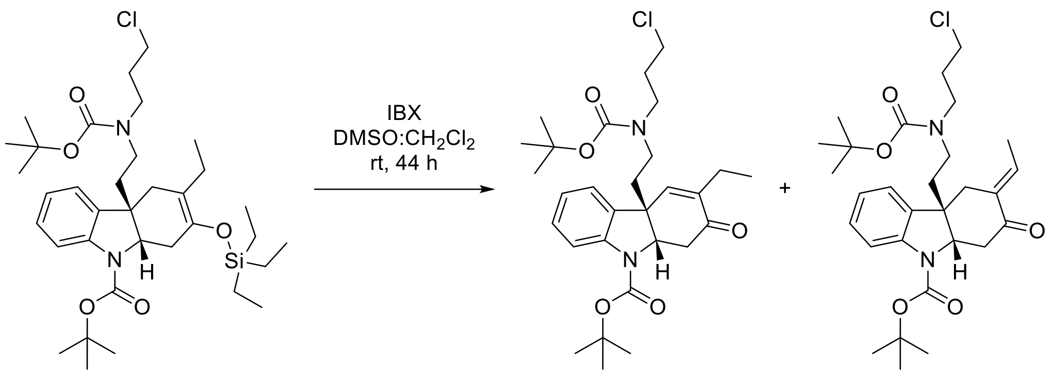

13 | **14** 41% [19 mg] | **12** 17% [8.0 mg]

2-Iodoxybenzoic acid (IBX) (**S7**) (39 mg, 0.14 mmol) was added to a flask, followed by compound **13** (57 mg, 0.088 mmol) in DMSO:CH_2Cl_2 (0.68:0.23 mL). The resulting mixture was stirred vigorously at room temperature for 44 h. The reaction mixture was then diluted with aq. $NaHCO_3$ (5%). The aqueous layer was extracted with Et_2O. The combined organic extracts were concentrated under reduced pressure. The residue was taken up in Et_2O and filtered through Celite. The filtrate was washed with sat. aq. $NaHCO_3$ and H_2O, dried over brine, $MgSO_4$, filtered and concentrated under reduced pressure. The crude product was purified by flash chromatography (petroleum ether 40–65/Et_2O, 7:3) to afford compound **14** as a colourless oil (19 mg, 0.036 mmol, 41%) and compound **12** as a white foam (8.0 mg, 0.015 mmol, 17%)[21].

R_f = 0.34 (petroleum ether 40–65/Et_2O, 7:3);

^{1}H NMR (400 MHz, $CDCl_3$, 50 °C) δ = 7.75 (d, *J* = 8.0 Hz, 1H), 7.23 (ddd, *J* = 8.1, 7.5, 1.3 Hz, 1H), 7.15 (dd, *J* = 7.5, 1.4 Hz, 1H), 7.04 (ddd, *J* = 7.5, 7.5, 1.1 Hz, 1H), 6.58 (s, 1H), 4.63 (dd, *J* = 8.8, 6.0 Hz, 1H), 3.49 (t, *J* = 6.3 Hz, 2H), 3.30 – 3.20 (m, 2H), 3.20 – 3.04 (m, 2H)[22], 2.95

[21] The highest yield of compound **14** was obtained when the oxidation was performed with freshly prepared IBX on the scale as reported above; however, when the IBX was not freshly prepared and the reaction was performed on a larger scale 2.0 equiv. of IBX were required and the reaction was performed at 55 °C with a slightly lower yield of compound **14** of 32%.

[22] Overlapping signals therefore not possible to accurately integrate, the integrals shown are corrected.

(dd, J = 15.5, 6.0 Hz, 1H), 2.54 (dd, J = 15.5, 9.9 Hz, 1H), 2.36 – 2.16 (m, 2H), 1.98 – 1.83 (m, 4H), 1.58 (s, 9H)[23], 1.42 (s, 9H), 1.05 (t, J = 7.4 Hz, 3H) ppm;

^{13}C NMR (101 MHz, $CDCl_3$, 50 °C) δ = 196.8, 155.3, 152.0, 141.8, 140.6, 133.7[24], 128.9, 123.4, 123.0, 116.2, 82.0, 80.1, 63.1, 47.0, 45.1, 43.9, 42.5, 40.6, 39.7[25], 31.8, 28.62, 28.60, 22.6, 12.8 ppm;

HRMS (ESI) *m*/*z*: $[M+Na]^+$ Calcd for $C_{29}H_{41}ClN_2O_5Na$ 555.2596; Found 555.2634;

IR (thin film) 2956 (m), 2923 (m), 2869 (m), 2853 (m), 1686 (s), 1479 (m), 1389 (m), 1367 (m), 1163 (s), 753 (m) cm^{-1};

$[\boldsymbol{\alpha}]_{\boldsymbol{D}}^{\mathbf{20}}$ +55 (*c* 0.50, $CHCl_3$).

[23] Corrected integral.
[24] Weak, but visible in HMBC.
[25] Weak, but visible in HSQC.

5.14 Preparation of (3a*S*,4*S*,6a*S*,11b*S*)-3-(3-chloropropyl)-4-ethyl-2,3,3a,4,6a,7-hexahydro-1*H*-pyrrolo[2,3-*d*]carbazol-5(6*H*)-one (15)

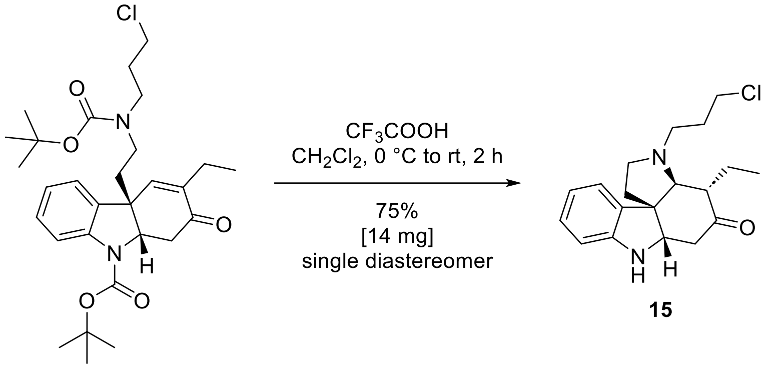

Compound **14** (30 mg, 0.056 mmol) in CH_2Cl_2 (0.57 mL) was added to a flask. The solution was cooled to 0 °C and trifluoroacetic acid (90 µL, 1.2 mmol) was added dropwise. The resulting mixture was stirred at room temperature for 2 h. The reaction mixture was then quenched with sat. aq. NH_4Cl. The aqueous layer was extracted with CH_2Cl_2. The combined organic extracts were dried over brine, $MgSO_4$, filtered and concentrated under reduced pressure. The crude product was purified by flash chromatography (petroleum ether 40–65/Et_2O/Et_3N, 3:7:0.1) to afford compound **15** as a white solid (14 mg, 0.042 mmol, 75%, single diastereomer)[26].

R_f = 0.47 (petroleum ether 40–65/Et_2O/Et_3N, 3:7:0.1);

^{1}H NMR (600 MHz, $CDCl_3$) δ = 7.13 (dd, *J* = 7.4, 0.7 Hz, 1H), 7.06 (ddd, *J* = 7.7, 1.2 Hz, 1H), 6.77 (ddd, *J* = 7.4, 0.9 Hz, 1H), 6.57 (ddd, *J* = 7.8, 0.7 Hz, 1H), 4.00 (dd, *J* = 3.9 Hz, 1H), 3.90 (s, 1H), 3.61 – 3.49 (m, 2H), 3.34 – 3.25 (m, 1H), 2.98 (dd, *J* = 16.0, 3.8 Hz, 1H), 2.92 (ddd, *J* = 12.4, 8.7, 7.3 Hz, 1H), 2.67 (d, *J* = 1.7 Hz, 1H), 2.54 (dd, *J* = 16.0, 4.1 Hz, 1H), 2.45 – 2.34 (m, 2H), 2.30 (ddd, *J* = 8.3, 6.5, 1.8 Hz, 1H), 2.23 (ddd, *J* = 11.0, 6.6, 4.2 Hz, 1H), 2.03 – 1.86 (m, 3H), 1.65 – 1.56 (m, 1H), 1.19 – 1.11 (m, 1H), 0.73 (dd, *J* = 7.4, 7.4 Hz, 3H) ppm;

^{13}C NMR (151 MHz, $CDCl_3$) δ = 212.8, 149.8, 135.6, 128.3, 123.3, 119.5, 109.6, 77.4, 77.2, 77.0, 73.5, 66.2, 52.8, 52.0, 51.4, 50.5, 43.0, 41.5, 40.5, 31.5, 22.4, 12.9 ppm;

HRMS (ESI) *m*/*z*: $[M+H]^+$ Calcd for $C_{19}H_{26}ClN_2O$ 333.1728; Found 333.1732;

---

[26] Determined from the ^{1}H spectrum of the crude product.

IR (thin film) 3363 (w, N–H), 2956 (s), 2924 (s), 2870 (m), 2854 (m), 1706 (s, C=O), 1609 (m, N–H), 1485 (m), 1464 (m), 744 (m, C–Cl) cm^{-1};

$[\boldsymbol{\alpha}]_{\boldsymbol{D}}^{\mathbf{20}}$ +24 (*c* 0.42, $CHCl_3$);

m.p. 93–95 °C[27].

[27] Measured from racemic material, due to the small quantity of enantioenriched material obtained.

5.15 Preparation of (3a*R*,3a[1]*R*,5a*S*,10b*S*)-3a-ethyl-2,3,3a,3a[1],5a,6,11,12-octahydro-1*H*-indolizino[8,1-*cd*]carbazol-4(5*H*)-one (16)

Route A

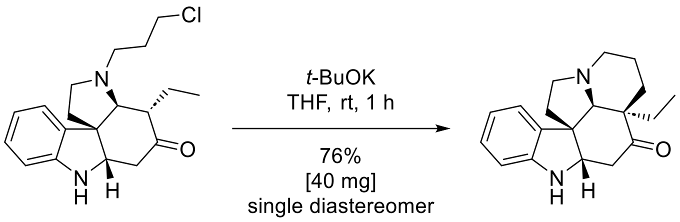

t-BuOK (29 mg, 0.24 mmol) was added to a flask, followed by compound **15** (58 mg, 0.17 mmol) in THF (3.5 mL). The resulting mixture was stirred at room temperature for 1 h. The reaction mixture was then quenched with sat. aq. NH_4Cl. The aqueous layer was extracted with EtOAc. The combined organic extracts were dried over brine, $MgSO_4$, filtered and concentrated under reduced pressure. The crude product was purified by flash chromatography (petroleum ether 40–65/Et_2O/Et_3N, 6:4:0.1) to afford compound **16** as a white solid (40 mg, 0.013 mmol, 76%, single diastereomer)[28].

Route B

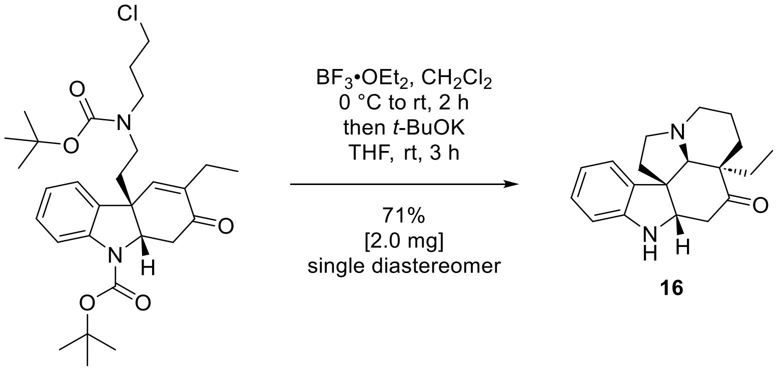

Compound **14** (5.0 mg, 9.4 µmol) in CH_2Cl_2 (0.50 mL) was added to a flask. The solution was cooled to 0 °C and BF_3•OEt_2 (10 µL, 81 µmol) was added dropwise. The resulting mixture was stirred at room temperature for 2 h. The reaction mixture was then concentrated under reduced pressure. The residue was dissolved in THF (0.20 mL) and *t*-BuOK (13 mg, 0.12 mmol) was added. The resulting mixture was stirred at room temperature for 3 h. The reaction mixture was then quenched with sat. aq. NH_4Cl. The aqueous layer was extracted with EtOAc. The

[28] Determined from the ^{1}H spectrum of the crude product.

combined organic extracts were dried over brine, $MgSO_4$, filtered and concentrated under reduced pressure. The crude product was purified by flash chromatography (petroleum ether 40–65/Et_2O/Et_3N, 6:4:0.1) to afford compound **16** as a white solid (2.0 mg, 6.7 µmol, 71%, single diastereomer)[29].

R_f = 0.28 (petroleum ether 40–65/Et_2O/Et_3N, 6:4:0.1);

^{1}H NMR (600 MHz, $CDCl_3$) δ = 7.15 (dd, *J* = 7.4, 0.7 Hz, 1H), 7.05 (ddd, *J* = 7.6, 7.6, 1.3 Hz, 1H), 6.77 (dd, *J* = 7.4, 7.4, 1.0 Hz, 1H), 6.58 (d, *J* = 7.8 Hz, 1H), 4.00 (dd, *J* = 4.4, 4.4 Hz, 1H), 3.94 (s, 1H), 3.13 (td, *J* = 8.7, 2.1 Hz, 1H), 3.06 (dd, *J* = 16.6, 4.8 Hz, 1H), 3.03 – 2.98 (m, 1H), 2.56 (dd, *J* = 16.7, 3.9 Hz, 1H), 2.32 (app. q, *J* = 9.0 Hz, 1H), 2.27 (s, 1H)[30], 2.31 – 2.20 (m, 1H), 2.02 (ddd, *J* = 13.4, 8.9, 8.9 Hz, 1H), 1.92 (ddd, *J* = 11.5, 11.1, 3.2 Hz, 1H), 1.55 – 1.49 (m, 1H), 1.49 – 1.38 (m, 2H), 1.36 – 1.27 (m, 1H), 0.94 (td, *J* = 13.3, 4.9 Hz, 1H), 0.48 (dd, *J* = 7.5, 7.5 Hz, 3H) ppm;

^{13}C NMR (151 MHz, $CDCl_3$) δ = 213.5, 150.0, 135.9, 128.1, 123.8, 119.4, 109.7, 76.2, 67.2, 53.8, 53.1, 52.2, 51.0, 42.2, 42.1, 30.9, 27.8, 22.8, 7.8 ppm;

HRMS (ESI) *m*/*z*: $[M+H]^+$ $C_{19}H_{25}N_2O$ 297.1961; Found 297.1948;

IR (thin film) 3361 (w), 2953 (m), 2922 (s), 2868 (m), 2853 (m), 1734 (w), 1704 (w), 1486 (m), 1377 (m) cm^{-1};

$[\boldsymbol{\alpha}]_{\boldsymbol{D}}^{\mathbf{20}}$ −2.8 (*c* 0.36, $CHCl_3$);

m.p. 139–141 °C[31].

---

[29] Determined from the ^{1}H spectrum of the crude product.
[30] Overlapping signals therefore not possible to accurately integrate, the integrals shown are corrected.
[31] Measured from racemic material, due to the small quantity of enantioenriched material obtained.

5.16 Preparation of (−)-Aspidospermidine (1)

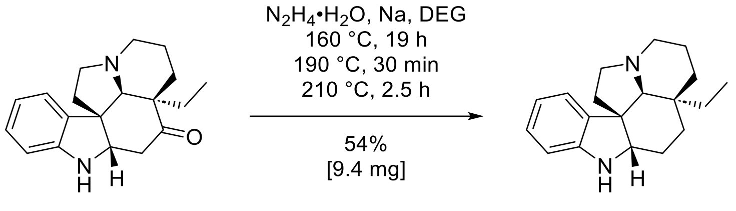

Compound **16** (18 mg, 0.061 mmol) was added to a flask, followed by diethylene glycol (DEG) (0.61 mL) and Na (260 mg, 11 mmol). Hydrazine monohydrate (0.75 mL, 15 mmol) was then added dropwise. The resulting mixture was stirred at 160 °C for 19 h, 190 °C for 30 min and 210 °C for 2.5 h. The reaction mixture was allowed to cool to room temperature and then diluted with H_2O. The aqueous layer was extracted with CH_2Cl_2. The combined organic extracts were dried over brine, $MgSO_4$, filtered and concentrated under reduced pressure. The crude product was purified by repeated flash chromatography (petroleum ether 40–65/Et_2O/Et_3N, 6:4:0.1; PhMe/EtOAc/Et_3N, 9:1:0.1) to afford (−)-aspidospermidine (**1**) as a white solid (9.4 mg, 0.033 mmol, 54%).

The obtained spectroscopic data matched literature values.[17]

R_f = 0.31 (petroleum ether 40–65/Et_2O/Et_3N, 6:4:0.1), 0.38 (PhMe/EtOAc/Et_3N, 9:1:0.1);

^{1}H NMR (600 MHz, $CDCl_3$) δ = 7.08 (dd, *J* = 7.4, 1.3 Hz, 1H), 7.01 (ddd, *J* = 7.6, 7.6, 1.3 Hz, 1H), 6.73 (ddd, *J* = 7.4, 7.4, 1.0 Hz, 1H), 6.64 (d, *J* = 7.7 Hz, 1H), 3.51 (dd, *J* = 11.1, 6.2 Hz, 1H), 3.16 – 3.09 (m, 1H), 3.08 – 3.02 (m, 1H), 2.34 – 2.23 (m, 2H), 2.22 (s, 1H), 2.00 – 1.90 (m, 2H), 1.79 – 1.68 (m, 1H), 1.67 – 1.59 (m, 2H), 1.55 – 1.43 (m, 3H), 1.39 (dddd, *J* = 14.4, 13.2, 11.1, 3.6 Hz, 1H), 1.11 (td, *J* = 13.6, 13.6, 4.7 Hz, 1H), 1.06 (ddd, *J* = 13.5, 3.7 Hz, 1H), 0.92 – 0.82 (m, 1H), 0.64 (dd, *J* = 7.5, 7.5 Hz, 3H) ppm;

^{13}C NMR (151 MHz, $CDCl_3$) δ = 149.6, 135.9, 127.2, 123.0, 119.1, 110.5, 71.4, 65.8, 54.0, 53.5, 53.2, 39.0, 35.8, 34.6, 30.1, 28.3, 23.2, 21.9, 7.0 ppm;

HRMS (ESI) *m/z*: $[M+H]^+$ Calcd for $C_{19}H_{27}N_2$ 283.2169; Found 283.2186;

IR (thin film) 3364 (w), 2929 (s), 2860 (m), 2779 (m), 2722 (w), 1607 (m), 1481 (m), 1462 (s), 741 (s) cm^{-1};

$[\boldsymbol{\alpha}]_{\boldsymbol{D}}^{\mathbf{20}}$ −17 (*c* 0.065, $CHCl_3$), lit.[17] $[\boldsymbol{\alpha}]_{\boldsymbol{D}}^{\mathbf{20}}$ +20.5 (*c* 0.6, $CHCl_3$)[32];

m.p. 104–106 °C, lit.[18] 104–106 °C[33].

[32] Optical rotation from enantiopure (+)-aspidospermidine (**1**).
[33] Measured from racemic material, due to the small quantity of enantioenriched material obtained.

Table S4 Comparison of Synthetic Aspidospermidine (1) ^{1}H NMR Data

Entry	Marino's Synthetic (+)-**1** [ppm] (500 MHz)[17]	Our Synthetic (−)-**1** [ppm] (600 MHz)	Δδ (δ_{ours} – $\delta_{Marino's}$) [ppm][34]
1	7.09 (d, *J* = 7.5 Hz, 1H)	7.08 (dd, *J* = 7.4, 1.3 Hz, 1H)	−0.01
2	7.03 (t, *J* = 7.5 Hz, 1H)	7.01 (ddd, *J* = 7.6, 7.6, 1.3 Hz, 1H)	−0.02
3	6.74 (t, *J*=7.2 Hz, 1H)	6.73 (ddd, *J* = 7.4, 7.4, 1.0 Hz, 1H)	−0.01
4	6.65 (d, *J* = 7.5 Hz, 1H)	6.64 (d, *J* = 7.7 Hz, 1H)	−0.01
5	3.52 (dd, *J* = 11, 7.0 Hz, 1H)	3.51 (dd, *J* = 11.1, 6.2 Hz, 1H)	−0.01
6	3.15 – 3.12 (m, 1 H)	3.16 – 3.09 (m, 1H)	—
7	3.08 (br d *J* = 10.5 Hz, 1H)	3.08 – 3.02 (m, 1H)	—
8	2.35 – 2.22 (m, 2H)	2.34 – 2.23 (m, 2H)	—
9	2.24 (s, 1H)	2.22 (s, 1H)	−0.02
10	1.99 – 1.92 (m, 2H)	2.00 – 1.90 (m, 2H)	—
11	1.81 – 1.71 (m, 1H)	1.79 – 1.68 (m, 1H)	—
12	1.68 – 1.62 (m, 2H)	1.67 – 1.59 (m, 2H)	—
13	1.54 – 1.47 (m, 2H)	1.55 – 1.43 (m, 3H)	—
14	1.42 – 1.36 (m, 1H)	1.39 (dddd, *J* = 14.4, 13.2, 11.1, 3.6 Hz, 1H)	—
15	1.26 (m, 1 H)	1.11 (td, *J* = 13.6, 13.6, 4.7 Hz, 1H)	—
16	1.16 – 1.05 (m, 1H)	1.06 (ddd, *J* = 13.5, 3.7 Hz, 1H)	—
17	0.92 – 0.84 (m, 1H)	0.92 – 0.82 (m, 1H)	—
18	0.64 (t, *J* = 6.6 Hz, 3H)	0.64 (dd, *J* = 7.5, 7.5 Hz, 3H)	0

[34] A systematic shift is present. Our spectra were referenced using the residual solvent peak of $CDCl_3$ at 7.26 ppm.

Table S5 Comparison of Natural and Synthetic Aspidospermidine (1) ^{13}C NMR Data

Entry	He's Natural (+)-**1** [ppm] (125 MHz)[19]	Marino's Synthetic (+)-**1** [ppm] (125 MHz)[17]	Our Synthetic (−)-**1** [ppm] (151 MHz)	Δδ (δ_{ours} − $\delta_{natural}$)[35] [ppm]	Δδ (δ_{ours} − $\delta_{Marino's}$) [ppm]
1	149.3	149.6	149.6	0.3	0
2	135.6	135.9	135.9	0.3	0
3	127.0	127.3	127.2	0.2	−0.1
4	122.7	123.0	123.0	0.3	0
5	118.8	119.2	119.1	0.3	−0.1
6	110.1	110.6	110.5	0.4	−0.1
7	71.1	71.5	71.4	0.3	−0.1
8	65.4	65.9	65.8	0.4	−0.1
9	53.7	54.1	54.0	0.3	−0.1
10	52.9	53.9	53.5	0.6	−0.4
11	52.9	53.2	53.2	0.3	0
12	38.7	39.0	39.0	0.3	0
13	35.5	35.8	35.8	0.3	0
14	34.3	34.7	34.6	0.3	−0.1
15	29.8	30.2	30.1	0.3	−0.1
16	28.1	28.3	28.3	0.2	0
17	23.0	23.2	23.2	0.2	0
18	21.6	22.0	21.9	0.3	−0.1
19	6.6	7.0	7.0	0.4	0

[35] A systematic shift is present. Our spectra were referenced using the solvent peak of $CDCl_3$ at 77.16 ppm.

6 NMR Spectra

6.1 ^{1}H NMR Spectrum of Compound 7 (600 MHz, $CDCl_3$)

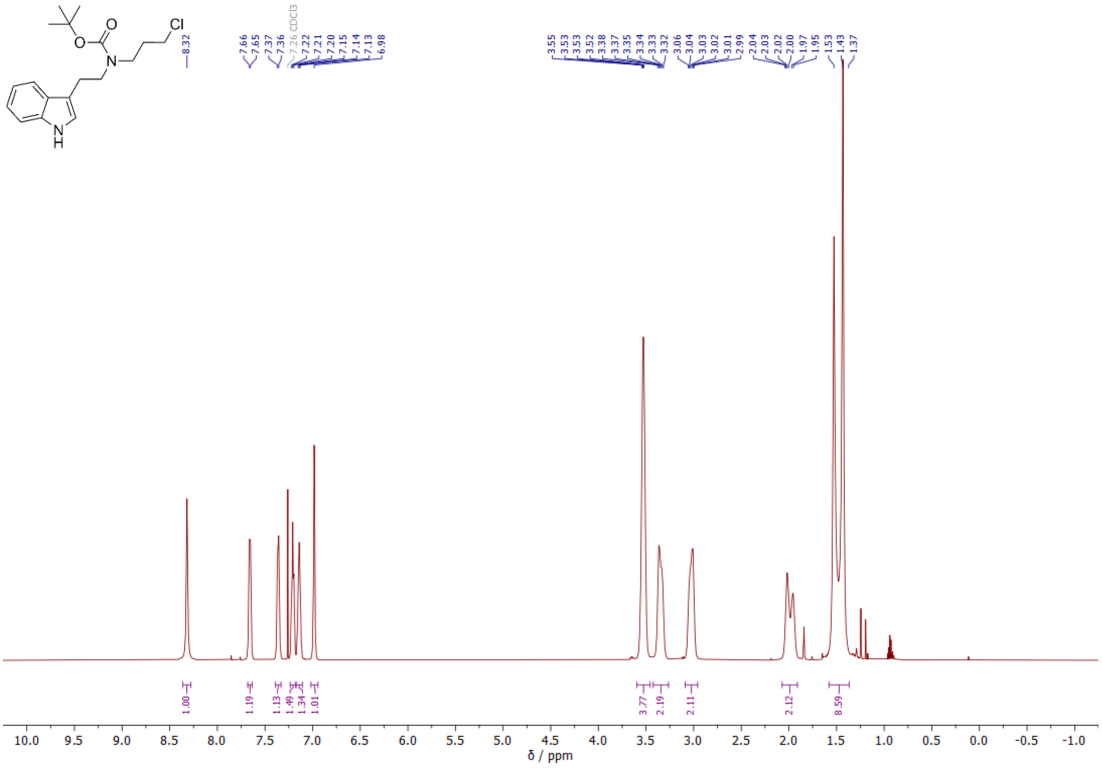

6.2 ^{13}C NMR Spectrum of Compound 7 (151 MHz, $CDCl_3$)

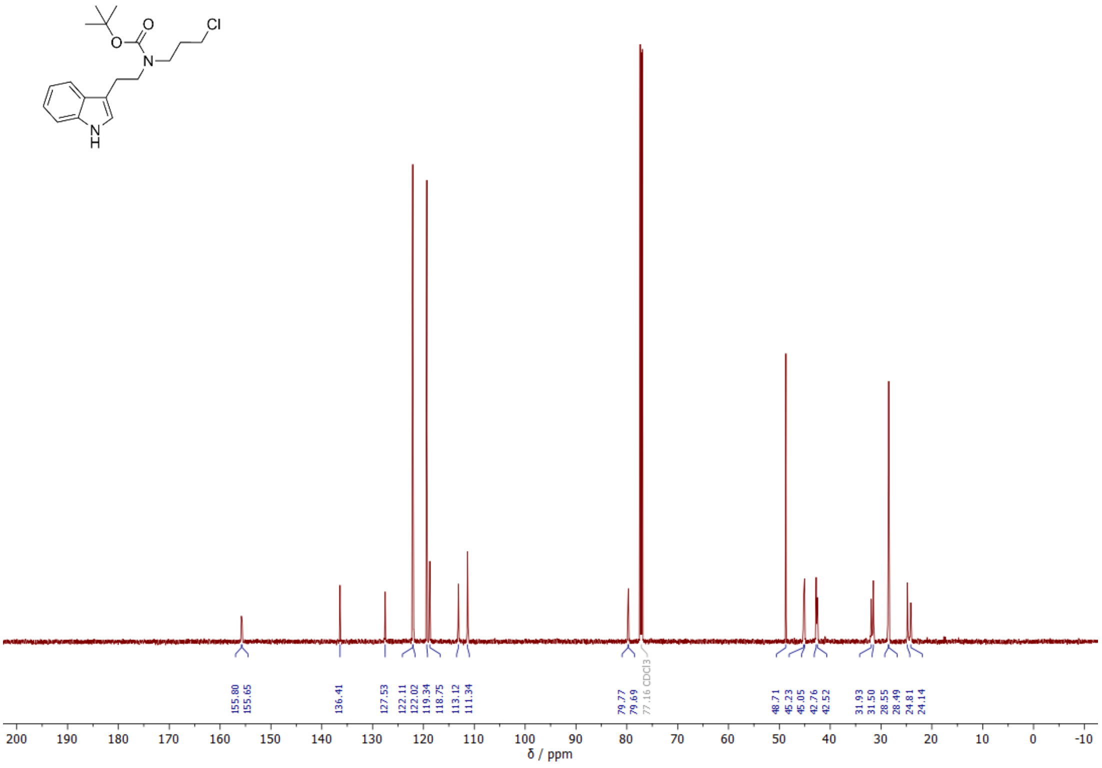

6.3 ^{1}H NMR Spectrum of Compound 9b (600 MHz, $CDCl_3$)

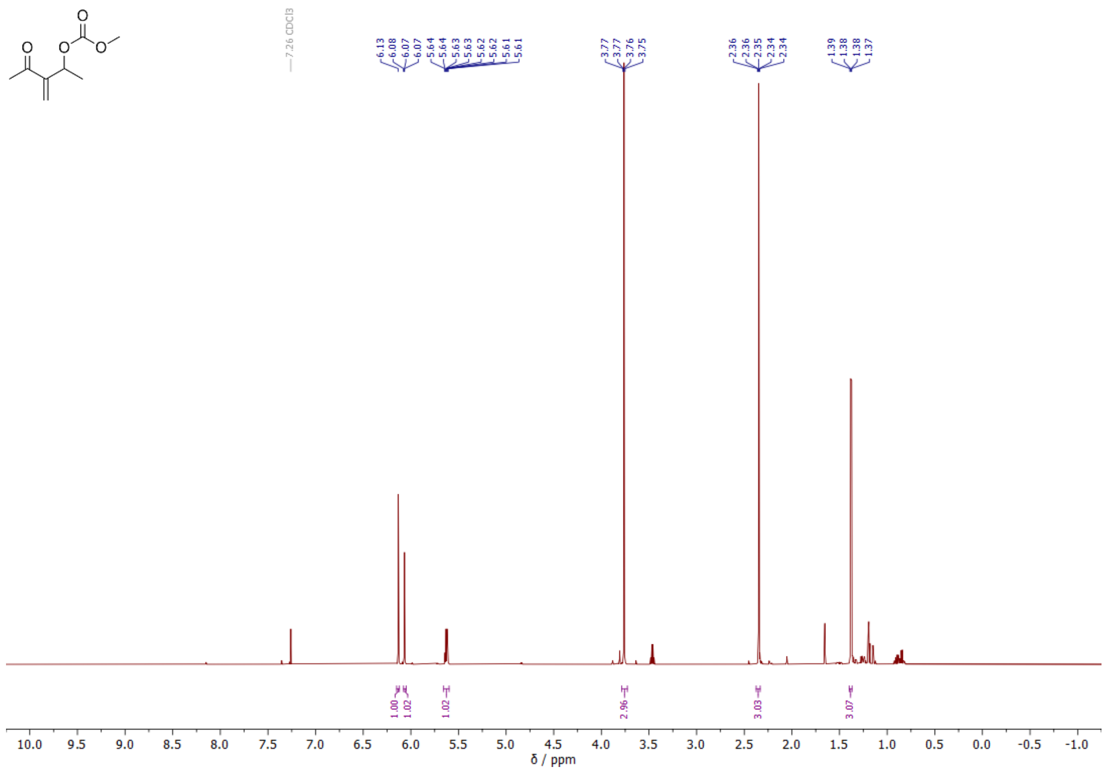

6.4 ^{13}C NMR Spectrum of Compound 9b (151 MHz, $CDCl_3$)

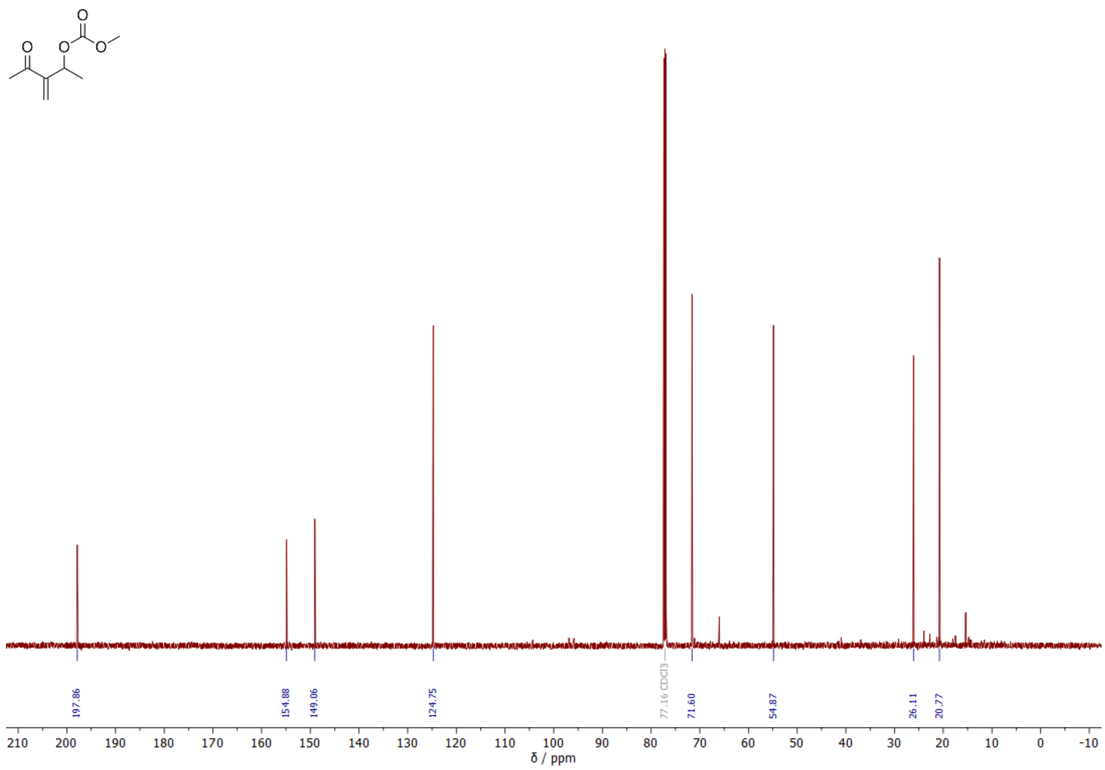

6.5 ^{1}H NMR Spectrum of Compound 9c (400 MHz, $CDCl_3$)

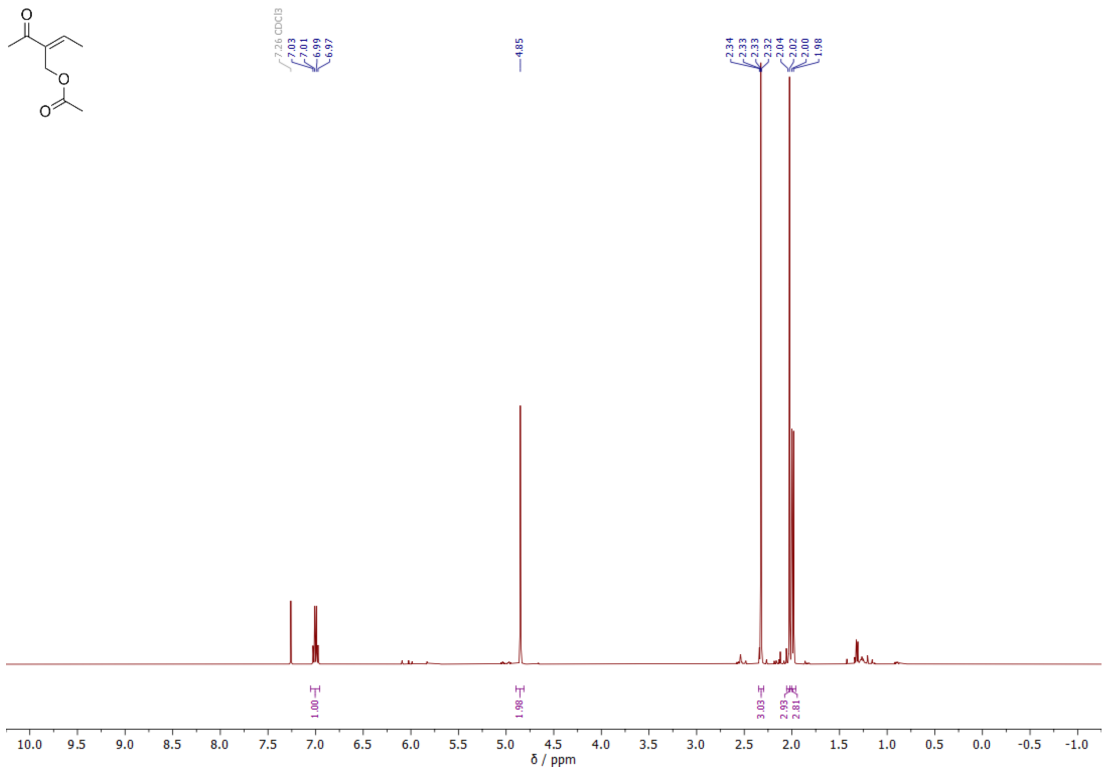

6.6 ^{13}C NMR Spectrum of Compound 9c (101 MHz, $CDCl_3$)

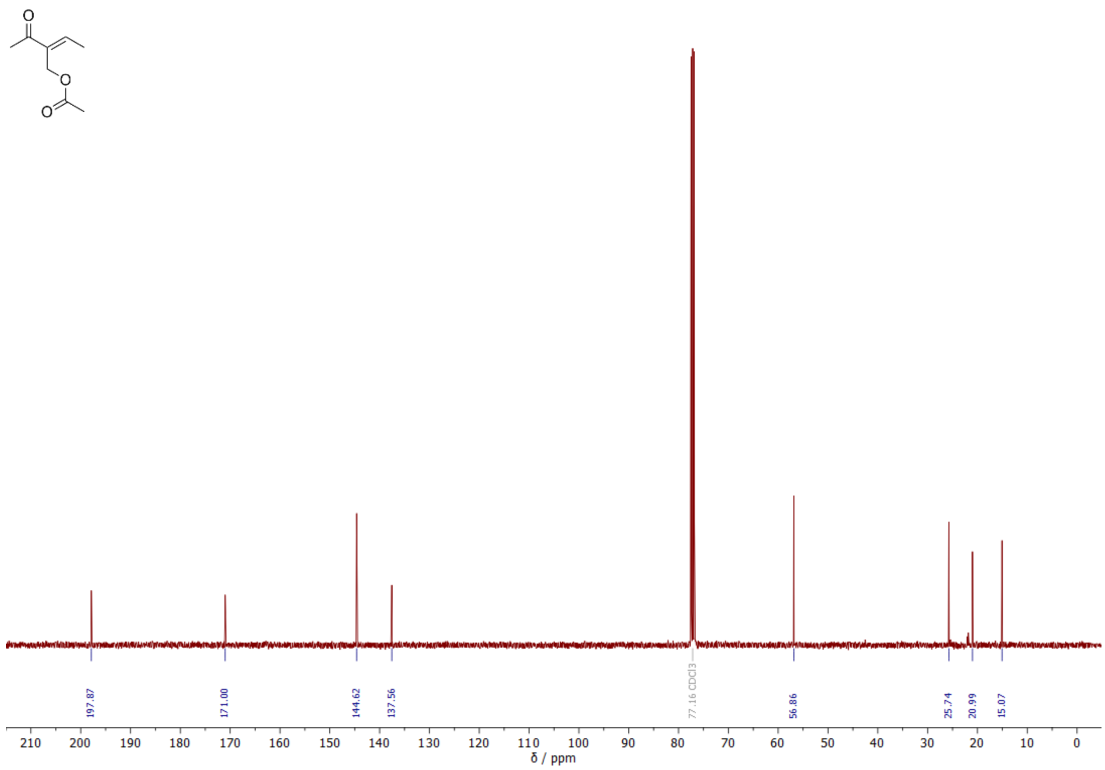

6.7 ^{1}H NMR Spectrum of Compound 10 (600 MHz, $CDCl_3$)

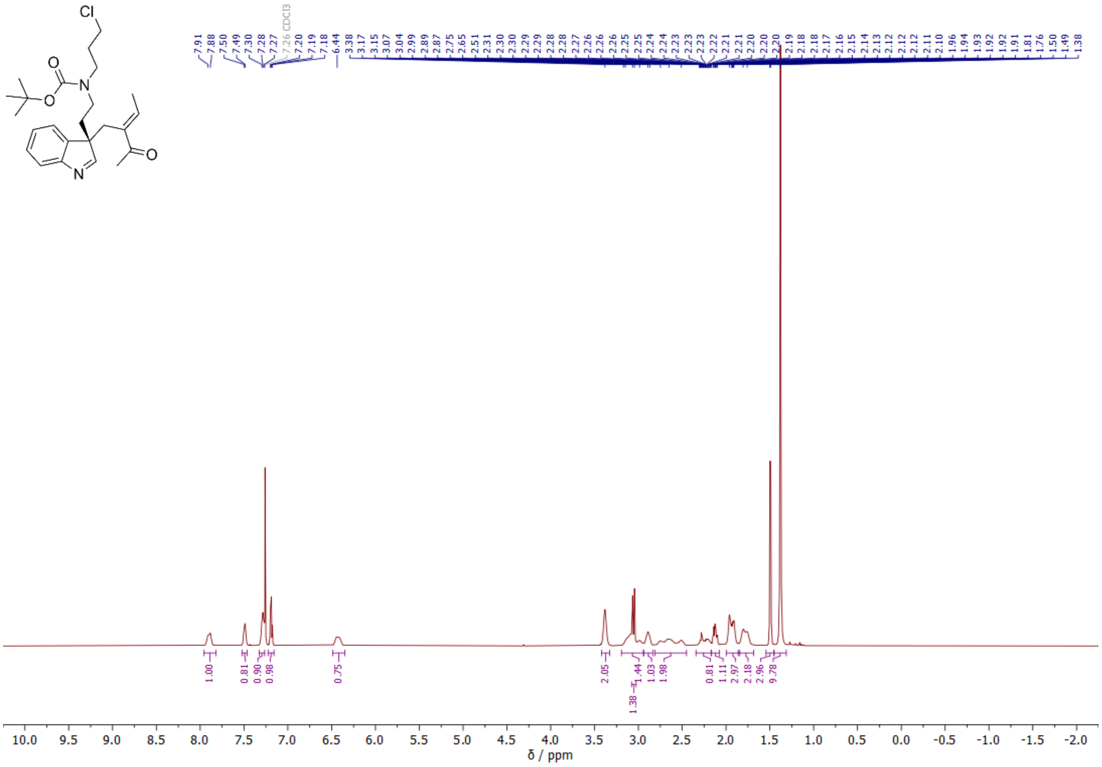

6.8 ^{13}C NMR Spectrum of Compound 10 (151 MHz, $CDCl_3$)

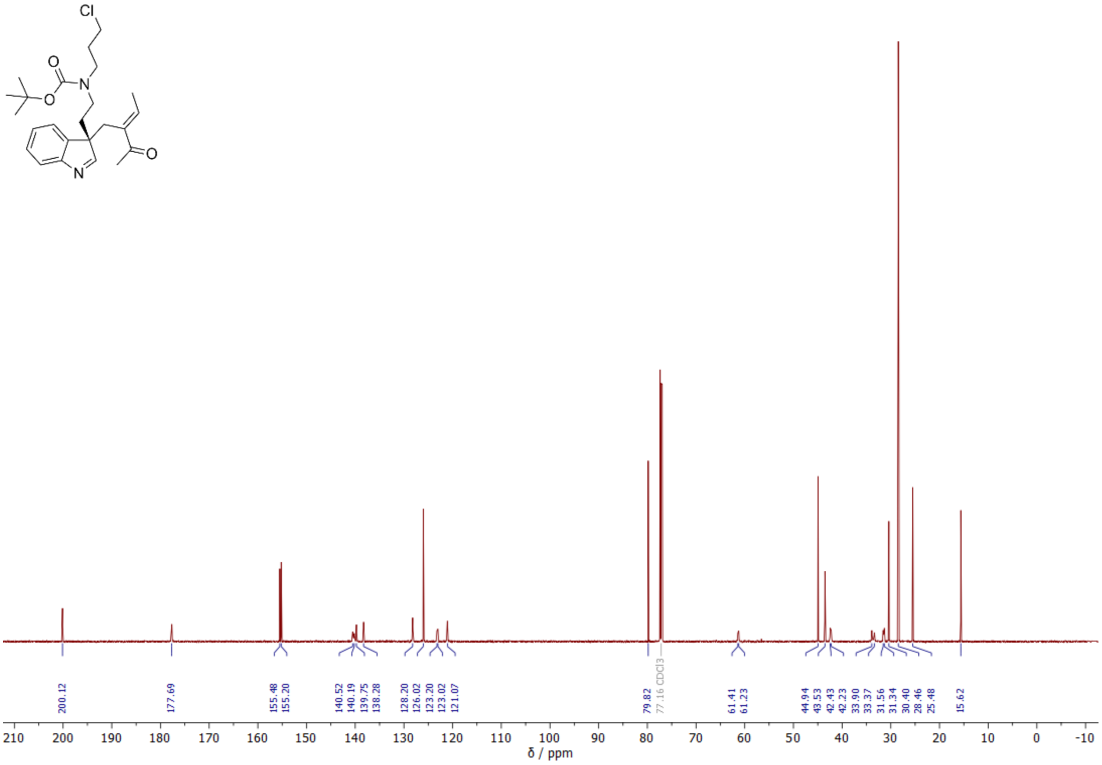

6.9 ^{1}H NMR Spectrum of Compound 11 (600 MHz, $CDCl_3$)

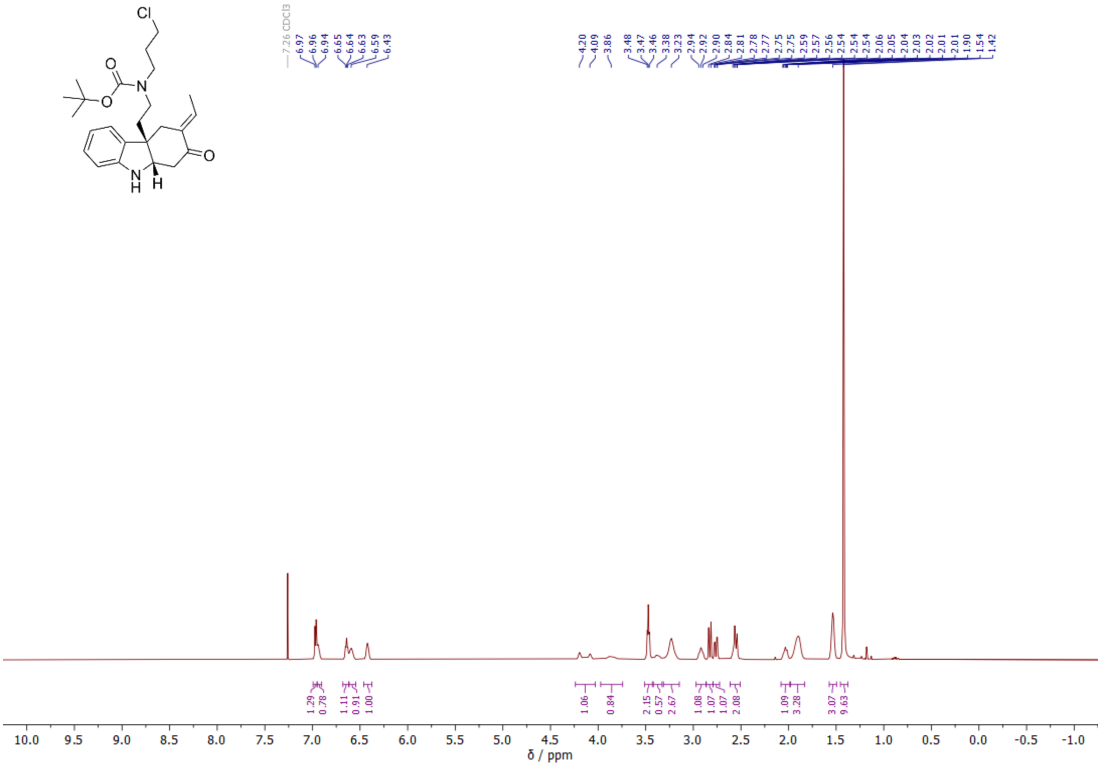

6.10 ^{13}C NMR Spectrum of Compound 11 (151 MHz, $CDCl_3$)

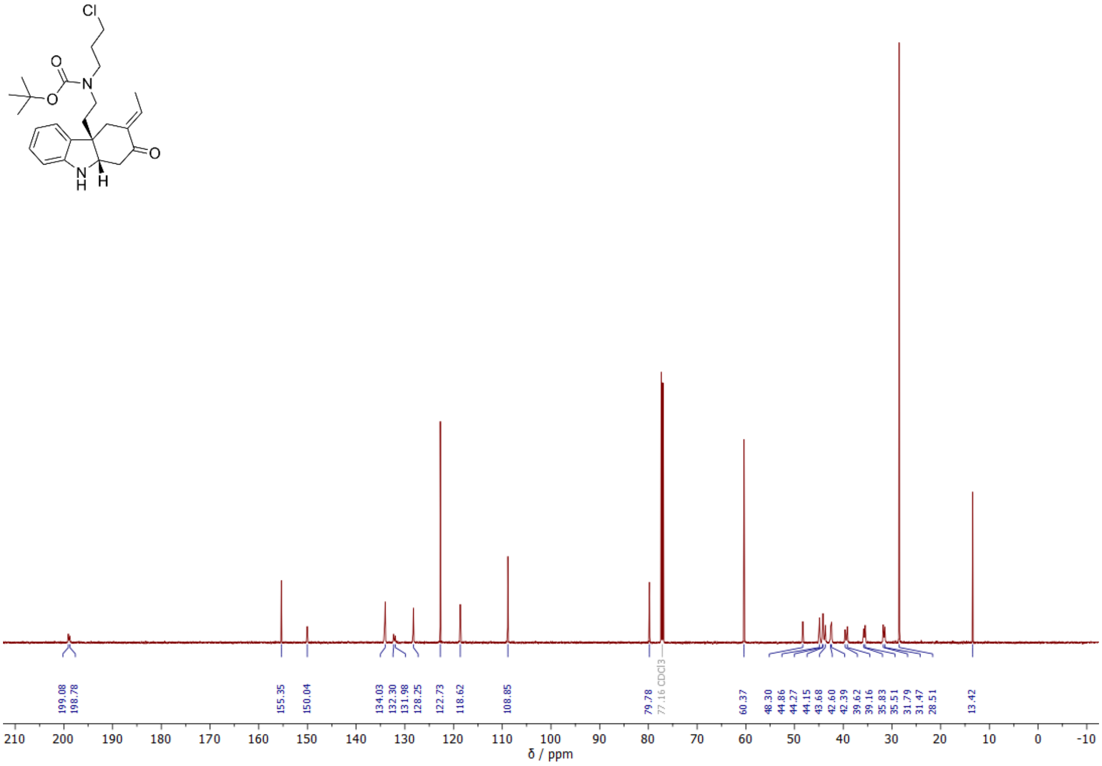

6.11 ^{1}H NMR Spectrum of Compound S2 (400 MHz, $CDCl_3$, 50 °C)

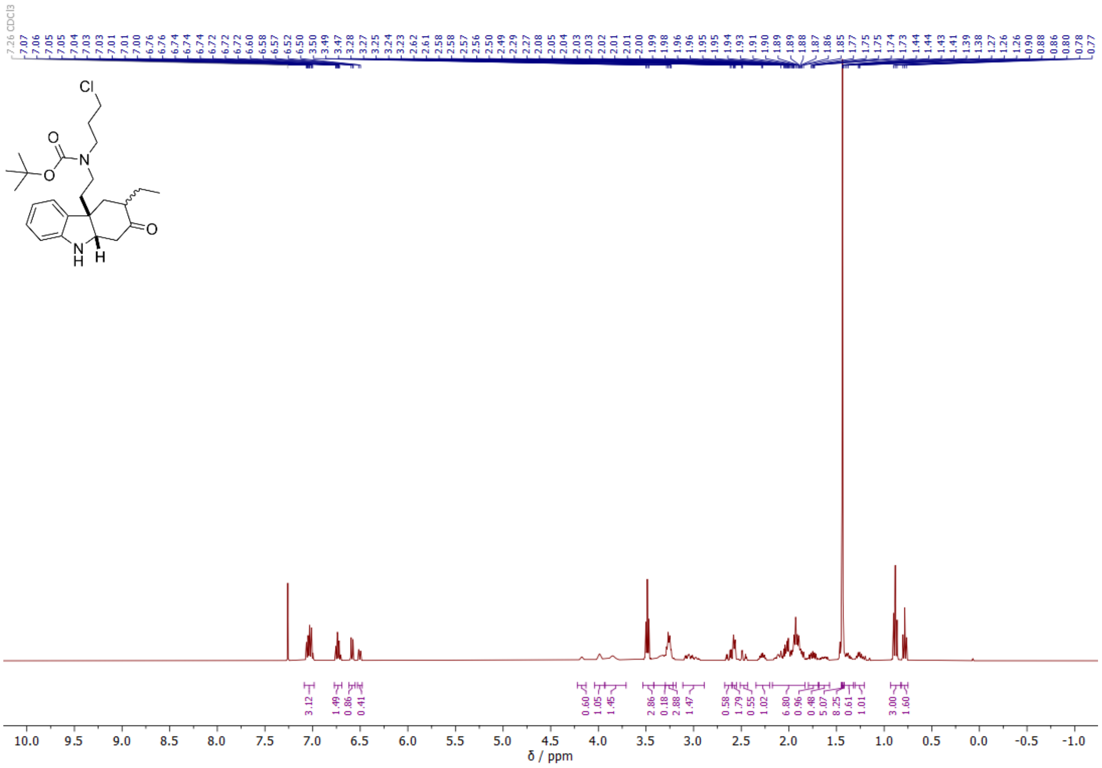

6.12 ^{13}C NMR Spectrum of Compound S2 (101 MHz, $CDCl_3$, 50 °C)

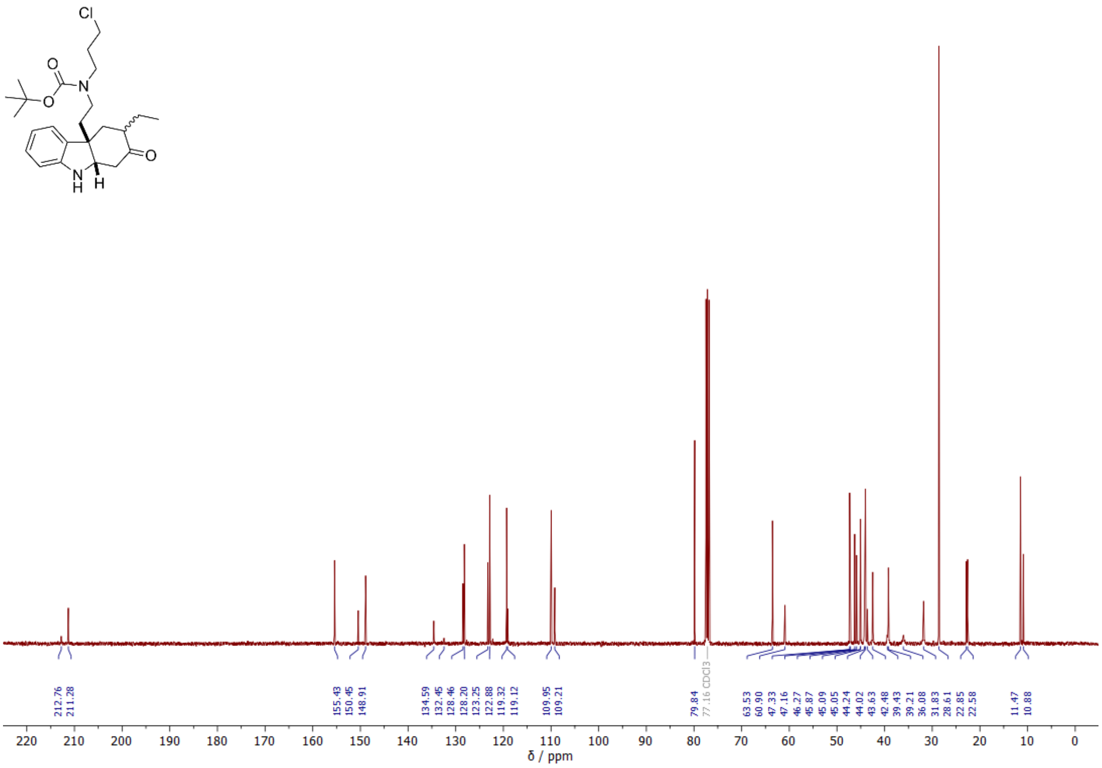

6.13 ^{1}H-^{13}C HSQC Spectrum of Compound S2 ($CDCl_3$, 50 °C)

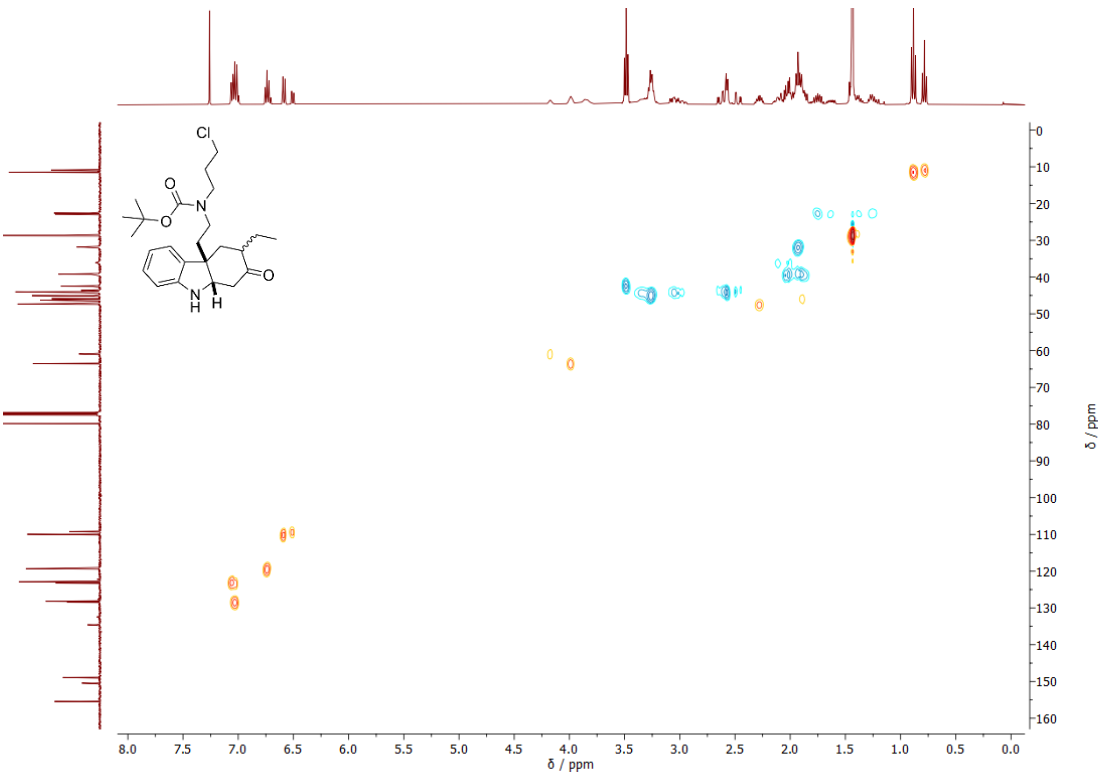

6.14 ^{1}H-^{13}C HMBC Spectrum of Compound S2 ($CDCl_3$, 50 °C)

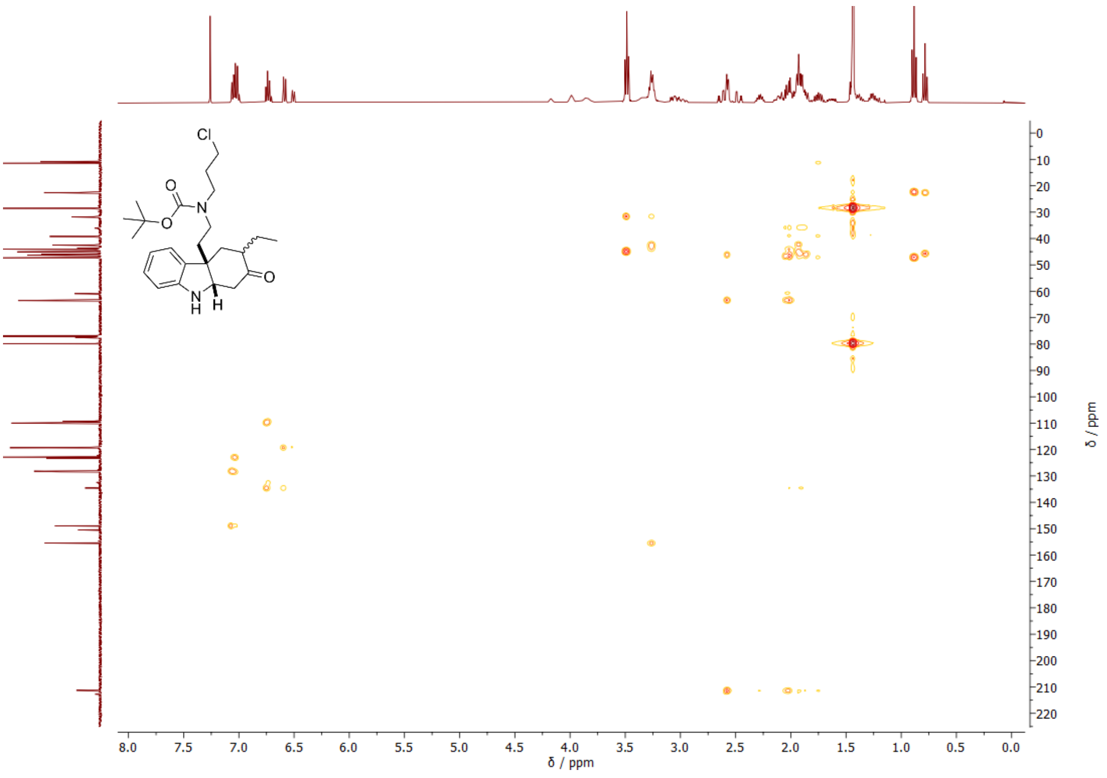

6.15 ^{1}H NMR Spectrum of Compound S3 (400 MHz, $CDCl_3$, 50 °C)[36]

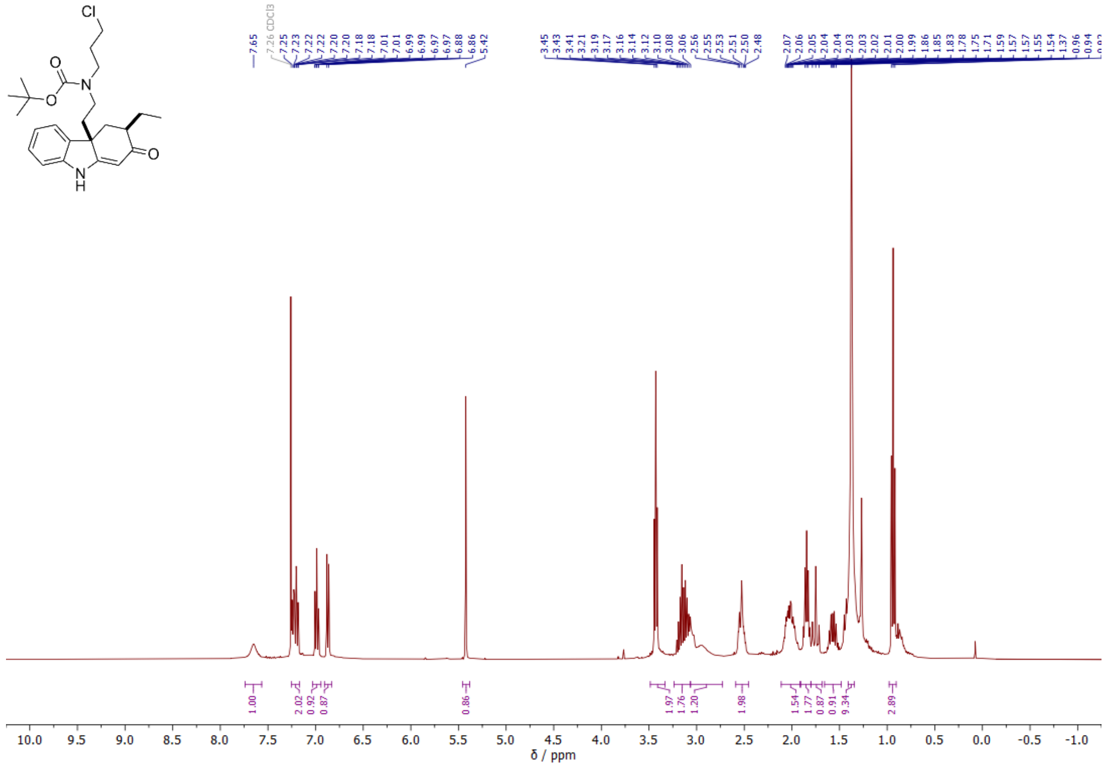

6.16 ^{13}C NMR Spectrum of Compound S3 (101 MHz, $CDCl_3$, 50 °C)

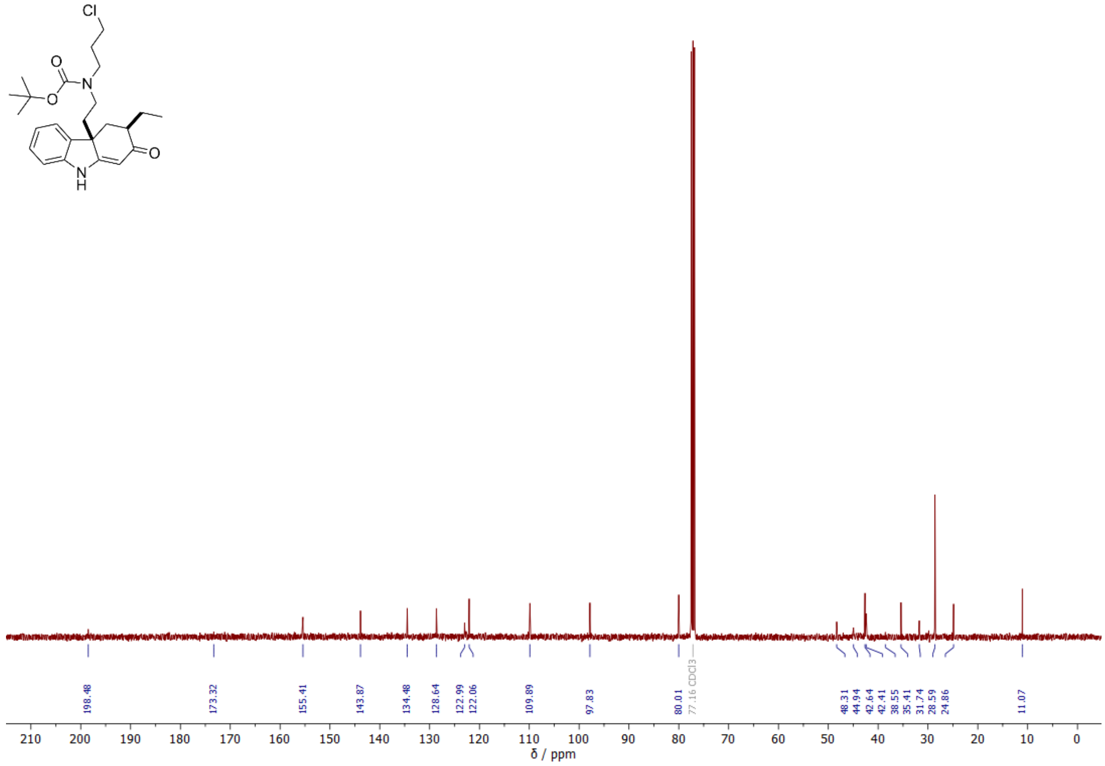

[36] It was not possible to differentiate the diastereomers from the NOESY NMR spectra.

6.17 ^{1}H-^{13}C HSQC Spectrum of Compound S3 ($CDCl_3$, 50 °C)

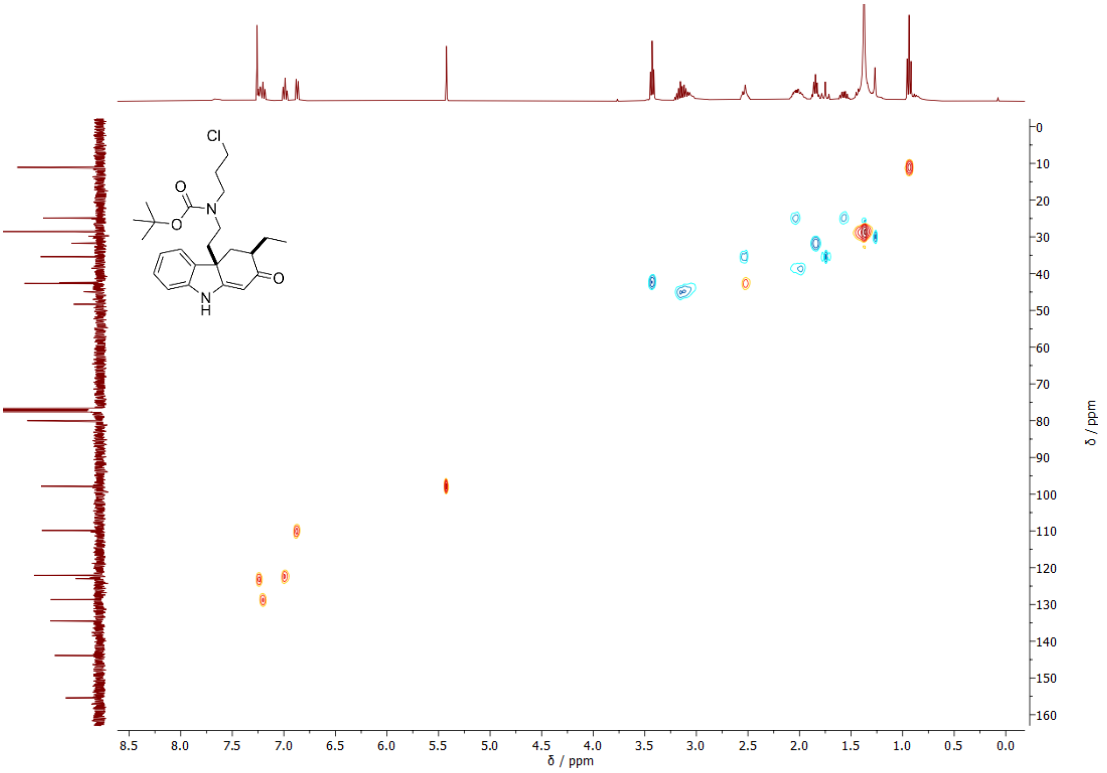

6.18 ^{1}H-^{13}C HMBC NMR Spectrum of Compound S3 ($CDCl_3$, 50 °C)

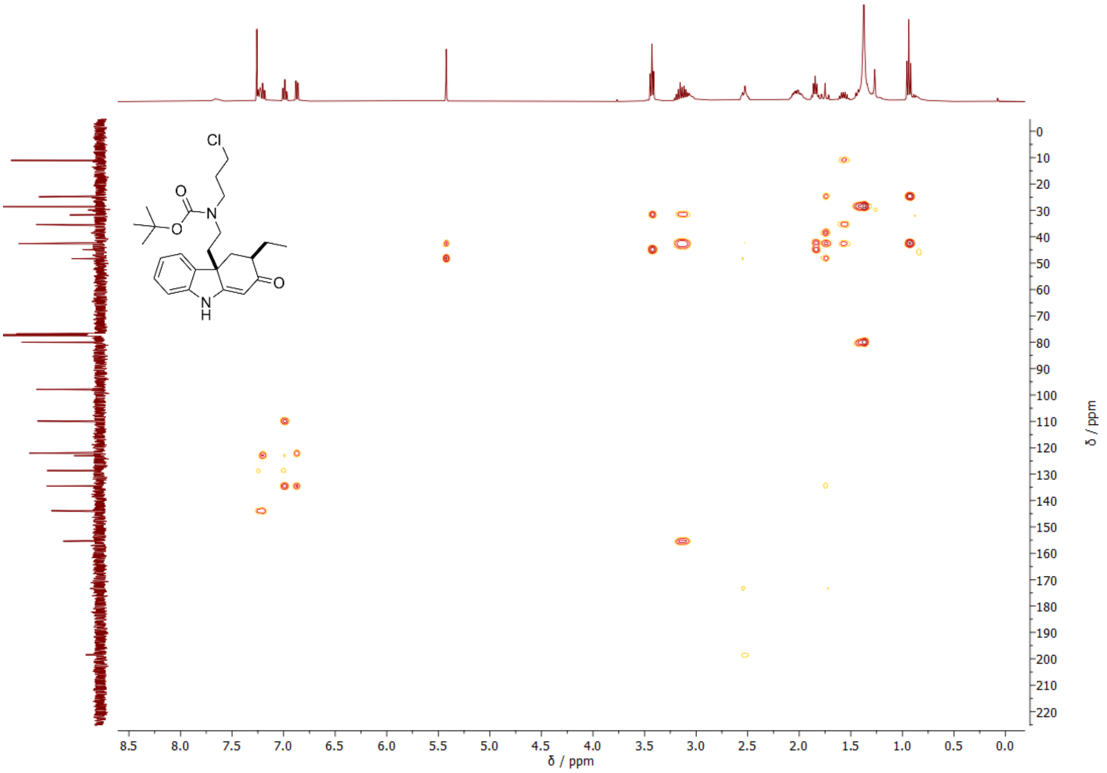

6.19 ^{1}H NMR Spectrum of Compound 12 (400 MHz, $CDCl_3$, 50 °C)

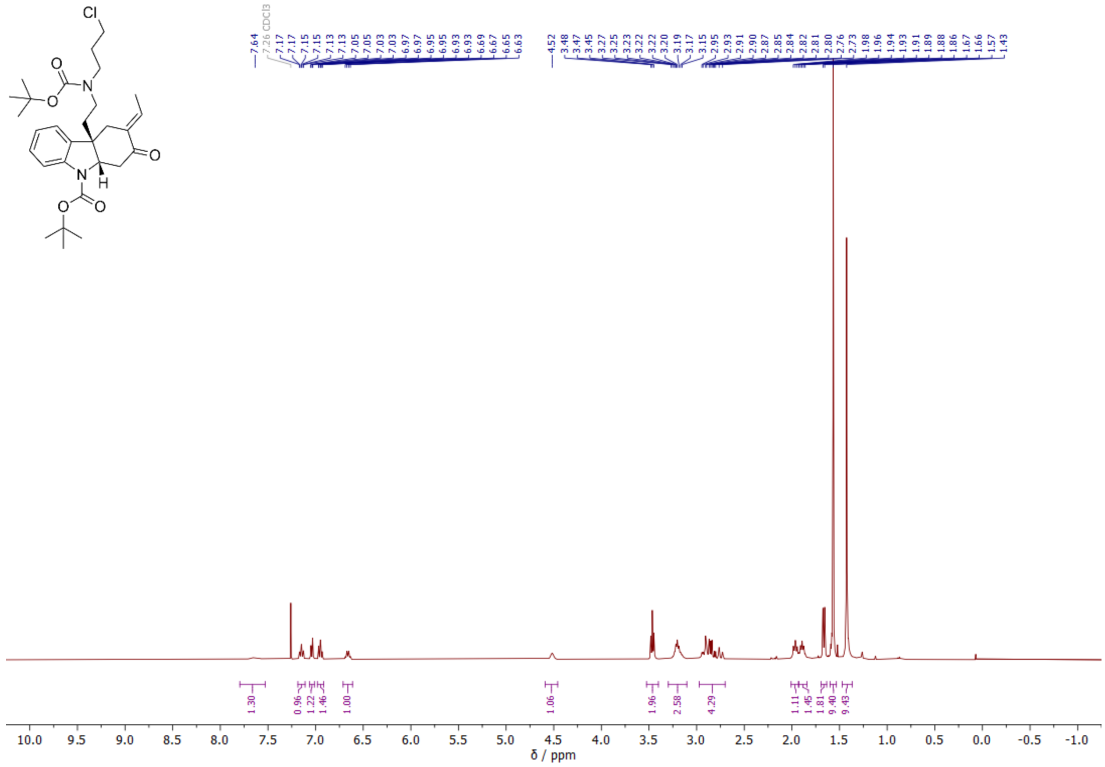

6.20 ^{13}C NMR Spectrum of Compound 12 (101 MHz, $CDCl_3$, 50 °C)

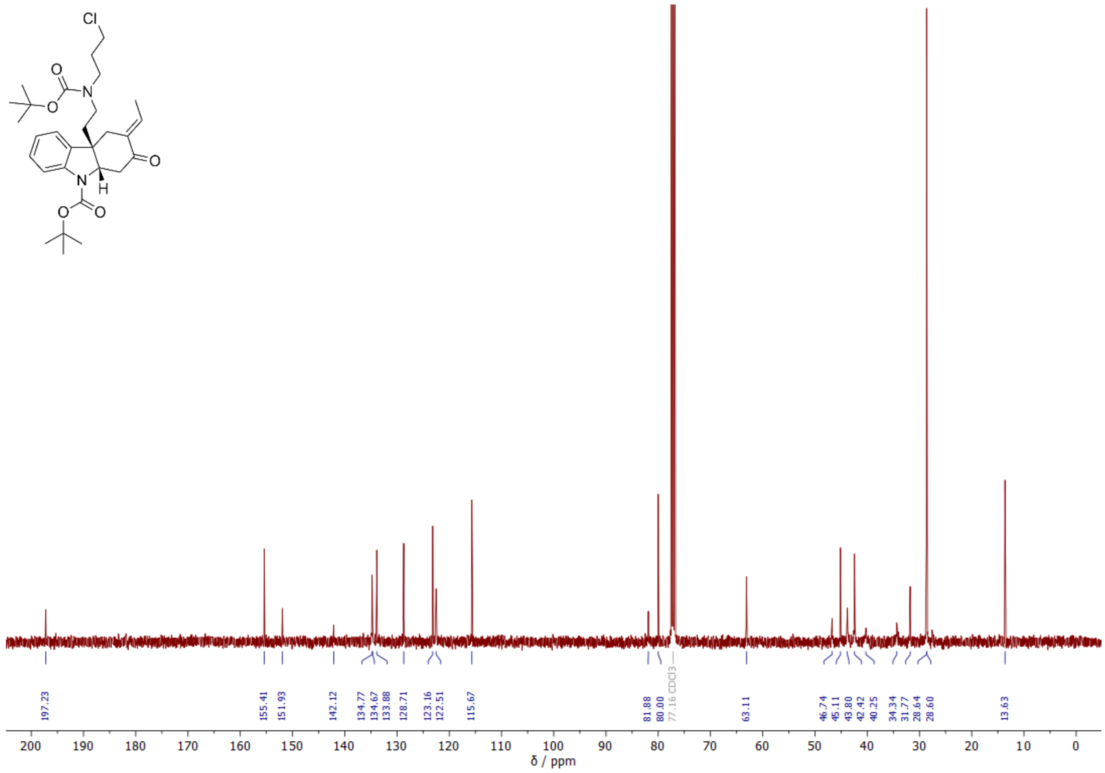

6.21 ^{1}H-^{13}C HSQC Spectrum of Compound 12 (CDCl$_3$, 50 °C)

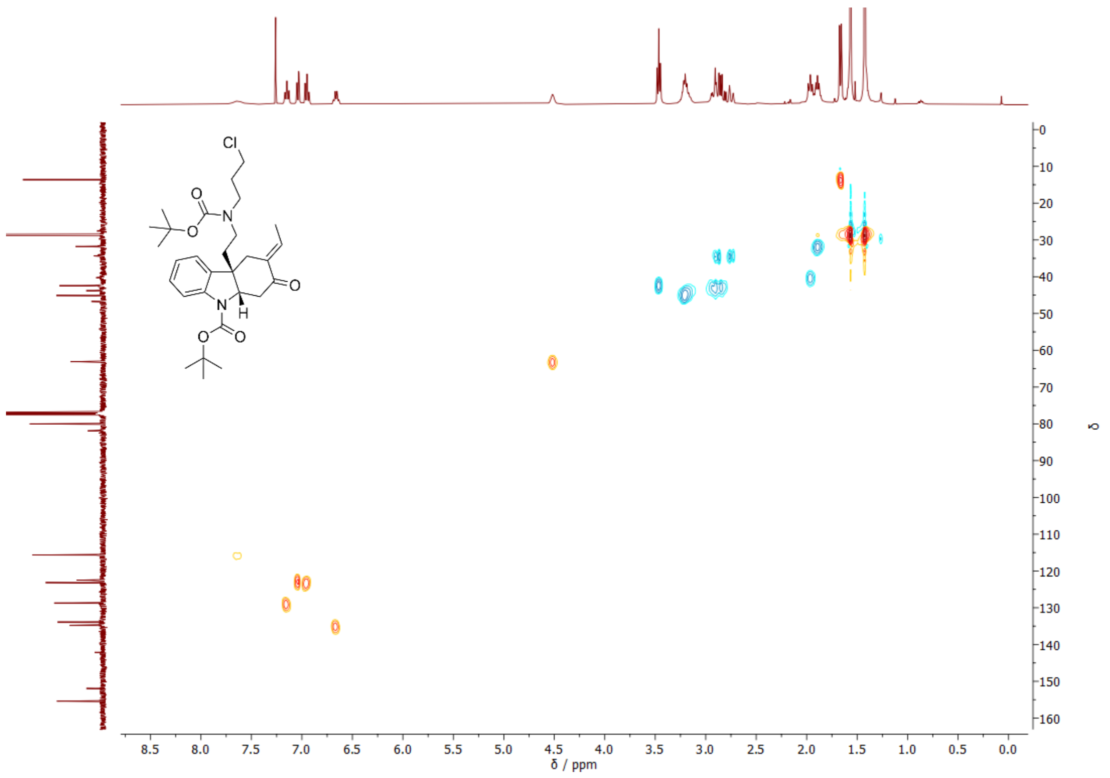

6.22 ^{1}H-^{13}C HMBC Spectrum of Compound 12 (CDCl$_3$, 50 °C)

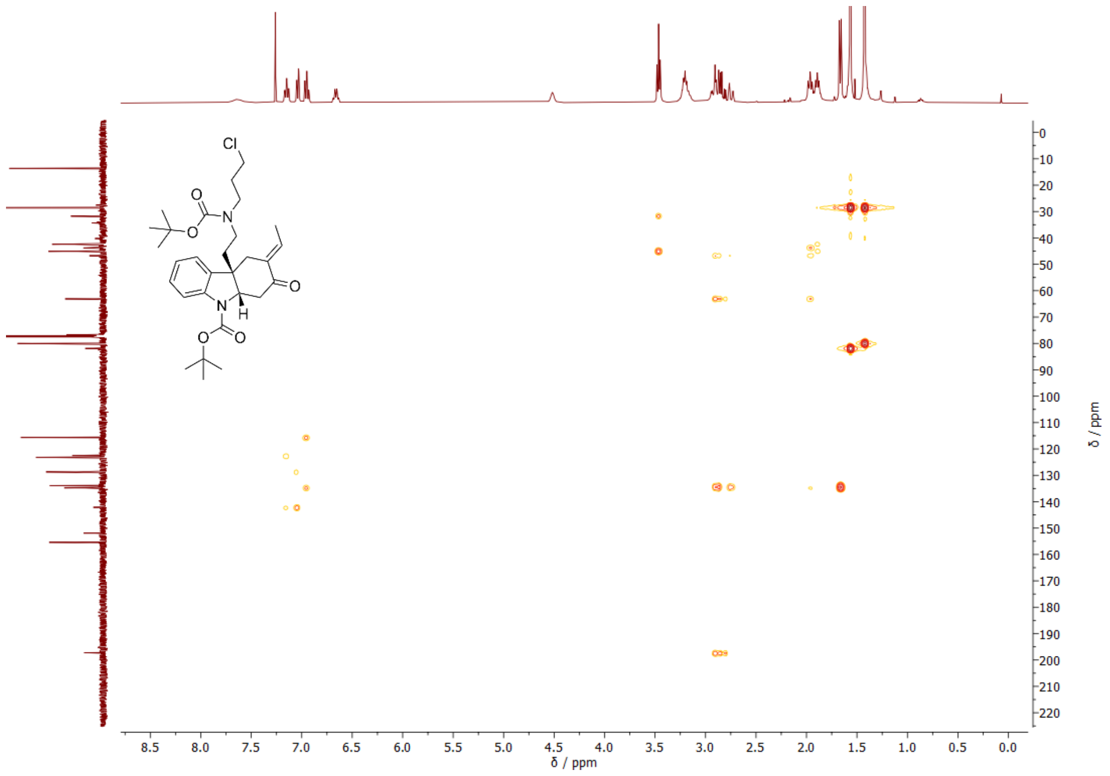

6.23 ^{1}H NMR Spectrum of Compound 13 (400 MHz, $CDCl_3$, 50 °C)

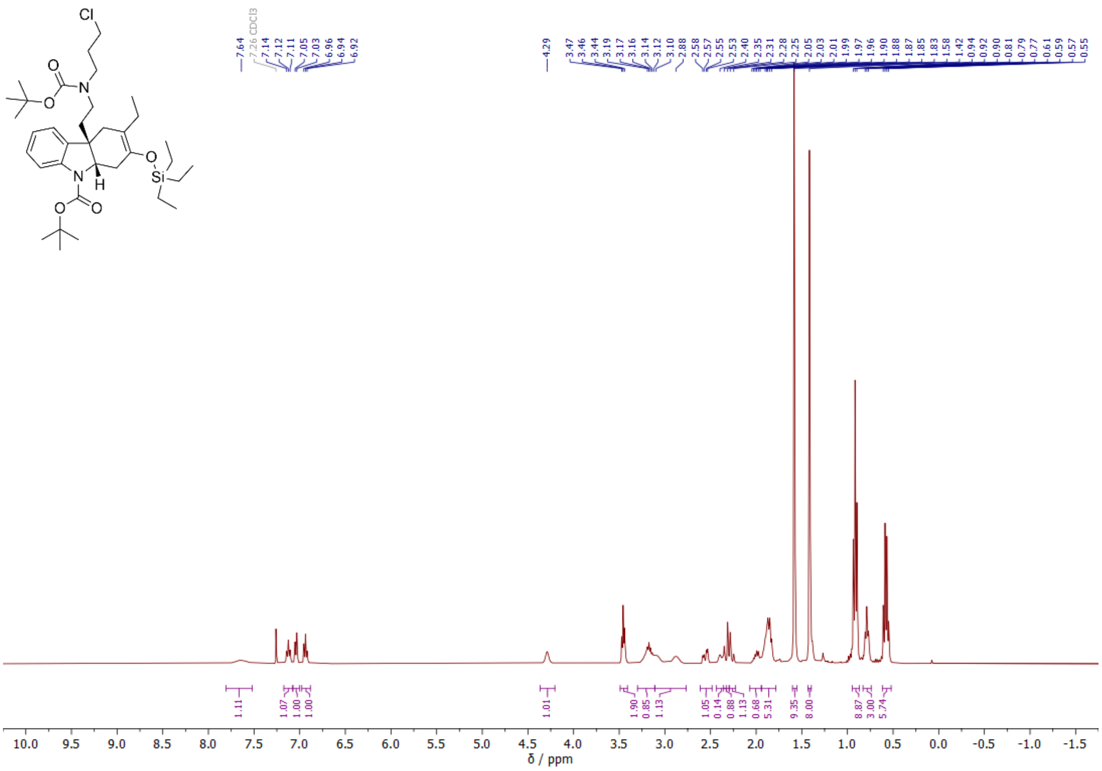

6.24 ^{13}C NMR Spectrum of Compound 13 (101 MHz, $CDCl_3$, 50 °C)

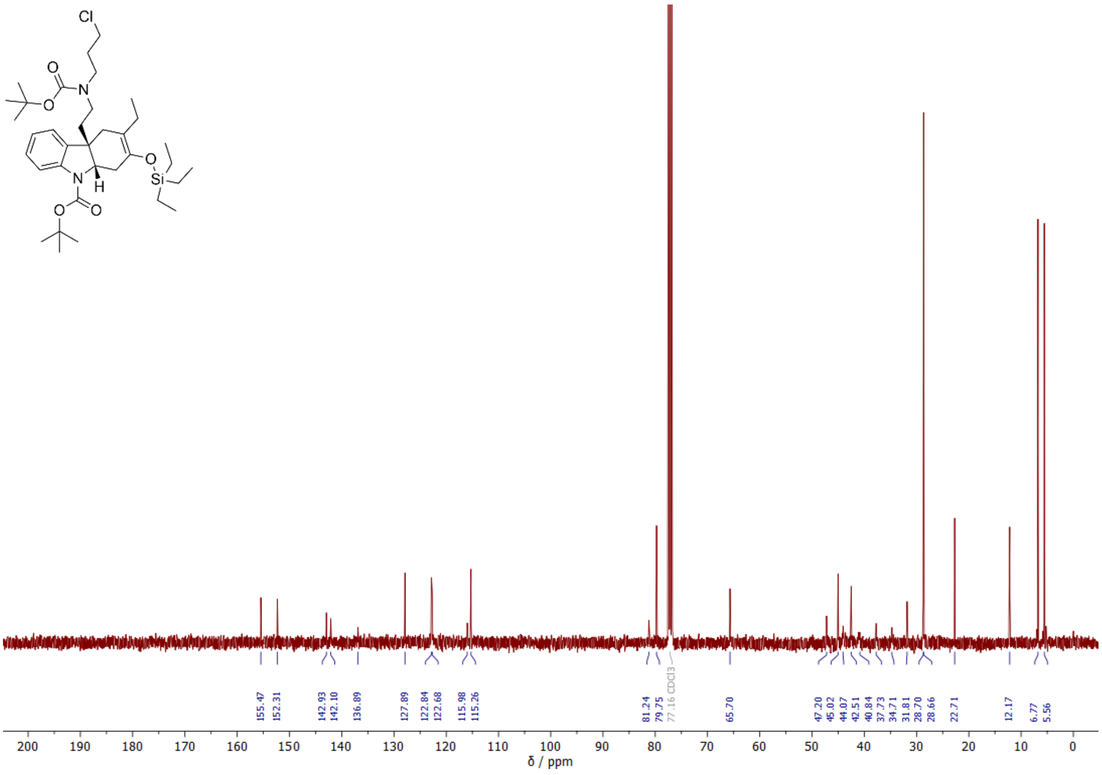

6.25 ^{1}H-^{13}C HSQC Spectrum of Compound 13 (CDCl$_3$, 50 °C)

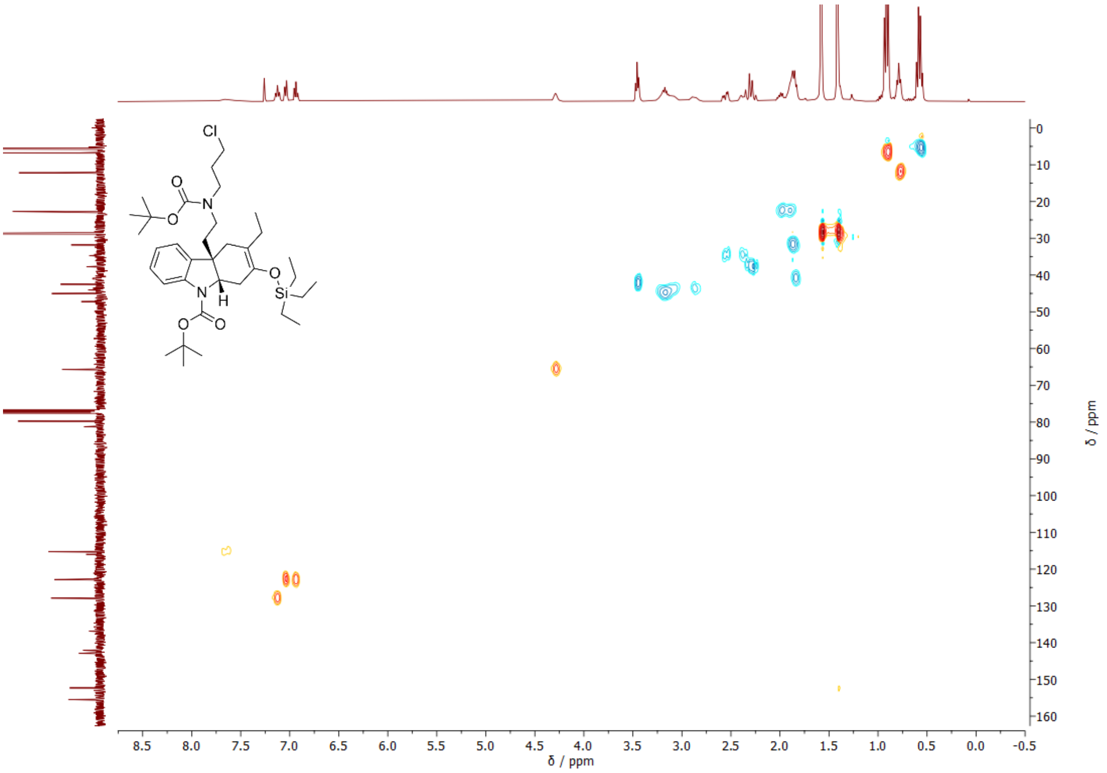

6.26 ^{1}H-^{13}C HMBC Spectrum of Compound 13 (CDCl$_3$, 50 °C)

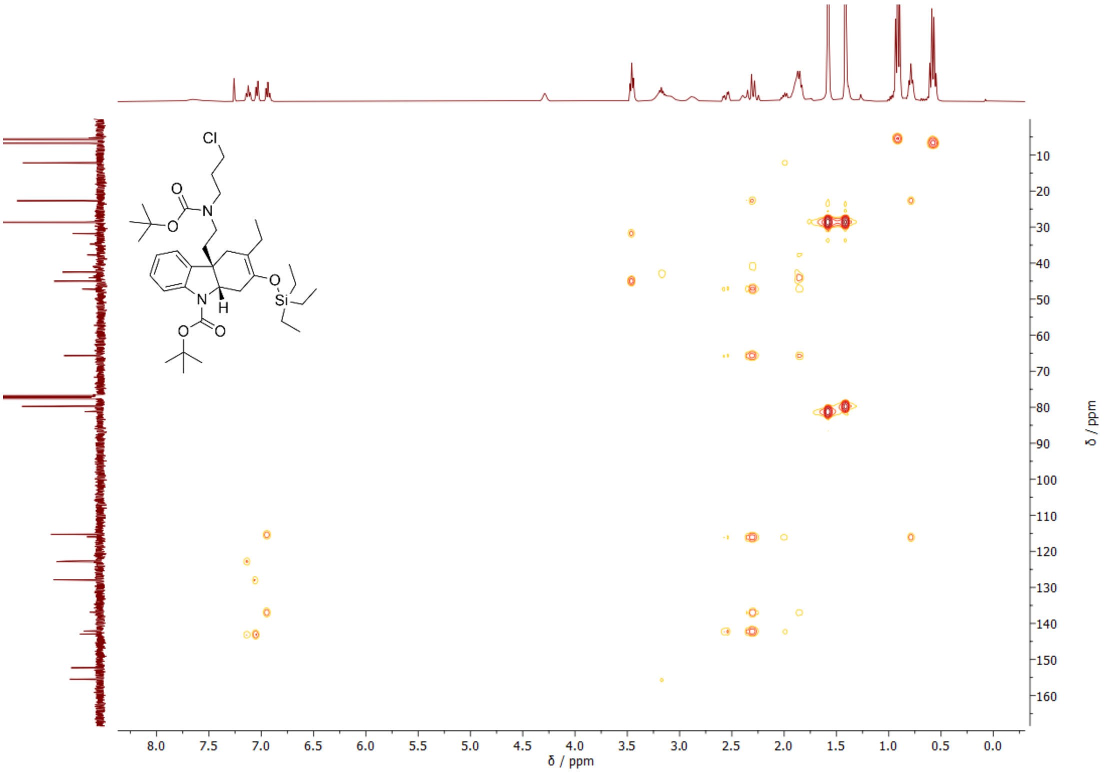

6.27 ^{1}H NMR Spectrum of Compound 14 (400 MHz, $CDCl_3$, 50 °C)

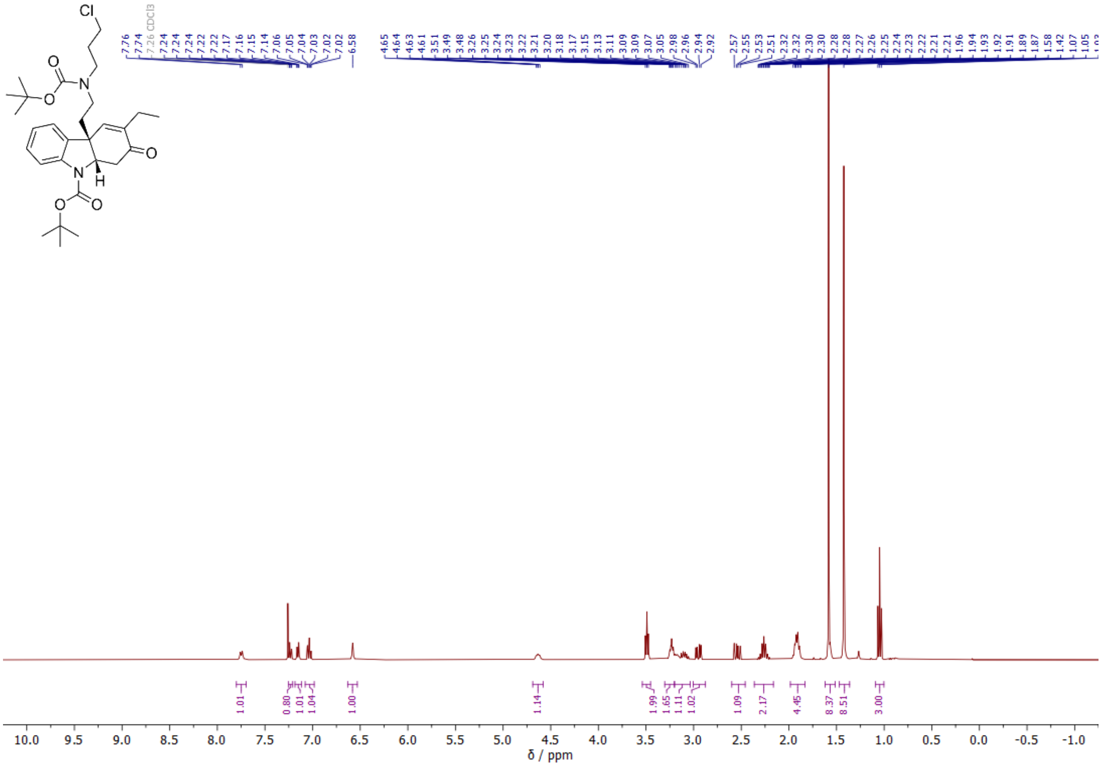

6.28 ^{13}C NMR Spectrum of Compound 14 (101 MHz, $CDCl_3$, 50 °C)

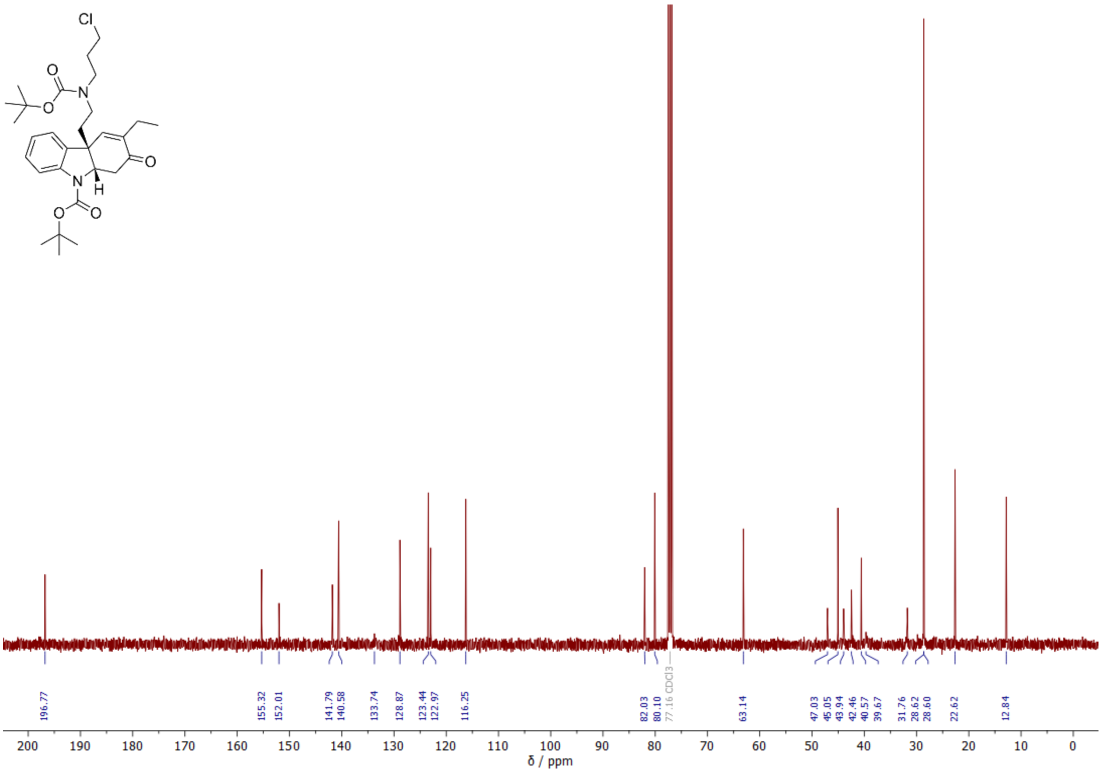

6.29 ^{1}H-^{13}C HSQC Spectrum of Compound 14 ($CDCl_3$, 50 °C)

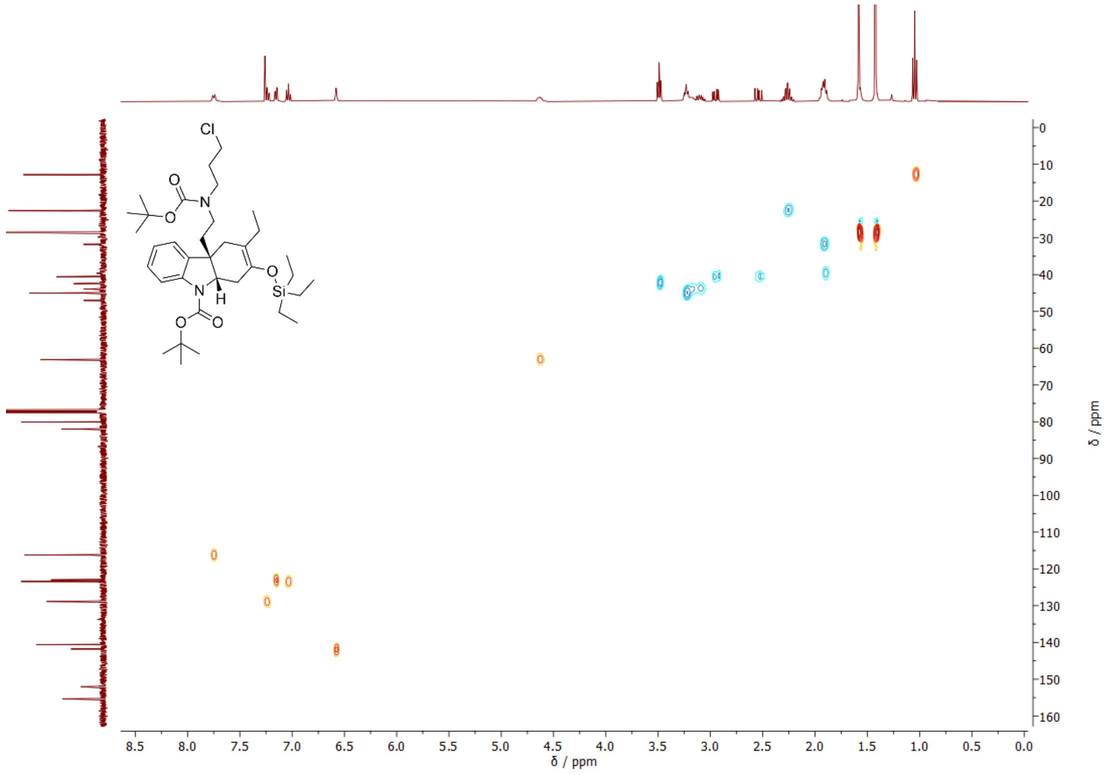

6.30 ^{1}H-^{13}C HMBC Spectrum of Compound 14 ($CDCl_3$, 50 °C)

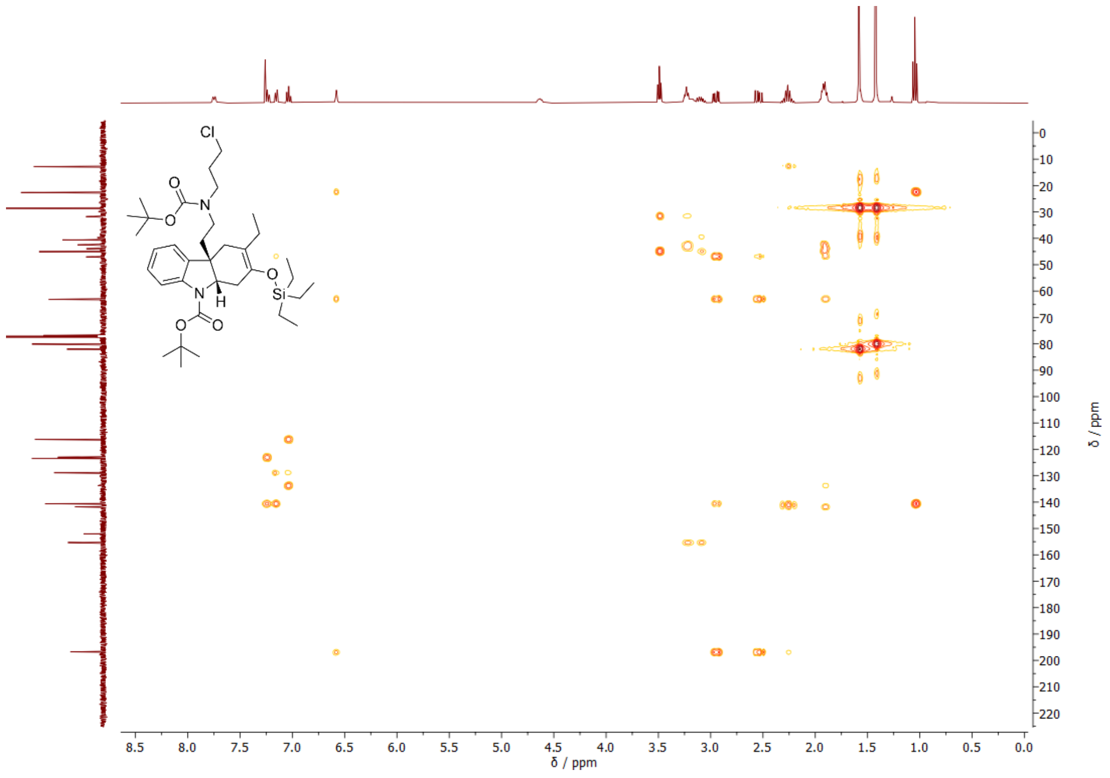

6.31 ^{1}H NMR Spectrum of Compound 15 (600 MHz, $CDCl_3$)

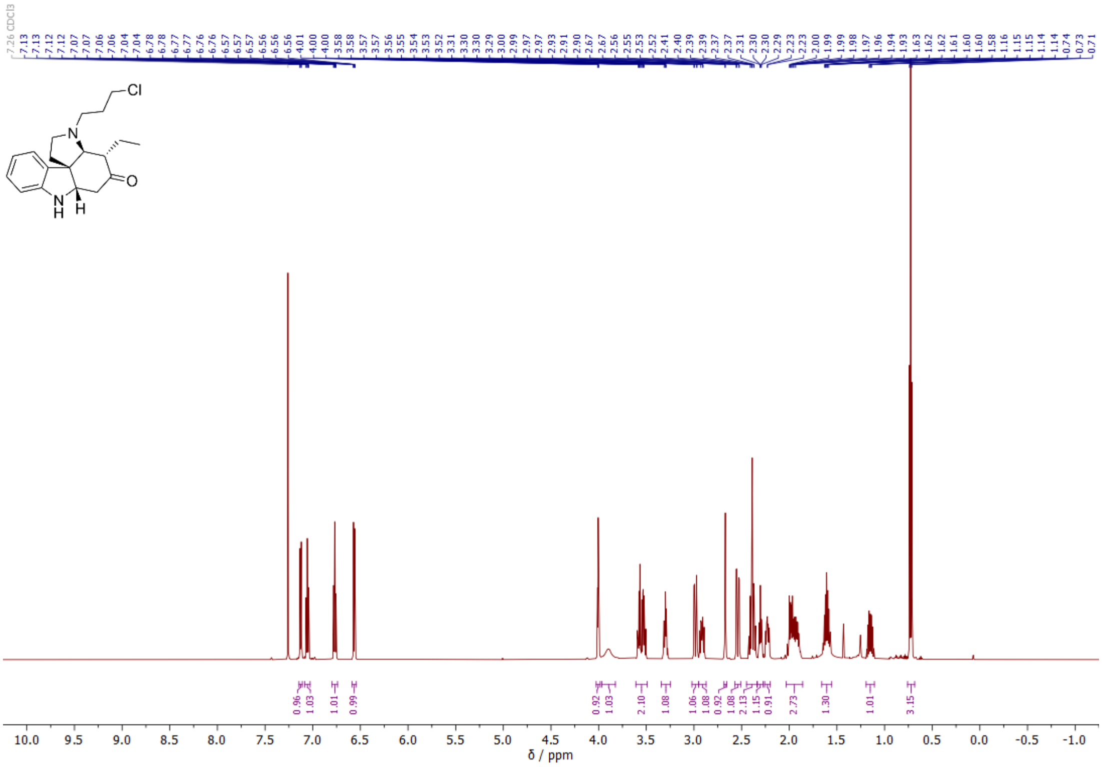

6.32 ^{13}C NMR Spectrum of Compound 15 (151 MHz, $CDCl_3$)

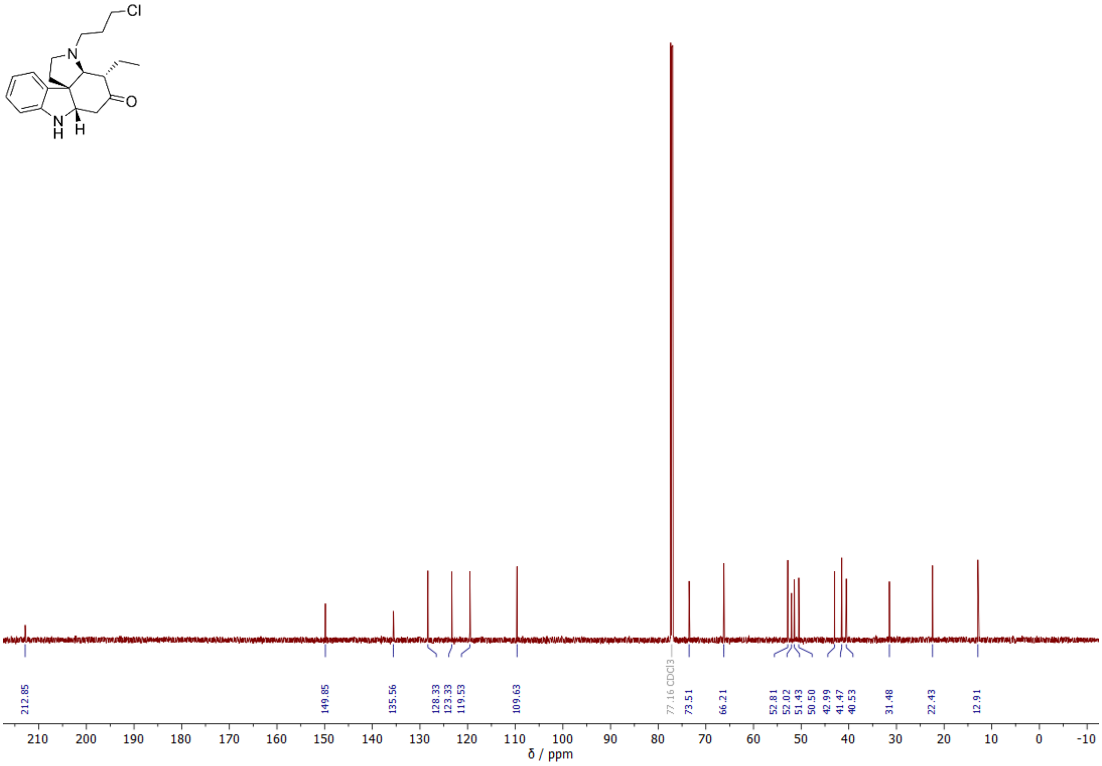

6.33 ^{1}H NMR Spectrum of Compound 16 (600 MHz, $CDCl_3$)

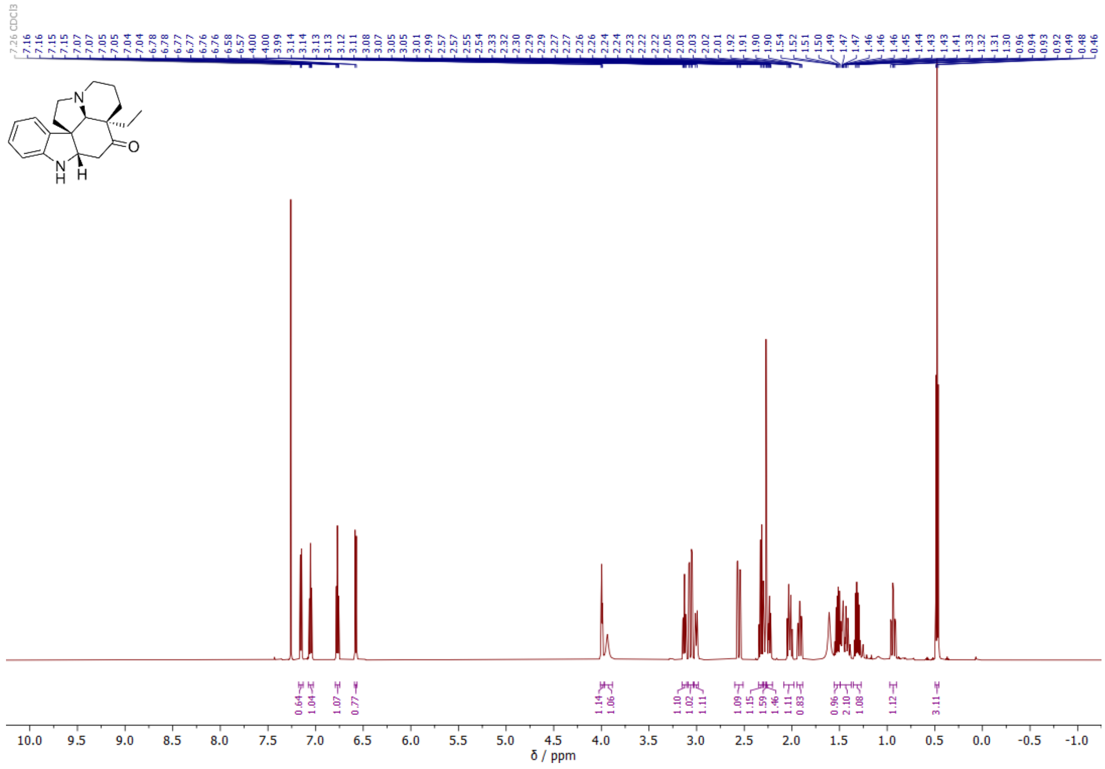

6.34 ^{13}C NMR Spectrum of Compound 16 (151 MHz, $CDCl_3$)

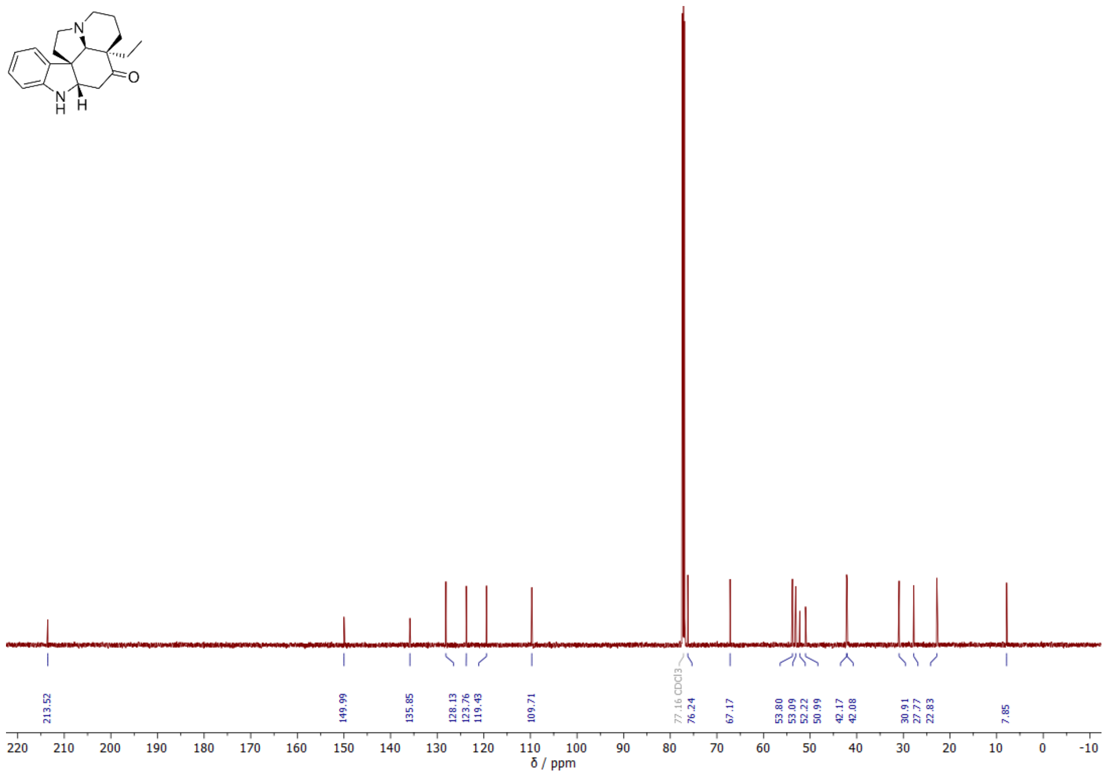

6.35 ^{1}H NMR Spectrum of Compound (−)-1 (600 MHz, $CDCl_3$)

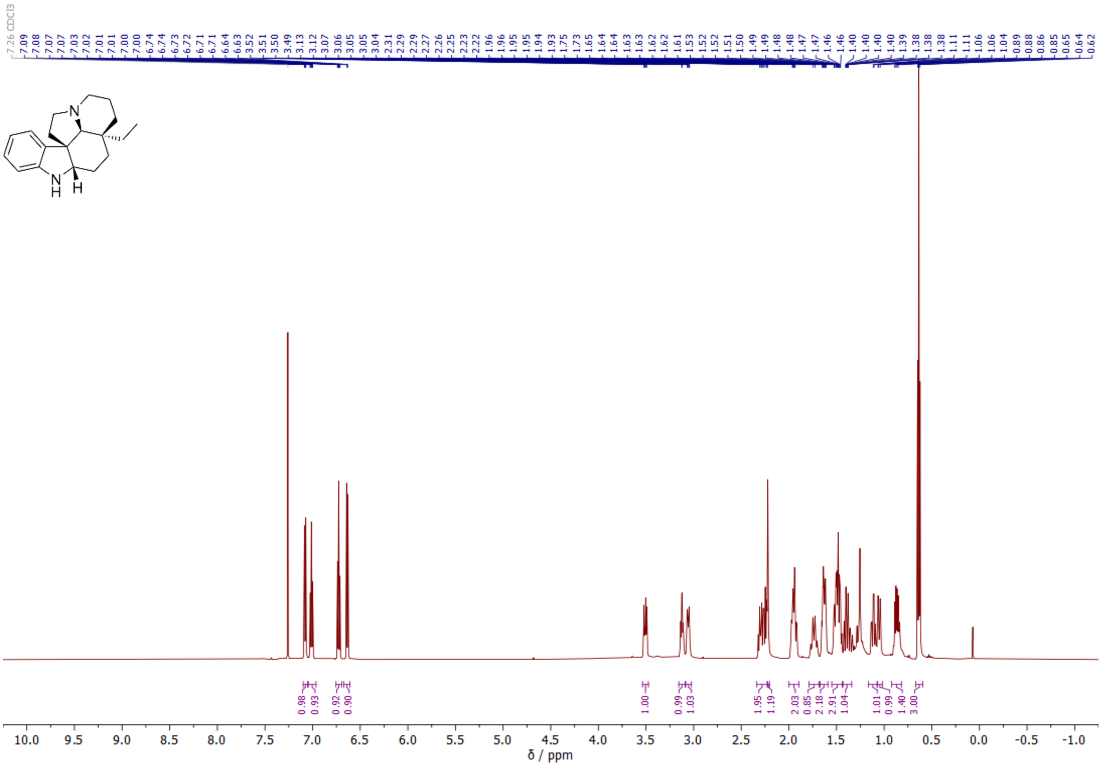

6.36 ^{13}C NMR Spectrum of Compound (−)-1 (151 MHz, $CDCl_3$)

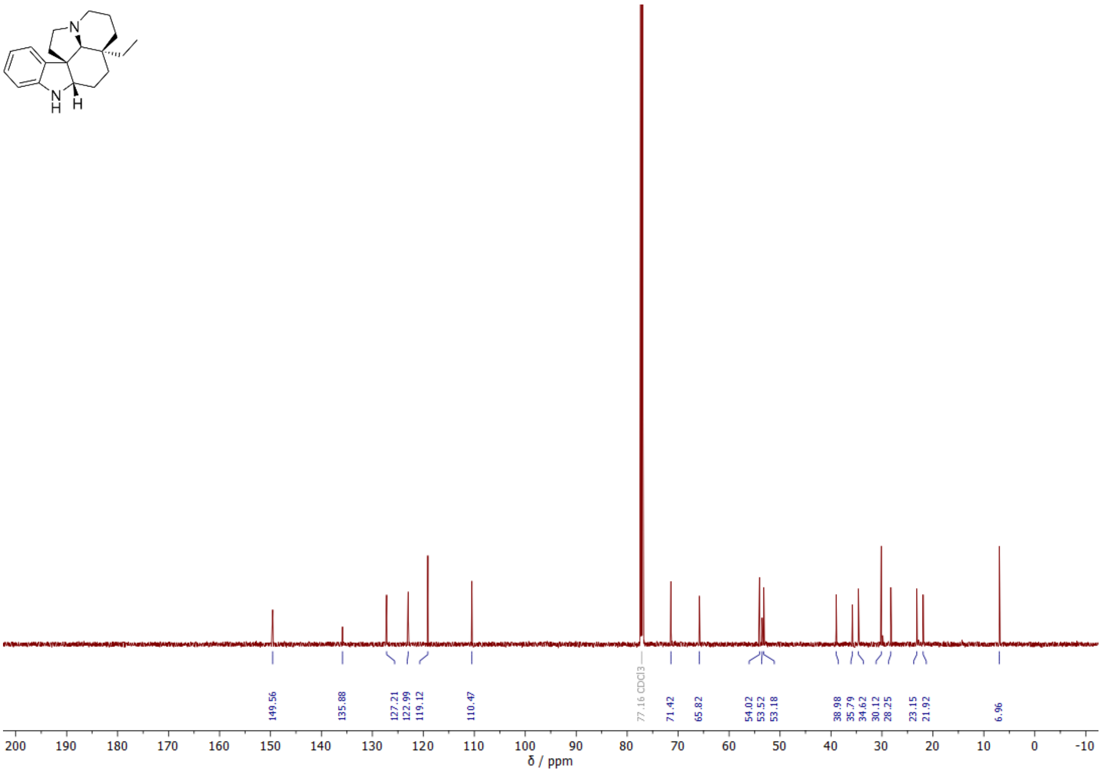

7 HPLC Chromatograms

7.1 HPLC Chromatogram of Racemic Compound 10

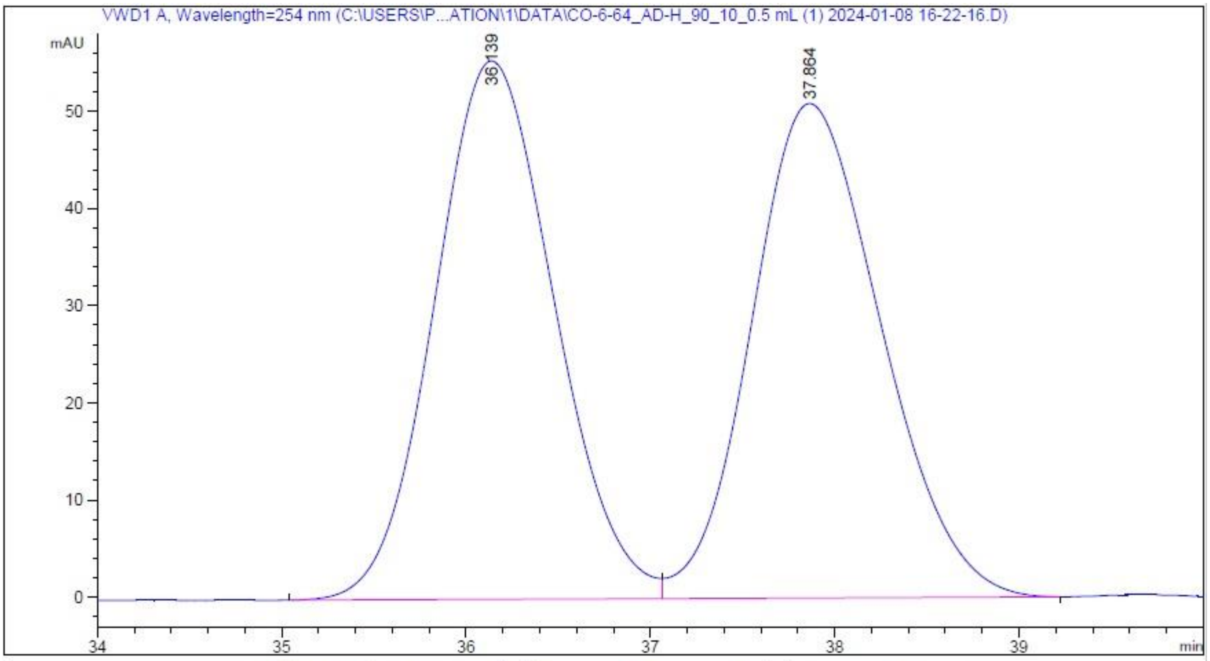

Peak #	RetTime [min]	Type	Width [min]	Area [mAU*s]	Height [mAU]	Area %
1	36.139	BV	0.5712	2499.11426	55.40646	50.1375
2	37.864	VB	0.5854	2485.40356	50.87529	49.8625

7.2 HPLC Chromatogram of Enantioenriched Compound 10

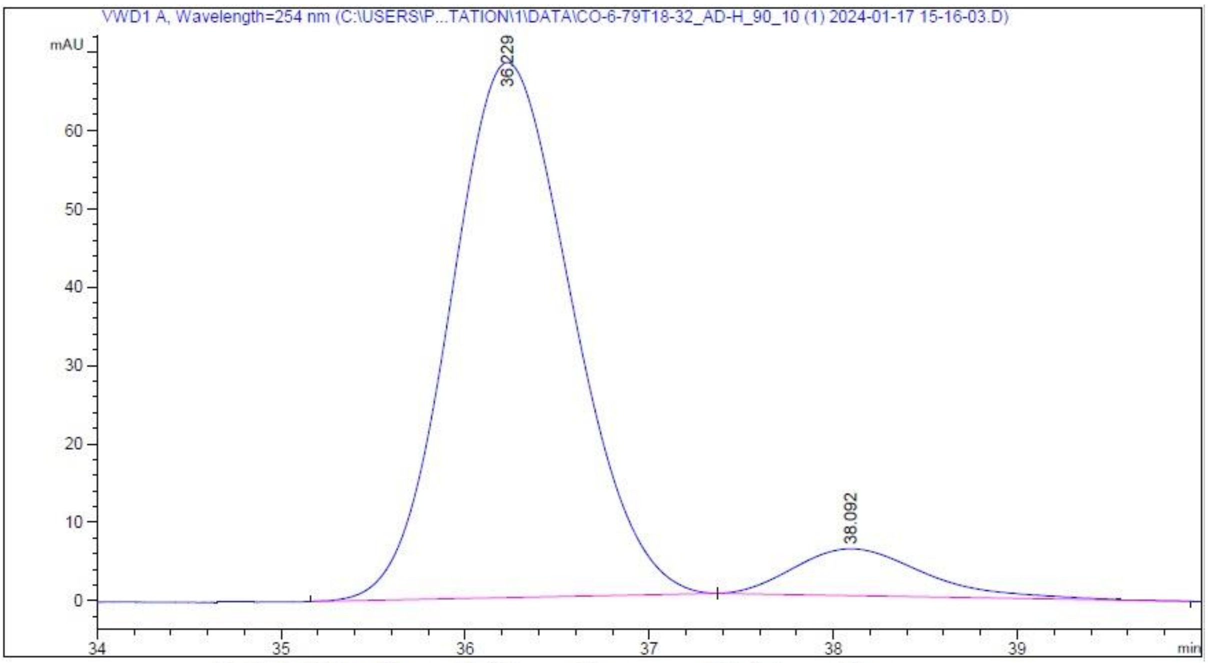

Peak #	RetTime [min]	Type	Width [min]	Area [mAU*s]	Height [mAU]	Area %
1	36.229	BB	0.5751	3109.92163	68.25481	91.3340
2	38.092	BBA	0.5791	295.07837	5.96056	8.6660

8 References

1. Kagawa, N.; Malerich, J. P.; Rawal, V. H., Palladium-Catalyzed β-Allylation of 2,3-Disubstituted Indoles. *Org. Lett.* **2008,** *10*, 2381-2384.
2. Kimura, M.; Futamata, M.; Mukai, R.; Tamaru, Y., Pd-Catalyzed C3-Selective Allylation of Indoles with Allyl Alcohols Promoted by Triethylborane. *J. Am. Chem. Soc.* **2005,** *127*, 4592-4593.
3. a) Liu, Y.; Du, H., Pd-Catalyzed Asymmetric Allylic Alkylations of 3-Substituted Indoles Using Chiral P/Olefin Ligands. *Org. Lett.* **2013,** *15*, 740-743; b) Tu, H.-F.; Zhang, X.; Zheng, C.; Zhu, M.; You, S.-L., Enantioselective Dearomative Prenylation of Indole Derivatives. *Nature Catalysis* **2018,** *1*, 601-608.
4. Müller, J. M.; Stark, C. B. W., Diastereodivergent Reverse Prenylation of Indole and Tryptophan Derivatives: Total Synthesis of Amauromine, Novoamauromine, and *epi*-Amauromine. *Angew. Chem. Int. Ed.* **2016,** *55*, 4798-4802.
5. Tanaka, M.; Mitsuhashi, H.; Maruno, M.; Wakamatsu, T., The Migration of Double Bond under the Neutral Conditions. The Transformation of α-Alkylidene Cyclic Carbonyl Compounds to α,β-Unsaturated Cyclic Carbonyl Compounds. *Chem. Lett.* **1994,** *23*, 1455-1458.
6. a) Diao, T.; Stahl, S. S., Synthesis of Cyclic Enones via Direct Palladium-Catalyzed Aerobic Dehydrogenation of Ketones. *J. Am. Chem. Soc.* **2011,** *133*, 14566-14569; b) Pan, G. F.; Zhu, X. Q.; Guo, R. L.; Gao, Y. R.; Wang, Y. Q., Synthesis of Enones and Enals via Dehydrogenation of Saturated Ketones and Aldehydes. *Adv. Synth. Catal.* **2018,** *360*, 4774-4783.
7. a) Nicolaou, K. C.; Zhong, Y. L.; Baran, P. S., A New Method for the One-Step Synthesis of α,β-Unsaturated Carbonyl Systems from Saturated Alcohols and Carbonyl Compounds. *J. Am. Chem. Soc.* **2000,** *122*, 7596-7597; b) Nicolaou, K. C.; Gray, D. L.; Montagnon, T.; Harrison, S. T., Oxidation of Silyl Enol Ethers by Using IBX and IBX•*N*-Oxide Complexes: A Mild and Selective Reaction for the Synthesis of Enones. *Angew. Chem. Int. Ed.* **2002,** *41*, 996-1000.
8. List, B.; Shevchenko, G.; Dehn, S., Brønsted Acid Mediated Direct α-Hydroxylation of Cyclic α-Branched Ketones. *Synlett* **2018,** *29*, 2298-2300.
9. a) Cazeau, P.; Duboudin, F.; Moulines, F.; Babot, O.; Dunogues, J., A New Practical Synthesis of Silyl Enol Ethers. *Tetrahedron* **1987,** *43*, 2075-2088; b) Bruckner, R., In *Organic Mechanisms: Reactions, Stereochemistry and Synthesis*, 3 ed.; Harmata, M., Ed. Springer: Berlin, Heidelberg, 2010; pp 531-532.
10. Anada, M.; Tanaka, M.; Suzuki, K.; Nambu, H.; Hashimoto, S., Dirhodium(II) Tetrakis(perfluorobutyrate)-Catalyzed 1,4-Hydrosilylation of α,β-Unsaturated Carbonyl Compounds. *Chem. Pharm. Bull.* **2006,** *54*, 1622-1623.
11. a) Ito, Y.; Hirao, T.; Saegusa, T., Synthesis of α,β-Unsaturated Carbonyl Compounds by Palladium(II)-Catalyzed Dehydrosilylation of Silyl Enol Ethers. *J. Org. Chem.* **1978,** *43*, 1011-1013; b) Gross, R. S.; Grieco, P. A.; Collins, J. L., Synthetic Studies on Quassinoids: Total Synthesis of (±)-Chaparrinone. *J. Am. Chem. Soc.* **2002,** *112*, 9436-9437; c) Angeles, A. R.; Waters, S. P.; Danishefsky, S. J., Total Syntheses of (+)- and (-)-Peribysin E. *J. Am. Chem. Soc.* **2008,** *130*, 13765-13770; d) Larock, R. C.; Hightower, T. R.; Kraus, G. A.; Hahn, P.; Zheng, D., A Simple, Effective, New, Palladium-Catalyzed Conversion of Enol Silanes to Enones and Enals. *Tetrahedron Lett.* **1995,** *36*, 2423-2426.
12. Zheng, W.; Cole, P. A., Novel Bisubstrate Analog Inhibitors of Serotonin *N*-Acetyltransferase: the Importance of Being Neutral. *Bioorg. Chem.* **2003,** *31*, 398-411.
13. Konig, C. M.; Harms, K.; Koert, U., Stereoselective Synthesis of Methyl 7-Dihydro-trioxacarcinoside B. *Org. Lett.* **2007,** *9*, 4777-4779.

14. Yang, C. T.; Zhang, Z. Q.; Liu, Y. C.; Liu, L., Copper-Catalyzed Cross-Coupling Reaction of Organoboron Compounds with Primary Alkyl Halides and Pseudohalides. *Angew. Chem. Int. Ed.* **2011,** *50*, 3904-3907.

15. Frigerio, M.; Santagostino, M.; Sputore, S., A User-Friendly Entry to 2-Iodoxybenzoic Acid (IBX). *J. Org. Chem.* **1999,** *64*, 4537-4538.

16. Frigerio, M.; Santagostino, M., A Mild Oxidizing Reagent for Alcohols and 1,2-Diols: o-iodoxybenzoic acid (IBX) in DMSO. *Tetrahedron Lett.* **1994,** *35*, 8019-8022.

17. Marino, J. P.; Rubio, M. B.; Cao, G.; de Dios, A., Total Synthesis of (+)-Aspidospermidine: A New Strategy for the Enantiospecific Synthesis of *Aspidosperma* alkaloids. *J. Am. Chem. Soc.* **2002,** *124*, 13398-13399.

18. Wenkert, E.; Hudlicky, T., Synthesis of Eburnamonine and Dehydroaspidospermidine. *J. Org. Chem.* **1988,** *53*, 1953-1957.

19. He, Y.-L.; Chen, W.-M.; Feng, X.-Z., Melomorsine, A New Dimeric Indoline Alkaloid from *Melodinus Morsei*. *J. Nat. Prod.* **2004,** *57*, 411-414.
